# Fundamental Cell Morphologies Examined With Cryo-TEM of the Species in the Novel Five Genera Robustly Correlate With New Classification in Family *Mycobacteriaceae*

**DOI:** 10.3389/fmicb.2020.562395

**Published:** 2020-11-16

**Authors:** Hiroyuki Yamada, Kinuyo Chikamatsu, Akio Aono, Kazuyoshi Murata, Naoyuki Miyazaki, Yoko Kayama, Apoorva Bhatt, Nagatoshi Fujiwara, Shinji Maeda, Satoshi Mitarai

**Affiliations:** ^1^Department of Mycobacterium Reference and Research, The Research Institute of Tuberculosis, Japan Anti-Tuberculosis Association, Tokyo, Japan; ^2^Supportive Center for Brain Research, National Institute for Physiological Science, Okazaki, Japan; ^3^Life Science Center for Survival Dynamics, Tsukuba Advanced Research Alliance (TARA), University of Tsukuba, Tsukuba, Japan; ^4^Terabase Inc., Okazaki, Japan; ^5^School of Biosciences and Institute of Microbiology and Infection, University of Birmingham, Birmingham, United Kingdom; ^6^Department of Food and Nutrition, Faculty of Contemporary Human Life Science, Tezukayama University, Nara, Japan; ^7^Department of Pharmacy, Faculty of Pharmaceutical Science, Hokkaido University of Science, Sapporo, Japan; ^8^Department of Basic Mycobacteriology, Graduate School of Biomedical Sciences, Nagasaki University, Nagasaki, Japan

**Keywords:** family *Mycobacteriaceae*, cryo-TEM examination, single cell morphology, cell length, cell diameter, circularity, cell perimeter, aspect ratio

## Abstract

A recent study proposed the novel classification of the family *Mycobacteriaceae* based on the genome analysis of core proteins in 150 *Mycobacterium* species. The results from these analyses supported the existence of five distinct monophyletic groups within the genus *Mycobacterium*. That is, *Mycobacterium* has been divided into two novel genera for rapid grower *Mycobacteroides* and *Mycolicibacterium*, and into three genera for slow grower *Mycolicibacter*, *Mycolicibacillus*, and an emended genus *Mycobacterium*, which include all the major human pathogens. Here, cryo-TEM examinations of 1,816 cells of 31 species (34 strains) belonging to the five novel genera were performed. The fundamental morphological properties of every single cell, such as cell diameter, cell length, cell perimeter, cell circularity, and aspect ratio were measured and compared between these genera. In 50 comparisons on the five parameters between any two genera, only five comparisons showed “non-significant” differences. That is, there are non-significant differences between slow grower genus *Mycolicibacillus* and genus *Mycobacterium* in average cell diameter (*p* = 0.15), between rapid grower genus *Mycobacteroides* and slow grower genus *Mycobacterium* in average cell length (*p* > 0.24), between genus *Mycobacteroides* and genus *Mycobacterium* (*p* > 0.68) and between genus *Mycolicibacter* and genus *Mycolicibacillus* (*p* > 0.11) in average cell perimeter, and between genus *Mycolicibacterium* and genus *Mycobacterium* in circularity (*p* > 0.73). The other 45 comparisons showed significant differences between the genera. Genus *Mycobacteroides* showed the longest average cell diameter, whereas the genus *Mycolicibacter* showed the shortest average diameter. Genus *Mycolicibacterium* showed the most extended average cell length, perimeter, and aspect ratio, whereas the genus *Mycolicibacillus* showed the shortest average cell length, perimeter, and aspect ratio. Genus *Mycolicibacillus* showed the highest average cell circularity, whereas genus *Mycobacterium* showed the lowest average cell circularity. These fundamental morphological data strongly support the new classification in the family *Mycobacteriaceae*, and this classification is rational and effective in the study of the members of the family *Mycobacteriaceae*. Because both the genus *Mycolicibacterium* and the genus *Mycobacterium* contain many species and showed larger significant standard deviations in every parameter, these genera may be divided into novel genera which show common genotype and phenotypes in morphology and pathogenicity.

## Introduction

*Mycobacterium* is often described as “0.2–0.6 μm by 1.0–10.0 μm, straight or slightly curved rod-shaped bacteria, sometimes branched, acid-fast.” Methods of measuring cell morphology include light microscopic examination of stained bacilli or electron microscopy of transmission or scanning. However, both have disadvantages to measure precise values, such as diameter or length, because the resolution power of general light microscopy is approximately 0.2 μm, which is as large as one-third of the estimated cell diameter, and because measurements with conventional transmission electron microscopy (TEM) and scanning electron microscopy (SEM) examination resulted in smaller values due to the shrinkage of samples during the preparation process due to dehydration and drying (Yamada et al., 2017).

In recent studies on the cell morphology of mycobacteria, bacteria cells were often examined with live time-lapse fluorescent microscopy and the lengths of some cells were measured with microfluidic culture systems (Aldridge et al., 2012; Joyce et al., 2012; Santi et al., 2013; Singh et al., 2013; Wakamoto et al., 2013). Another study measured cell length using a combination of transmission electron microscopy, fluorescent, and light microscopy (Vijay et al., 2014). The same authors examined *Mycobacterium tuberculosis* (MTB) clinical isolates stained Ziehl-Neelsen and analyzed cell length diversity, which varied from cell to cell (Vijay et al., 2017). However, data obtained from these studies were less precise because of the reasons mentioned above and they rarely measured cell diameters (Milo et al., 2010). This study conducted whole-mount ice-embedded cryo-TEM examinations of more than 1,800 cells of species belonging to the family *Mycobacteriaceae*, obtaining precise cell morphology data on the cell length, cell diameter, cell perimeter, cell circularity, and aspect ratio.

In 2018, the family *Mycobacteriaceae* was classified into five genera: the rapidly growing genera *Mycobacteroides* and *Mycolicibacterium*, and slow-growing genera *Mycolicibacter*, *Mycolicibacillus*, and *Mycobacterium*, which contain many highly pathogenic species, based on a comparison of core proteins, the average amino acid identity of the conserved protein families, the molecular signatures in the form of conserved signature indels, and conserved signature proteins (Gupta et al., 2018; Oren and Garrity, 2018a,b). Our previous studies reported on a structome analysis of MTB and *Mycolicibacterium smegmatis* (MSG, basonym *Mycobacterium smegmatis*), which revealed that non-pathogenic MSG is entirely distinct from virulent MTB (Yamada et al., 2015, 2018). However, MSG has been often used as an alternative to MTB, but MSG is a distinct species in a genus different from MTB (Yamada et al., 2018). The present study was based on the novel classification and comparison of fundamental cell morphology data obtained from cryo-TEM examination between the five genera. Most comparisons of the cell length, the cell diameter, the cell perimeter, the cell circularity, and the aspect ratio revealed significant differences between the five genera. Therefore, these data suggest and support the idea that the novel classification of the family *Mycobacteriaceae* is proper and reasonable from the viewpoint of cell morphology and the ultimate phenotype.

## Materials and Methods

### Bacteria

Bacterial strains examined in this study were purchased from ATCC, JCM, or DSM, except for the strain CDC1551 (Bhatt et al., 2007; Yamada et al., 2012), as listed in Tables 1–3. These bacteria were cultured with 50 ml Middlebrook 7H9 (Becton Dickinson, Sparks, MD, United States) and supplemented with OADC enrichment (oleic acid, bovine albumin (Fraction V), dextrose, and catalase, Becton Dickinson), and 0.05% Tween 80 (Sigma-Aldrich), contained in a 125-ml Erlenmeyer flask with a flat bottom (Nalgene, 4112-0125, NY, United States) or with Mycobroth (Kyokuto Pharmaceutical Industrial Co. Ltd., Tokyo, Japan).

**TABLE 1:** List of rapid grower species and strains in genus *Mycobacteroides* and genus *Mycolicibacterium* examined with cryo-TEM.

Species (strains)	Strain identification number	Number of examined cell	Type strain
**Rapid growers**			
**Genus *Mycobacteroides***			
*Mycobacteroides abscessus* subsp. *abscessus*	ATCC 19977	82	Yes
*Mycobacteroides abscessus* subsp. *bolletii*	JCM 15297	158	Yes
*Mycobacteroides abscessus* subsp. *massiliense*	JCM 15300	149	Yes
*Mycobacteroides chelonae*	ATCC 35752	27	Yes
*Mycobacteroides immunogenum*	DSM 45595	31	Yes
*Mycobacteroides salmoniphilum*	DSM 43276	65	Yes
Subtotal		512	
**Genus *Mycolicibacterium***			
*Mycolicibacterium aurum*	ATCC 23366	36	Yes
*Mycolicibacterium austroafricanum*	ATCC 33464	13	Yes
*Mycolicibacterium chitae*	ATCC 19627	20	Yes
*Mycolicibacterium fortuitum* subsp. *fortuitum*	ATCC 06841	98	Yes
*Mycolicibacterium fortuitum* subsp. *fortuitum*	ATCC 11440	18	No
*Mycolicibacterium gilvum*	ATCC 43909	51	Yes
*Mycolicibacterium smegmatis*	ATCC 19420	61	Yes
Subtotal		297	
Total		809	

**TABLE 2:** List of slow grower species and strains in genus *Mycolicibacter* and genus *Mycolicibacillus* examined with cryo-TEM.

Species (strains)	Strain identification number	Number of examined cell	Type strain
**Slow growers**			
**Genus *Mycolicibacter***			
*Mycolicibacter algericus*	DSM 45454	38	Yes
*Mycolicibacter non chromogenicus*	ATCC19530	42	Yes
*Mycolicibacter terrae*	ATCC 15755	45	Yes
Subtotal		125	
**Genus *Mycolicibacillus***			
*Mycolicibacillus koreensis*	DSM 45576	30	Yes
*Mycolicibacillus parakoreensis*	DSM 45575	11	Yes
*Mycolicibacillus trivialis*	ATCC 23292	27	Yes
Subtotal		68	
Total		193	

**TABLE 3:** List of slow grower species and strains in genus *Mycobacterium* examined with cryo-TEM.

Species (strains)	Strain identification number	Number of examined cell	Type strain
**Slow growers**			
**Genus *Mycobacterium***			
*Mycobacterium tuberculosis* H37Rv	ATCC 27294	14	Yes
*Mycobacterium tuberculosis* CDC1551	Bhatt et al., 2007	29	No
*Mycobacterium africanum*	ATCC 25420	63	Yes
*Mycobacterium bovis*	ATCC 19210	134	Yes
*Mycobacterium microti*	ATCC 19422	28	Yes
*Mycobacterium avium*	ATCC 25291	62	Yes
*Mycobacterium celatum*	ATCC 51130	37	No
*Mycobacterium celatum*	ATCC 51131	25	Yes
*Mycobacterium gordonae*	ATCC 14470	43	Yes
*Mycobacterium intermedium*	ATCC 51848	43	Yes
*Mycobacterium intracellulare*	ATCC 13950	125	Yes
*Mycobacterium marinum*	ATCC 00927	38	Yes
*Mycobacterium scrofulaceum*	ATCC 19981	60	Yes
*Mycobacterium ulcerans*	ATCC 19423	11	Yes
*Mycobacterium xenopi*	ATCC 19250	102	Yes
Total		814	

### Preparation of Bacterial Suspensions for Whole-Mount Ice-Embedded CryoTEM Examination

The cells were then used in the exponential growth phase. Aliquots (4 ml) of cultured cells were transferred to two 2 ml sterile microcentrifuge tubes, which were treated with SigmaCoat (Sigma-Aldrich, SL-2, MO, United States) to prevent bacterial cells from adhering to the tube wall, and centrifuged at 10,000 × g for 1 min. The supernatants were discarded, and the sediments were resuspended in 2 ml of 2.5% glutaraldehyde in phosphate buffer (PB, 0.1M, pH7.4) and collected in one microcentrifuge tube. Then the bacterial cells were fixed at 4°C overnight. Then, the bacterial suspensions were centrifuged at 10,000 × g, and the supernatants were discarded and rinsed with PB. After centrifugation at 10,000 × g and the supernatants were discarded. Finally, the sediments were resuspended with 200 μl PB, and the concentrated bacterial suspensions were filtered with Acrodisk 32 mm Syringe Filter with 5.0 μm Supor Membrane (PALL Life Sciences, REF4650, United Kingdom) to obtain single-cell suspensions. The final bacterial solutions were stored at 4°C until use. Manipulattions of the bacterial samples before fixation with glutaraldehyde were performed in the biosafety level 3 facility.

### Whole-Mount Ice-Embedded CryoTEM Examination

1 μL of the bacterial suspension prepared above was applied to a glow-discharged carbon grid with holes (Quantifoil copper grids R 2/1 or S 7/2, Quantifoil MicroTools, Jena, Germany) and mounted in an environmentally controlled chamber at 100% humidity, excess water was removed by blotting, and the grids were frozen in vitreous ice by plunging them into liquid ethane or ethane-propane mixture cooled with liquid nitrogen using a Vitrobot Mark II (FEI, Hillsboro, OR, United States) or EM GP2 (Leica Mikrosysteme GmbH, Vienna, Austria). The grid was loaded in a Single Tilt Liquid Nitrogen Cryo Transfer holder (Model 626, Gatan Inc., Pleasanton, CA, United States) and transferred into TEM.

The microscope was operated at 300 kV (JEM-3100, JEOL, Tokyo, Japan), 200 kV (JEM-2200FS and JEM-2100Plus, JEOL, Tokyo, Japan) or 120 kV (JEM-1230 and JEM-2100Plus, JEOL, Tokyo, Japan) acceleration voltages, and the samples on the grids were cooled during examination with liquid nitrogen in the single tilt nitrogen cryotransfer holder described above at −175.9 to −177.8°C. Raw images of the intact cells were recorded at the magnification of ×6,000, ×8,000, or ×10,000 according to the length of a single cell on a 4K × 4K charge-coupling device (CCD) sensor (F415, TVPIS, Germany) with JEM-3100 and JEM-2200FS, on 1K × 1K CCD digital camera system (OSIS MegaView G2, Olympus, Tokyo, Japan) with JEM-1230, or 2K × 2K high sensitivity CMOS camera system (JEOL, Tokyo, Japan) with JEM-2100Plus. Damaged or bent cells and the cells embedded in the thicker ice were not examined because the exact cell morphology could not be obtained (Yamada et al., 2012, 2018).

### Image Analysis

The captured images were saved as TIFF files, except for the JEM-3100 examination, with which images were captured as dm3 files and converted into TIFF files using ImageJ (Schindelin et al., 2012) plug-in. The cell profiles, which can be traced whole cell outline clearly, were analyzed using the Measure command in the Analyze menu of ImageJ/Fiji. Briefly, the cell diameter and cell length of each cell were measured using the line selection menu, and cell perimeter, circularity, and the aspect ratio of each cell was measured by tracing the outermost profile of each cell using the polygonal selection menu in the ImageJ window. Measured pixel values were converted to μm or nm according to the measured pixel value of the scale bar on the corresponding images. In cell length analysis, the shortest and the longest values were recorded, and the longest/the shortest ratios were calculated in each species (each strain). All measures were recorded in Fiji, exported as CSV files, and analyzed with Microsoft^®^ Excel for Mac Ver. 13.36.

### Statistics

Compare mean *t*-test, and one-way ANOVA was performed to compare the differences in mean values. Statistical analyses were performed using StatPlus: mac, AnalystSoft Inc.—statistical analysis program for macOS^®^. Version v7 (StatPlus Mac, RRID:SCR_014635).

## Results

### Cryo-TEM Images

**FIGURE 1:**
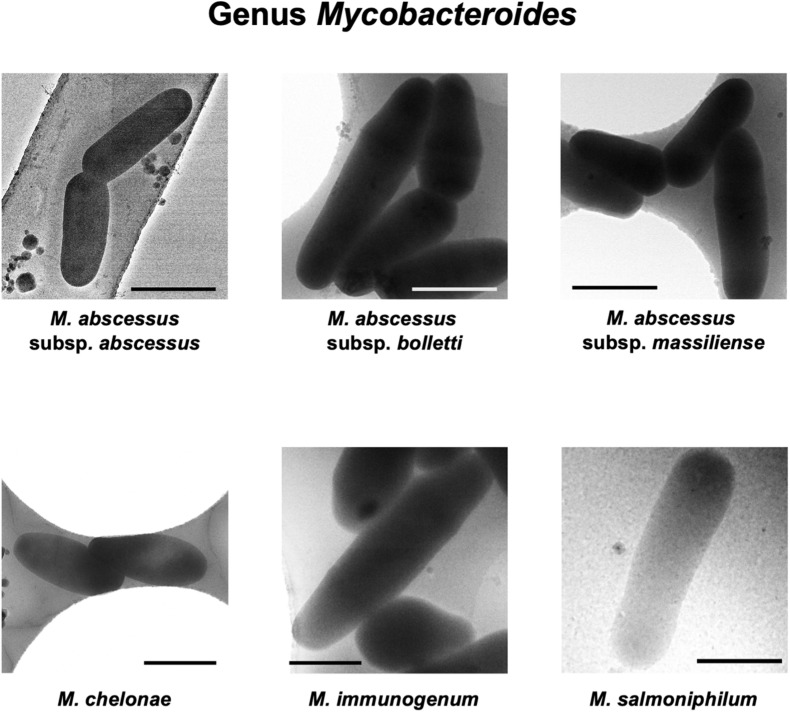
Representative cryo-TEM images of the cells of rapidly growing species belonging to genus *Mycobacteroides*; *M. abscessus* subsp. *abscessus*, *M. abscessus* subsp. *bolletti*, *M. abscessus* subsp. *massiliense*, *M. chelonae*, *M. immunogenum*, and *M. salmoniphilum*. Each scale bar indicates 1 μm.

**FIGURE 2:**
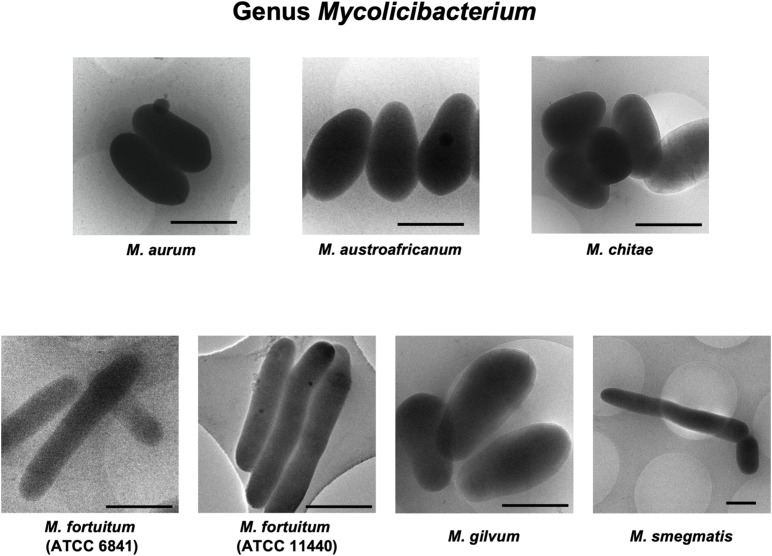
Representative cryo-TEM images of the cells of rapidly growing species belonging to genus *Mycolicibacterium*; *M. aurum*, *M. austroafricanum*, *M. chitae*, *M. fortuitum* (ATCC 6841), *M. fortuitum* (ATCC 11440), *M. gilvum*, and *M. smegmatis*. Each scale bar indicates 1 μm.

**FIGURE 3:**
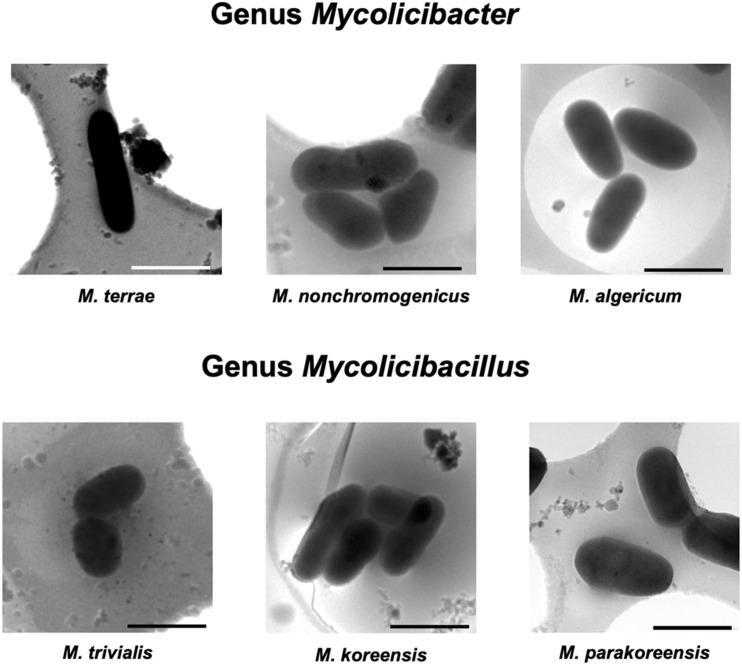
Representative cryoTEM images of the cells of slow-growing species belonging to genus *Mycolicibacter*, *M. terrae*, *M. non chromogenicus*, *M. algericum*, *and genus Mycolicibacillus*, *M. trivialis*, *M. koreensis*, *M. parakoreensis*. Each scale bar indicates 1 μm.

**FIGURE 4:**
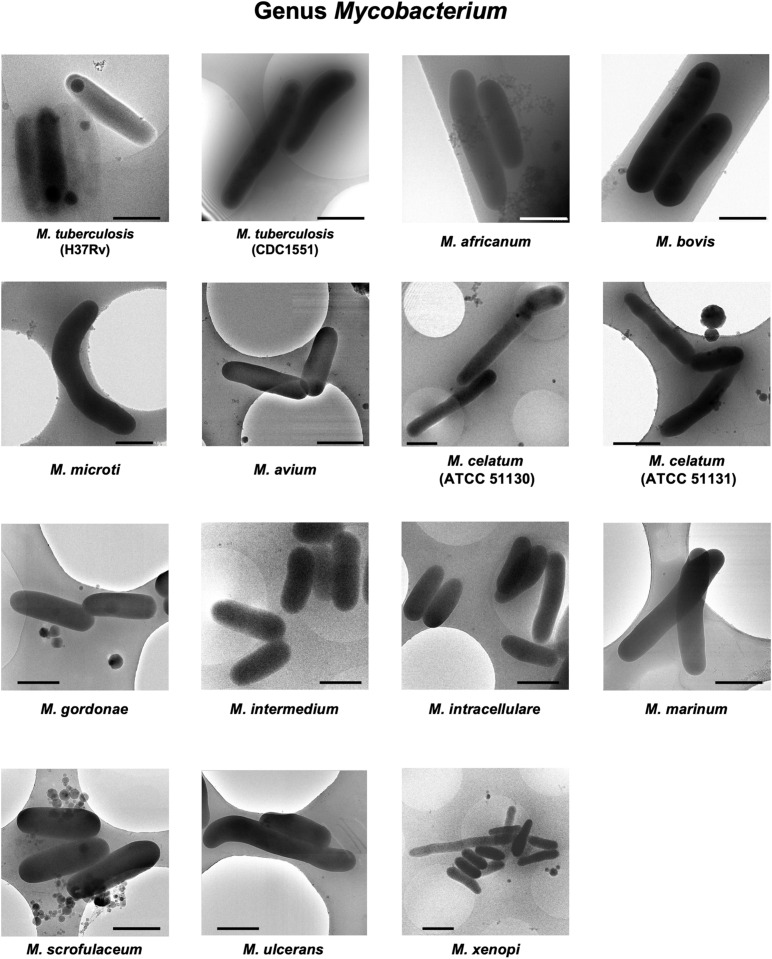
Representative cryo-TEM images of the cells of slow-growing species belonging to genus *Mycobacterium*, *M. tuberculosis* (H37Rv), *M. tuberculosis* (CDC1551), *M. africanum*, *M. bovis*, *M. microti*, *M. avium*, *M. celatum* (ATCC 51130), *M. celatum* (ATCC 51131), *M. gordonae*, *M. intermedium*, *M. intracellulare*, *M. marinum*, *M. scrofulaceum*, *M. ulcerans*, and *M. xenopi*. Each scale bar indicates 1 μm.

Rapid and slow grower species belonging to 5 genera in the family *Mycobacteriaceae* were examined cryo-TEM. 1,816 cells of 31 species (34 strains) were examined (Supplementary Figures S4–S8). Representative cryo-TEM images of rapid grower species belonging to genus *Mycobacteroides* (6 species) and genus *Mycolicibacterium* (6 species, 7 strains) were shown in Figures 1, 2, respectively. In addition, representative cryo-TEM images of slow grower species belonging to genus *Mycolicibacter* (3 species), genus *Mycolicibacillus* (3 species), and genus *Mycobacterium* (13 species, 15 strains) were shown in Figures 3, 4, respectively.

### Measurements of Cell Morphological Parameters and Comparison Between the Genera

**FIGURE 5:**
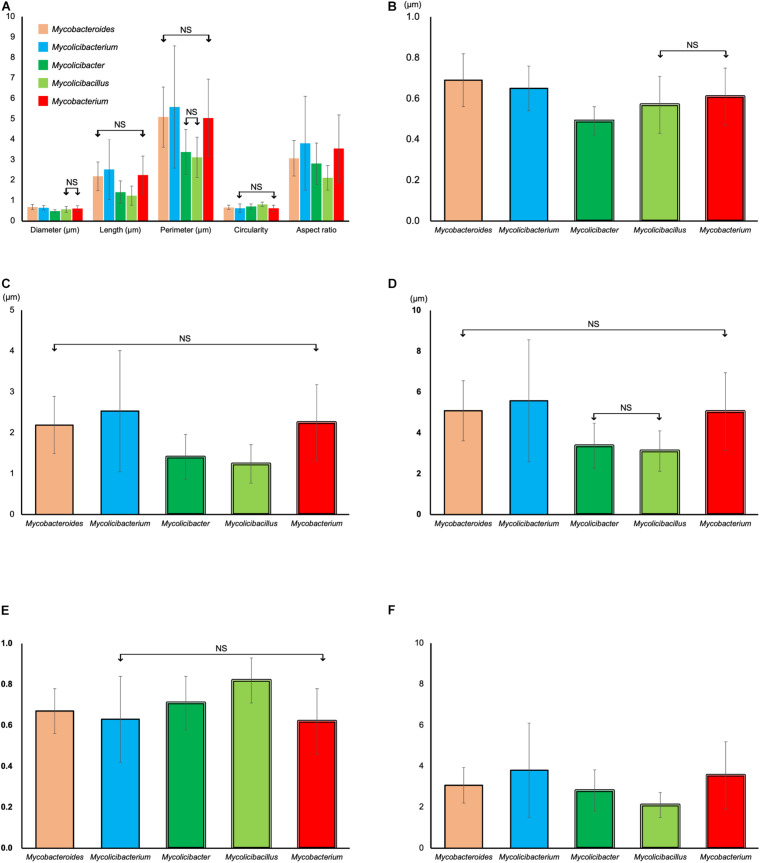
Comparison of the average of the cell diameter, the cell length, the cell perimeter, the cell circularity, and the aspect ratio between genera. **(A)** Overview of the whole comparison. **(B)** Comparison of the cell diameter between five genera. **(C)** Comparison of the cell length between five genera. **(D)** Comparison of the cell perimeter between five genera. **(E)** Comparison of the cell circularity between five genera. **(F)** Comparison of aspect ratio between five genera. Comparisons without significant difference between two genera were indicated as NS. Detail comparison data were shown in Supplementary Tables S1–S4.

Cell diameter, cell length, cell perimeter, circularity, and the aspect ratio of each cell were measured and analyzed with ImageJ/Fiji (Table 4, Figure 5A, and Supplementary Tables S1–S4). In the comparison of the cell diameter, the average value of the whole cells was 0.63 ± 0.14 μm. It was extremely similar, where the averages of the genus ranged from 0.49 ± 0.07 to 0.69 ± 0.13 μm in genus *Mycolicibacter* and genus *Mycobacteroides*, respectively. There are significant differences between five genera (*p* < 0.000009) except for the comparison of the values between slow grower genus *Mycolicibacillus* and genus *Mycobacterium*, 0.57 ± 0.14 and 0.61 ± 0.14 μm, respectively (*p* = 0.15, Figure 5B). Because standard deviations (SD) within every genus were small, small *p*-values were calculated despite small differences between genera (Supplementary Table S1).

**TABLE 4:** Comparison of average values in diameter, length, perimeter, circularity, and aspect ratio of the cells between genera.

	Genus				
	
	Rapid grower		Slow grower		
		
	*Mycobacteroides*	*Mycolicibacterium*	*Mycolicibacter*	*Mycolicibacillus*	*Mycobacterium*
Species (strains)	6	6 (7)	3	3	13 (15)
Number of examined cells	512	297	125	68	814
Diameter (μm)	0.63 ± 0.14*				
Average	0.69	0.65	0.49	0.57	0.60
SD	0.13	0.11	0.07	0.14	0.13
Minimum	0.43	0.45	0.24	0.38	0.33
Maximum	1.15	1.00	0.70	0.91	1.03
Length (μm)	2.18 ± 1.01*				
Average	2.19	2.51	1.41	1.24	2.25
SD	0.70	1.46	0.55	0.47	0.93
Shortest	0.78	0.94	0.53	0.60	0.77
Longest	6.31	8.58	3.74	2.83	6.98
L/S ratio	8.14	9.10	7.00	4.71	9.02
Perimeter (μm)	4.96 ± 2.04*				
Average	5.09	5.52	3.38	3.12	5.05
SD	1.47	2.88	1.10	0.99	1.90
Minimum	2.20	2.62	1.75	1.86	2.27
Maximum	13.8	17.8	7.99	6.49	13.6
Circularity	0.65 ± 0.16*				
Average	0.67	0.63	0.71	0.82	0.62
SD	0.11	0.21	0.13	0.11	0.16
Minimum	0.40	0.18	0.35	0.56	0.17
Maximum	0.96	0.96	0.98	0.97	0.97
Aspect ratio	3.36 ± 1.59*				
Average	3.07	3.80	2.81	2.11	3.56
SD	0.87	2.30	1.01	0.60	1.64
Minimum	1.23	1.30	0.98	1.14	1.17
Maximum	6.22	14.4	7.21	3.79	15.8

*Total average and standard deviation.

In the comparison of the cell length, the average value of the whole cells was 2.18 ± 1.01 μm, and the average values of each genus varied from genus to genus, where the values ranged from 1.24 ± 0.47 to 2.53 ± 1.48 μm in genus *Mycolicibacillus* and genus *Mycolicibacterium*, respectively. There are significant differences between five genera (*p* < 0.03) except for the comparison of the values between rapid grower genus *Mycobacteroides* and slow grower genus *Mycobacterium*, 2.19 ± 0.70 and 2.25 ± 0.93 μm (*p* > 0.24), respectively (Table 4, Figure 5C, and Supplementary Table S1). Cell length and diameter distributions of single-cell were shown in Supplementary Figure S1.

Cryo-TEM examinations also revealed the large deviations in single-cell length both in the species and in the genus. For example, in the analysis of cell length variation in the examined species, the shortest and the longest cell lengths were extracted, and the ratio of the longest versus the shortest (L/S) was calculated. Then, half of the examined species showed L/S value of less than 3.0; the others showed more than 3.0 (Supplementary Figure S2). This means that in half of the examined species the cell divisions occurred asymmetrically. Especially, *Mycobacteroides immunogenum*, *Mycolicibacterium fortuitum* subsp. *fortuitu*m (ATCC 6841), *Mycobacterium microti*, *Mycobacterium avium*, and *Mycobacterium celatum* (ATCC 51130), which showed L/S more than 6 (Supplementary Figure S2). This suggests that highly asymmetrical cell division may have occurred in these species. Some examples of the asymmetric cell division were shown in the cryo-TEM images of MSG in Figure 2 and *M. celatum* (ATCC 51130 and ATCC 51131) in Figure 4 and Supplementary Figures S4–S8.

In the comparison of the cell perimeter, the average value of the whole cells was 4.96 ± 2.04 μm, and the average values of each genus varied from genus to genus, where the values ranged from 3.12 ± 0.99 to 5.58 ± 2.99 μm in genus *Mycolicibacillus* and genus *Mycolicibacterium*, respectively. There are significant differences between five genera (*p* < 0.002) except for the comparison of the values between rapid grower genus *Mycobacteroides* and slow grower genus *Mycobacterium*, 5.09 ± 1.47 and 5.05 ± 1.90 μm (*p* > 0.68), respectively, and between slow grower genus *Mycolicibacter* and genus *Mycolicibacillus*, 3.38 ± 1.10 and 3.12 ± 0.99 μm (*p* > 0.11), respectively (Table 4, Figure 5D, and Supplementary Table S2).

In the comparison of the cell circularity, which was calculated in each cell with the formula, 4π × area/perimeter^2^, measured with Fiji, the average value of the whole cells was 0.65 ± 0.16, and the average values of each genus varied from genus to genus with small differences, where the values ranged from 0.62 ± 0.16 to 0.82 ± 0.11 in genus *Mycobacterium* and genus *Mycolicibacillus*, respectively. There are significant differences between five genera (*p* < 0.00017) except for the comparison of the values between rapid grower genus *Mycolicibacterium* and slow grower genus *Mycobacterium*, 0.63 ± 0.21 and 0.62 ± 0.16 (*p* > 0.73), respectively (Table 4, Figure 5E, and Supplementary Table S3).

In the comparison of the cell aspect ratio, the average value of the whole cells was 3.36 ± 1.59, and the average values of each genus varied from genus to genus, where the values ranged from 2.11 ± 0.60 to 3.80 ± 2.30 in genus *Mycolicibacillus* and genus *Mycolicibacterium*, respectively. There are significant differences between five genera (*p* < 0.044) without any exception (Table 4, Figure 5F, and Supplementary Table S4).

## Discussion

1,816 cells belonging to 5 genera in family *Mycobacteriaceae* were examined in ice-embedded whole-mount cryo-TEM, and the fundamental cell properties were obtained through Fiji/ImageJ software. In a comparison between newly proposed genera (Gupta et al., 2018), there were a substantial number of significant differences in cell diameter, cell length, cell perimeter, circularity, and aspect ratio. However, the deviation in cell diameter was small despite significant differences. The total average was 0.63 μm with an SD of 0.14 μm, which is less than 25% of the average. Furthermore, the average in three genera *Mycobacteroides*, *Mycolicibacterium*, and *Mycobacterium* ranged from 0.61 to 0.69 μm, and those of two genera *Mycolicibacter* and *Mycolicibacillus* ranged from 0.49 to 0.57 μm. These data suggest that the cell diameters of the species in the family *Mycobacteriacea* maybe strictly regulated with the conserved genetical manner by the actin homolog MreB (Table 4; Melzer et al., 2018), as the cell diameters of model bacteria such as *E. coli* and *B. subtilis* are regulated by the cytoskeletal protein MreB (Dion et al., 2019; Kurita et al., 2019; Patel et al., 2020).

In contrast, there are significant differences in the average cell length between five genera (Table 4). However, the average values of the single species in each genus were not even, but heterogeneous. The cell length varied from not only genus to genus on average, but also species to species within the same genus, and also from cell to cell within the same species (Supplementary Figures S1, S2).

There are two groups of species based on the longest cell length versus the shortest cell length (L/S) ratio, that is, L/S ratio < 3 species (group 1) and L/S ratio > 3 species (group 2). As shown in Supplementary Figures S1, S2, in genus *Mycobacteroides*, group 1 vs. group 2 ratio was 3: 3, in genus *Mycolicibacterium*, that was 4: 3, in genus *Mycolicibacter*, that was 0: 3, in *Mycolicibacillus*, that was 2: 1, and in genus *Mycobacterium*, that was 7: 8. There are some reports about asymmetric cell division in species belonging to genera in the family *Mycobacteriaceae* (Aldridge et al., 2012; Joyce et al., 2012; Santi et al., 2013; Singh et al., 2013; Wakamoto et al., 2013), such as MSG and MTB. Based on this study, the cells of group 2 species with high L/S ratio may divide asymmetrically, such as *Mycobacteroides immunogenum*, *Mycolicibacterium fortuitum*, species belonging to genus *Mycolicibacter*, *Mycobacterium microti*, *Mycobacterium avium*, *Mycobacterium celatum*, *Mycobacterium intracellulare*, *Mycobacterium marinum*, *Mycobacterium scrofulaceum*, and *Mycobacterium xenopi* (Supplementary Figures S1, S2). On the other hand, our data suggest that the cells of group 1 species with a low L/S ratio may divide rather symmetrically. It is known that some bacteria use a negative regulation of FtsZ assembly, recognized as nucleoid occlusion. However, because it has been shown that the FtsZ of MSG (group 1) can complement the deletion of MTB (group 2), the asymmetric cell divisions are not attributed to the difference of FtsZ in genotype or phenotype between group 1 and group 2 (Hett and Rubin, 2008; Eswara and Ramamurthi, 2017).

It is noteworthy that in genus *Mycobacterium*, a member of MTB complex, *Mycobacterium microti* showed significantly longer cell length than MTB (both H37Rv and CDC1551), *M. africanum*, and *M. bovis* (*p* < 0.004). There were no significant differences in average cell length between MTB H37Rv and CDC1551 (*p* > 0.6), and between MTB (H37Rv and CDC1551) and *M. africanum* (*p* > 0.3). In contrast, there were significant differences between MTB (H37Rv and CDC1551) and *M. bovis* (*p* < 0.02), and between *M. africanum* and *M. bovis* (*p* < 0.00000003) because average cell length in *M. bovis* was the shortest.

Although members of MTB complex have highly conserved genomes, *M. microti* genome lacks RD1 and RD3–RD10, and MiD1, RD1b, MiD2, and MiD3 (Brodin et al., 2002; Frota et al., 2004; Mostowy et al., 2004). However, genomic deletions of RD1 and RD3–RD10 were also identified in *M. bovis* BCG. In contrast, MiD deletions are specific for *M. microti*, and, MiD3 deletion was universally present to any strains examined (Brodin et al., 2002). Therefore, specific cell morphology in *M. microti* may be attributed to MiD3 deletion, since MiD3 deletion has been identified in *M. pinnipedii*, the cell morphology of *M. pinnipedii* may validate this conjecture (Supplementary Figure S3).

In the comparison of the cell perimeter and aspect ratio, the differences between the five genera were similar to those of the cell length because the cell perimeters and the aspect ratios were in direct proportion to the cell length. On the other hand, in the comparison of the cell circularity, the differences were inversely correlated with the cell length, where genus *Mycolicibacillus*, which had the shortest average cell length, showed the highest circularity of the five genera.

In this study, the fundamental cell morphologies of the species belonging to the family *Mycobacteriaceae* were analyzed based on whole-mount ice-embedded cryo-TEM examinations. There have been studies that analyzed cell morphology. However, most of these studies only analyzed cell lengths because it is difficult to precisely analyze cell morphology besides the cell length due to the resolution power of light microscopical examination and to cell shrinkage during sample preparation in the ultrathin section of TEM examination and conventional SEM examination (Yamada et al., 2015, 2017). Data presented in this study are the first comprehensive quantitative information about the cell morphology belonging to the family *Mycobacteriaceae*.

Although some studies dispute the novel classification of the family *Mycobacteriaceae* (Nouioui et al., 2018; Tortoli et al., 2019), others use basonyms (Brzeszcz and Kaszycki, 2018; Chevtchouk Jurno et al., 2019), or another study interpreted five novel genera as sub-genera (Bachmann et al., 2019; Matsumoto et al., 2019), the literature supported and used the novel classification (Ito et al., 2018; Yamada et al., 2018; Cereija et al., 2019; Del Barrio-Duque et al., 2019; Kim et al., 2019; Sánchez et al., 2019; Tiago et al., 2019; Zhou et al., 2019; Chin et al., 2020; Salam et al., 2020; Tan et al., 2020). This work reflects comparative morphological studies by Gupta et al. (2018). Although it does not cover all species belonging to four genera *Mycobacteroides*, *Mycolicibacterium*, *Mycolicibacter*, and *Mycobacterium*, it is one of the most comprehensive morphological analysis to date and includes all the species belonging to the genus *Mycolicibacillus*. In addition, it is crucial that our data, which showed significant differences in the cell morphology parameters between five genera, strongly support the novel classification of the family *Mycobacteriaceae* proposed by Gupta et al. (2018) in terms of the cell morphology. Because genus *Mycolicibacterium* and genus *Mycobacterium* contain many species with varied morphological properties, these two genera may be further subdivided in future studies.

This study used samples with a single culture period for each species. Because only a small number of cells were examined in some species due to sample preparation immaturity, further investigations are required to provide additional cell morphological data based on the different culture periods and examination with a larger number of cells, especially in two genera *Mycolicibacterium* and *Mycobacterium*, both of which contain more than 50 species. Besides, the longer cells may have less chance to be analyzed than the shorter cells because the former may have more chances to localize with overlapping other cells than shorter cells and cannot be accurately traced to the outline. Therefore, the average length, perimeter, and aspect ratio of each species may increase, and the average circularity may decrease according to future investigations. Finally, as we have proposed in our previous report, which undertook a structome analysis in MSG (Yamada et al., 2018), MSG should be interpreted as a completely distinct species from MTB. *M. bovis* BCG strains or MTB auxotroph strains are more appropriate to use as surrogate species or strains, which can be manipulated in the biosafety level 2 facility (Vilcheze et al., 2018).

## Data Availability Statement

All datasets generated for this study are included in the article/Supplementary Material.

## Author Contributions

HY and SMi conceived the project. HY analyzed, interpreted the cryoTEM data, and prepared the figures and manuscript. SMi, KC, and AB collected the strains. HY, KC, AA, AB, NF, and SMa cultured bacterial samples. HY, KM, NM, and YK prepared bacterial samples for cryoTEM examination and manage cryoTEM operation and image capturing. All authors had full access to all the data in the study and approved the final version of the manuscript for submission.

## Conflict of Interest

YK was employed by the company Terabase Inc. The remaining authors declare that the research was conducted in the absence of any commercial or financial relationships that could be construed as a potential conflict of interest.
