# Supplementary material for: Fundamental Cell Morphologies Examined With Cryo-TEM of the Species in the Novel Five Genera Robustly Correlate With New Classification in Family Mycobacteriaceae
Source: Front Microbiol. 2020 Nov 16;11:562395. doi: 10.3389/fmicb.2020.562395 (PMC7701246; doi:10.3389/fmicb.2020.562395)
Supplement: Supplementary file 1 [file Data_Sheet_1.PDF]

Supplementary Material

Fundamental cell morphologies examined with cryo-TEM of the species in the novel five genera robustly correlate with new classification in family *Mycobacteriaceae*

Hiroyuki Yamada[1*], Kinuyo Chikamatsu [1], Akio Aono [1], Kazuyoshi Murata[2], Naoyuki Miyazaki[2,3], Yoko Kayama[4], Apoorva Bhatt[5], Nagatoshi Fujiwara[6], Shinji Maeda[7], Satoshi Mitarai[1,8]

[1] Department of Mycobacterium Reference and Research, the Research Institute of Tuberculosis, Japan Anti-Tuberculosis Association, Tokyo, Japan.

[2] National Institute of Physiological Science, Aichi, Japan.

[3] Tsukuba University, Ibaraki, Japan.

[4] Terabase Inc., Aichi, Japan.

[5] School of Biosciences, University of Birmingham

[6] Department of Food and Nutrition, Faculty of Contemporary Human Life Science, Tezukayama University, Nara, Japan.

[7] Department of Pharmacy, Faculty of Pharmaceutical Science, Hokkaido University of Science, Hokkaido, Japan.

[8] Department of Basic Mycobacteriology, Graduate School of Biomedical Sciences, Nagasaki University, Nagasaki, Japan.

*** Correspondence:**
Dr. Hiroyuki Yamada
hyamada@jata.or.jp

Supplementary Figures

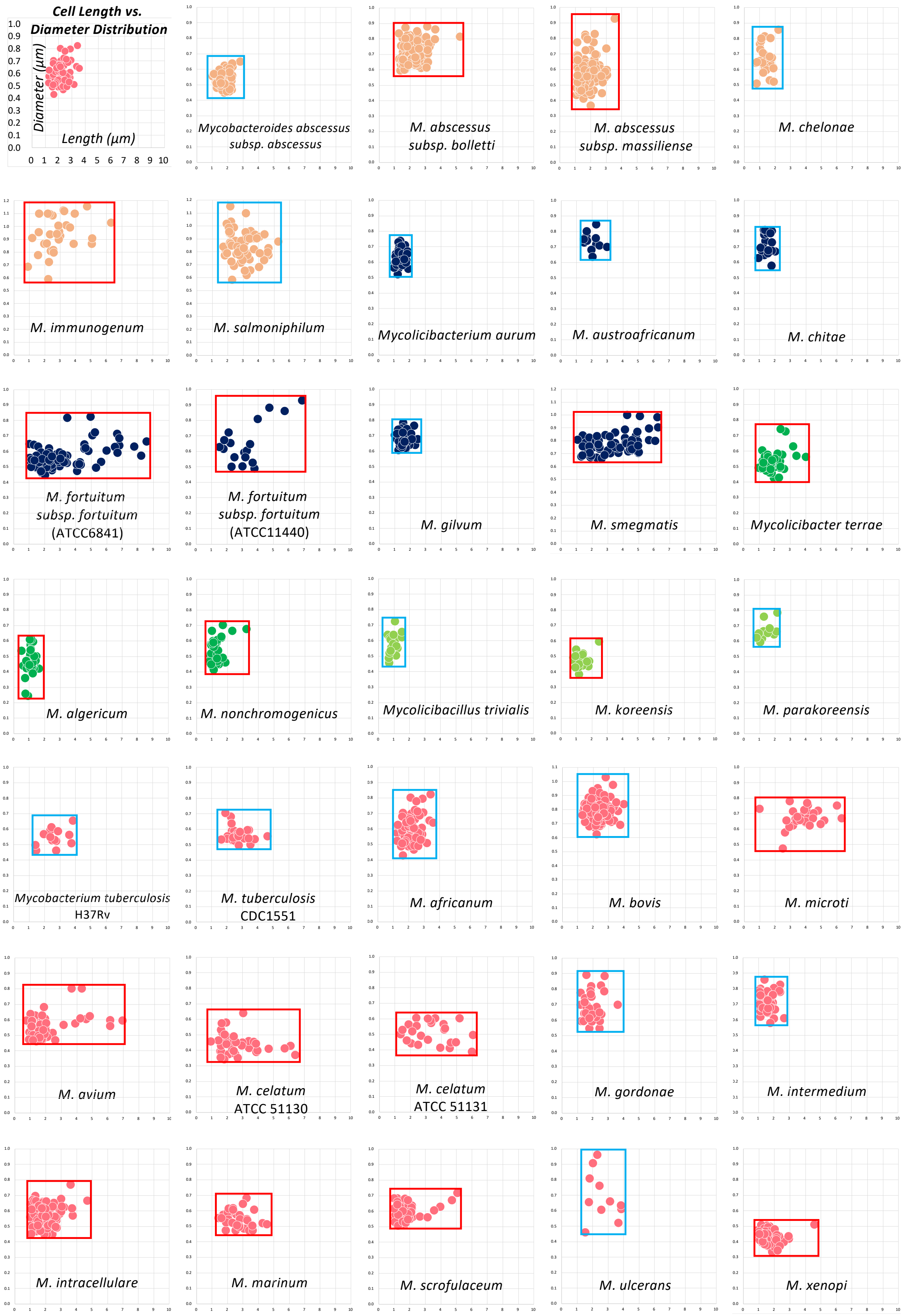

Fig. S1. Length-diameter distributions. Cell length - diameter distributions of each examined cell were plotted. Blue and red rectangles indicate diameter-length distribution with 3.0 or less L/S and L/S more than 3.0, respectively.

Supplementary Figures

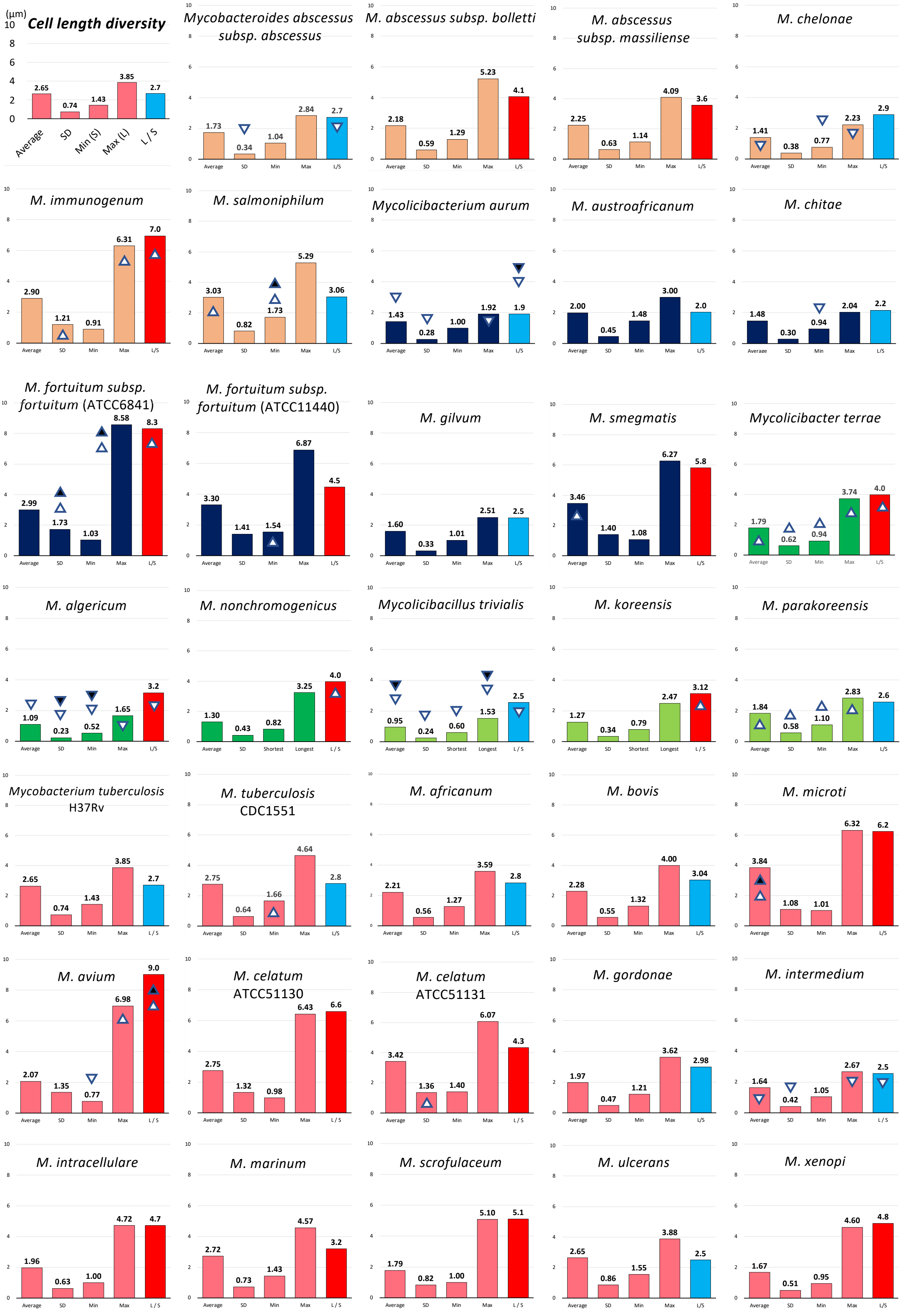

Fig. S2. Cell length diversity. Average, standard deviation, the shortest (min), the longest (max) and Longest / Shortest (L/S) ratio of each species were displayed. ▲ and ▼ indicate the largest and the smallest values among all species, respectively. △ and ▽ indicate the largest and the smallest values within each genus, respectively.

Supplementary Figures

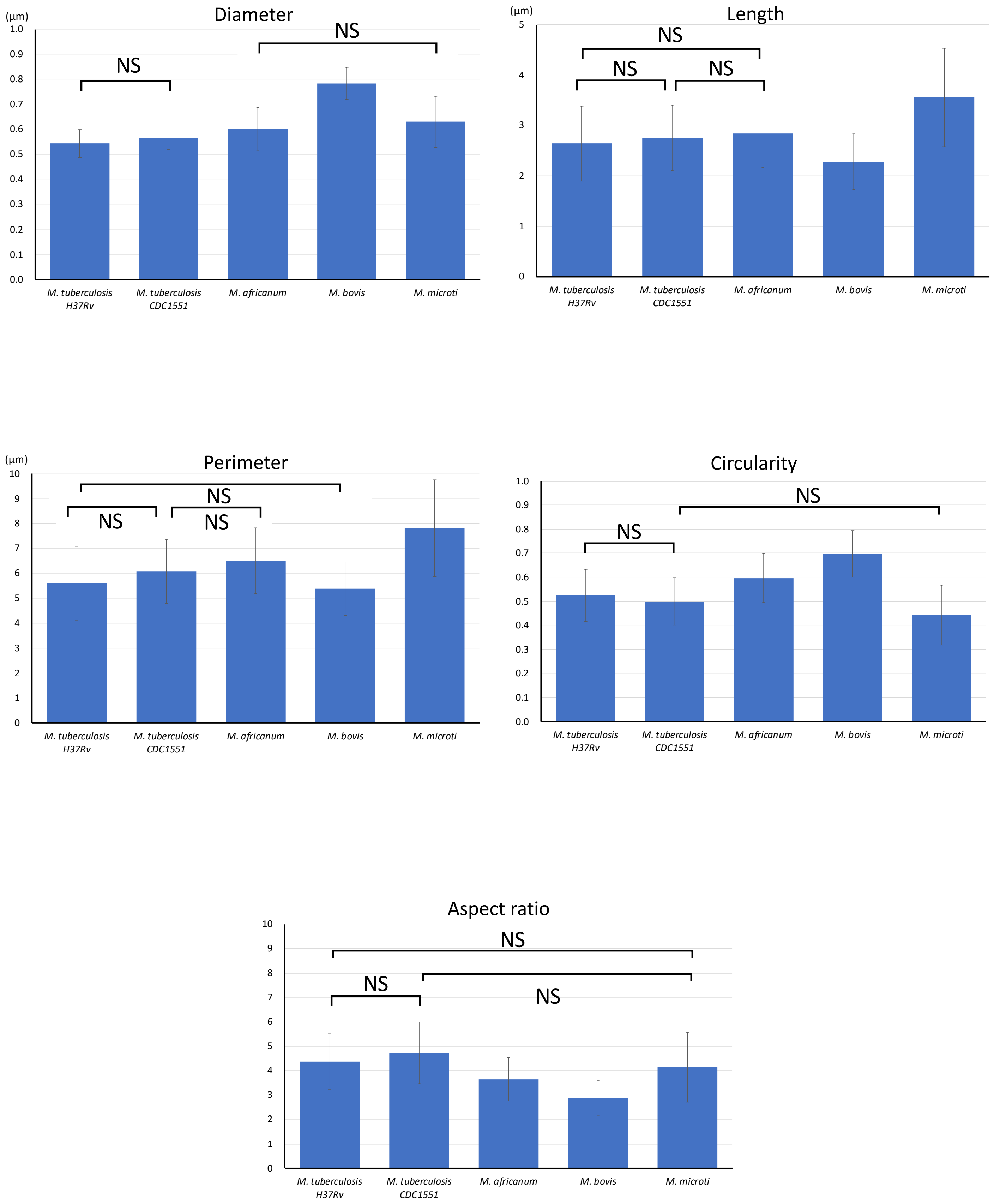

Fig. S3. Comparison of fundamental cell morphology between species in MTB complex. Comparisons without significant difference were indicated as NS.
