# Supplementary material for: Fundamental Cell Morphologies Examined With Cryo-TEM of the Species in the Novel Five Genera Robustly Correlate With New Classification in Family Mycobacteriaceae
Source: Front Microbiol. 2020 Nov 16;11:562395. doi: 10.3389/fmicb.2020.562395 (PMC7701246; doi:10.3389/fmicb.2020.562395)

Supplementary Figure S 4

Genus Mycobacteroides ***Mycobacteroides abscessus subsp. abscessus***

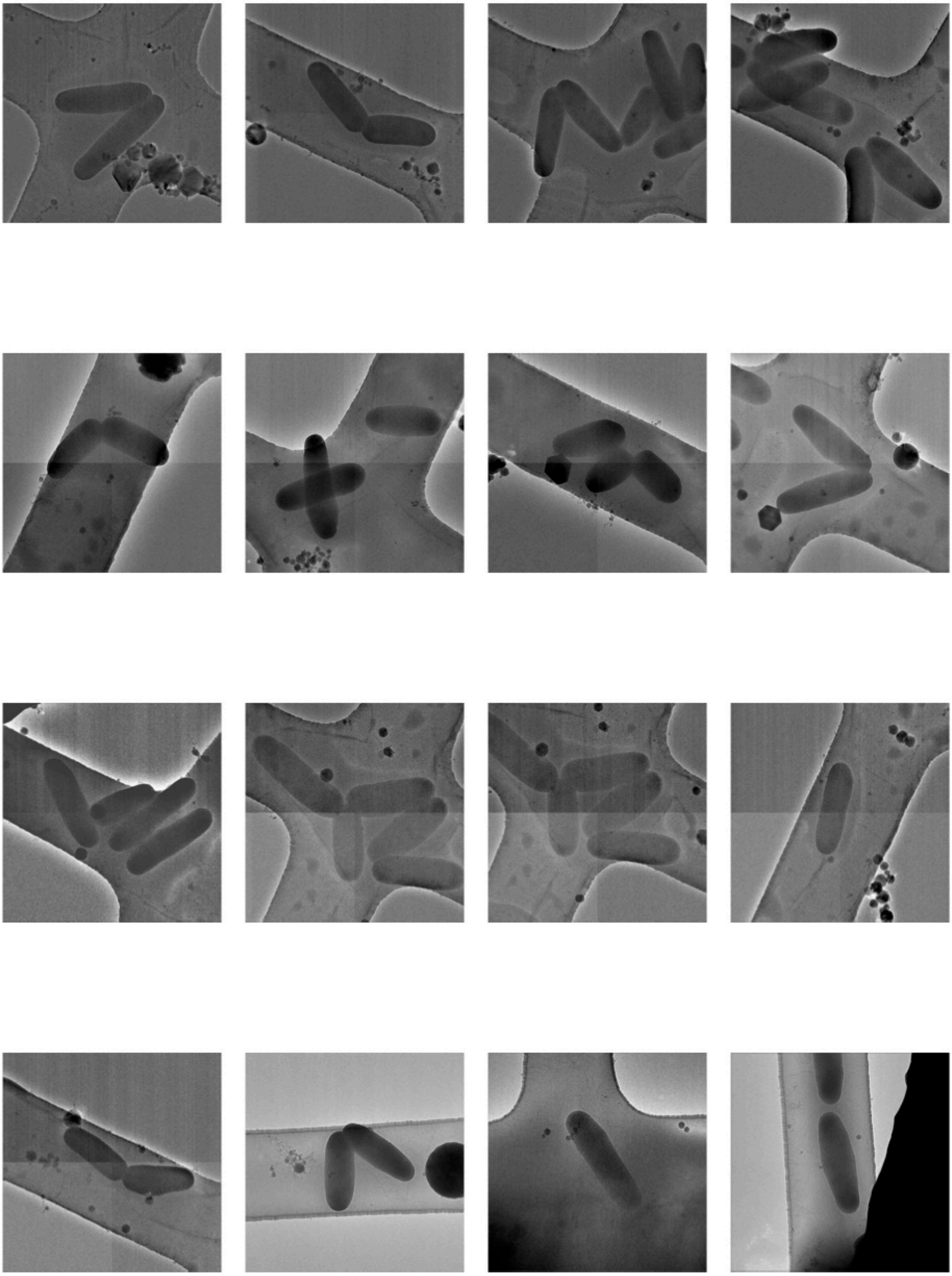

Mycobacteroides abscessus subsp. abscessus

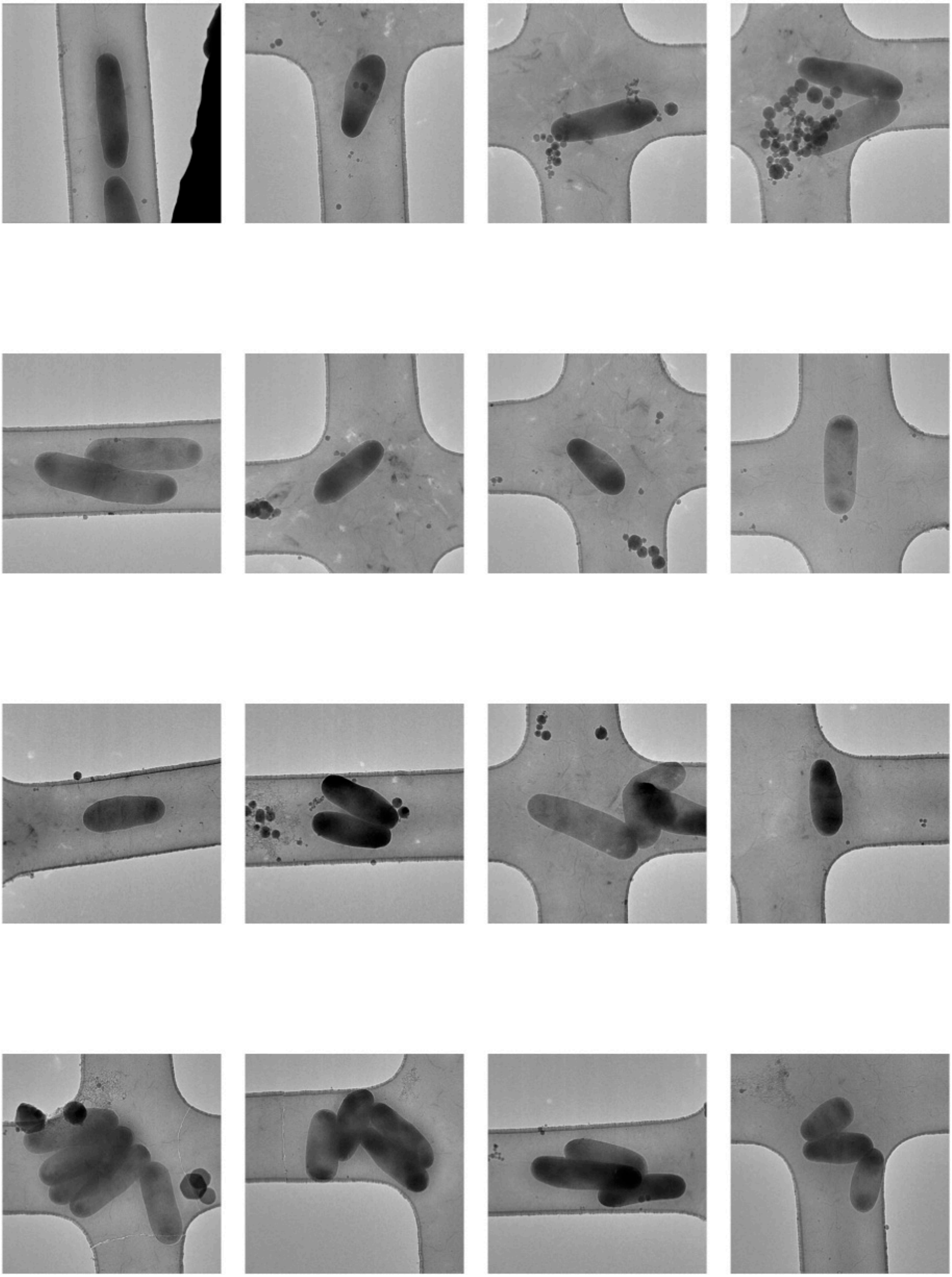

Mycobacteroides abscessus subsp. abscessus

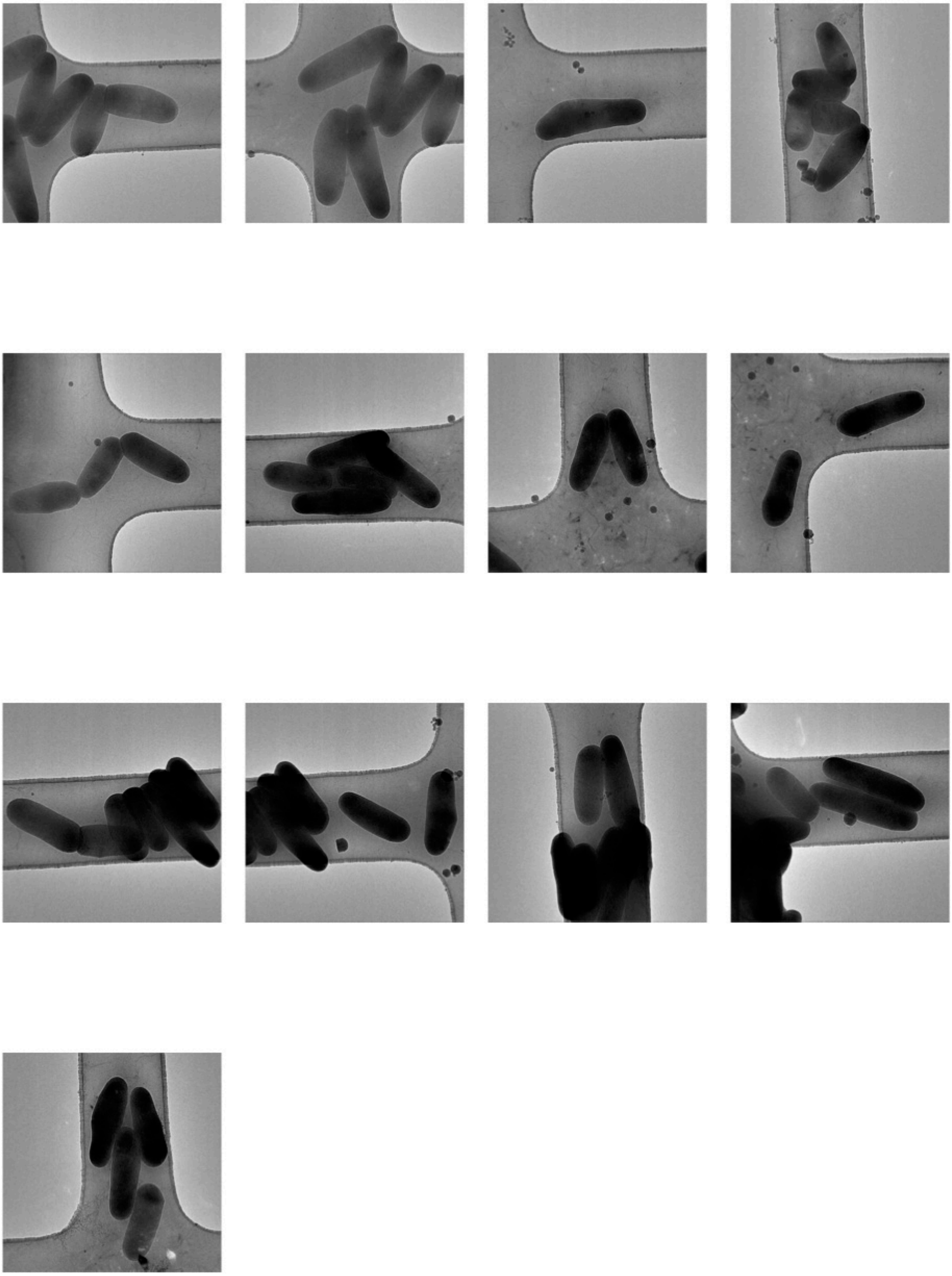

Mycobacteroides abscessus subsp. bolletti

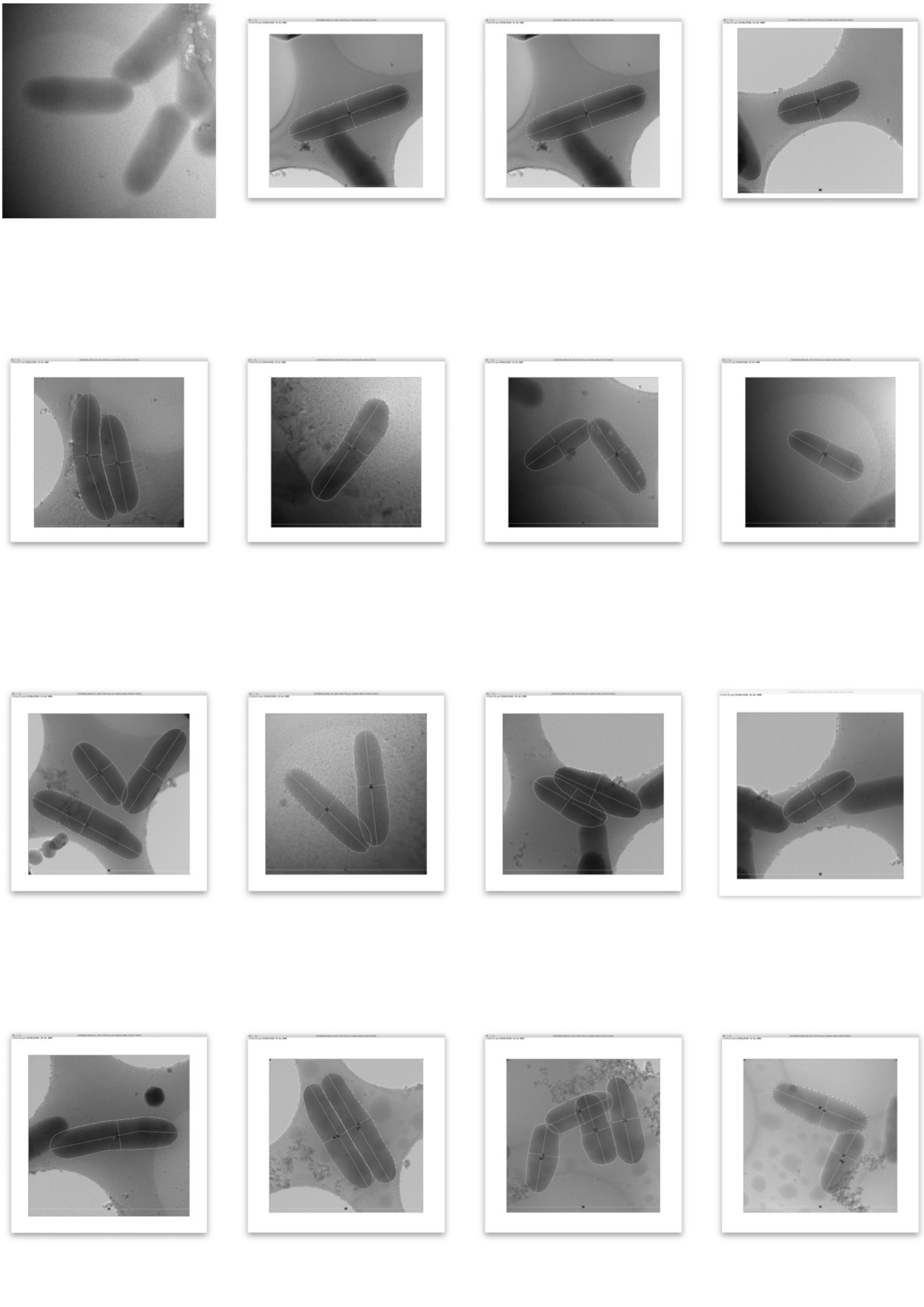

Mycobacteroides abscessus subsp. bolletti

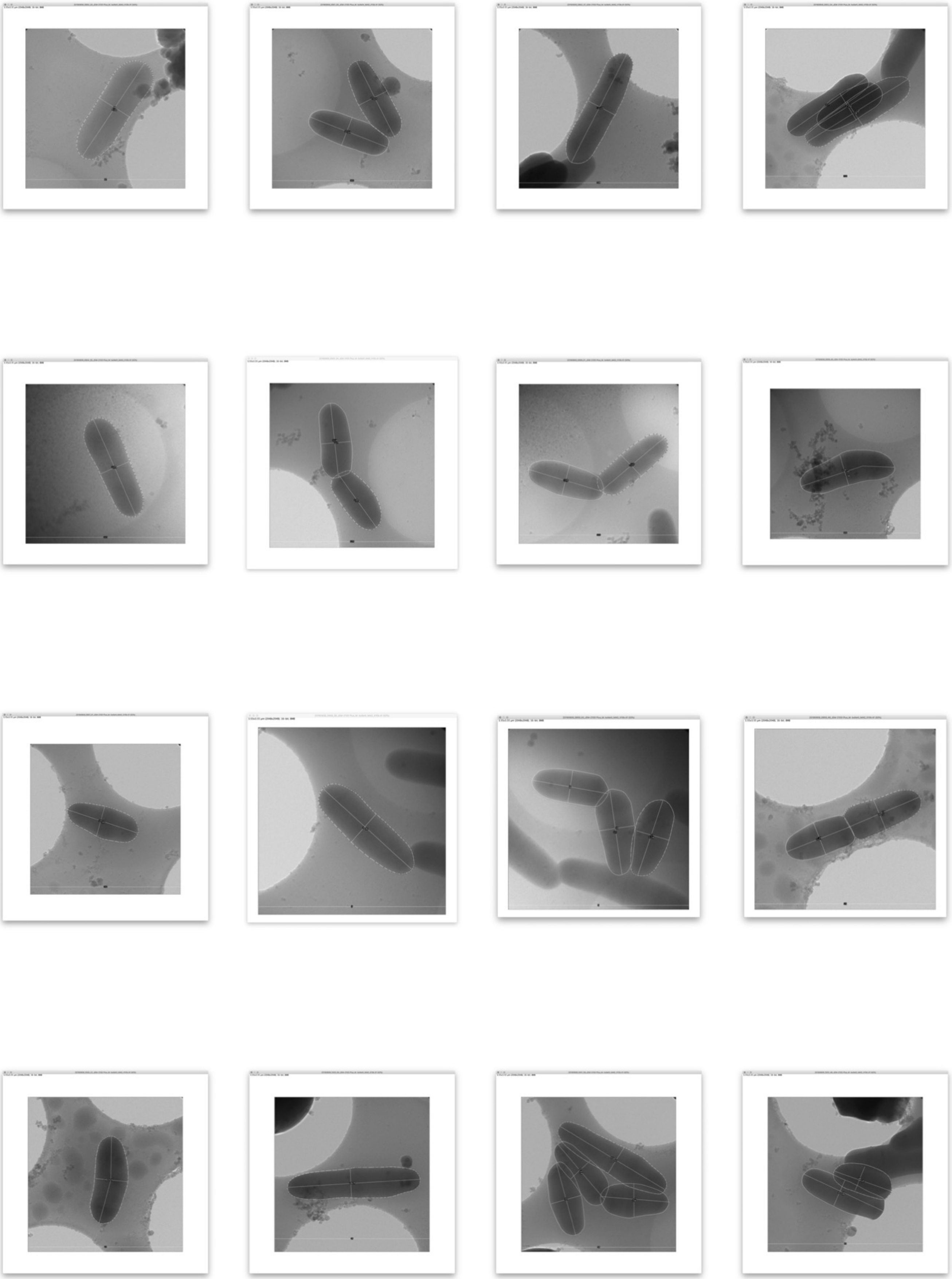

Mycobacteroides abscessus subsp. bolletti

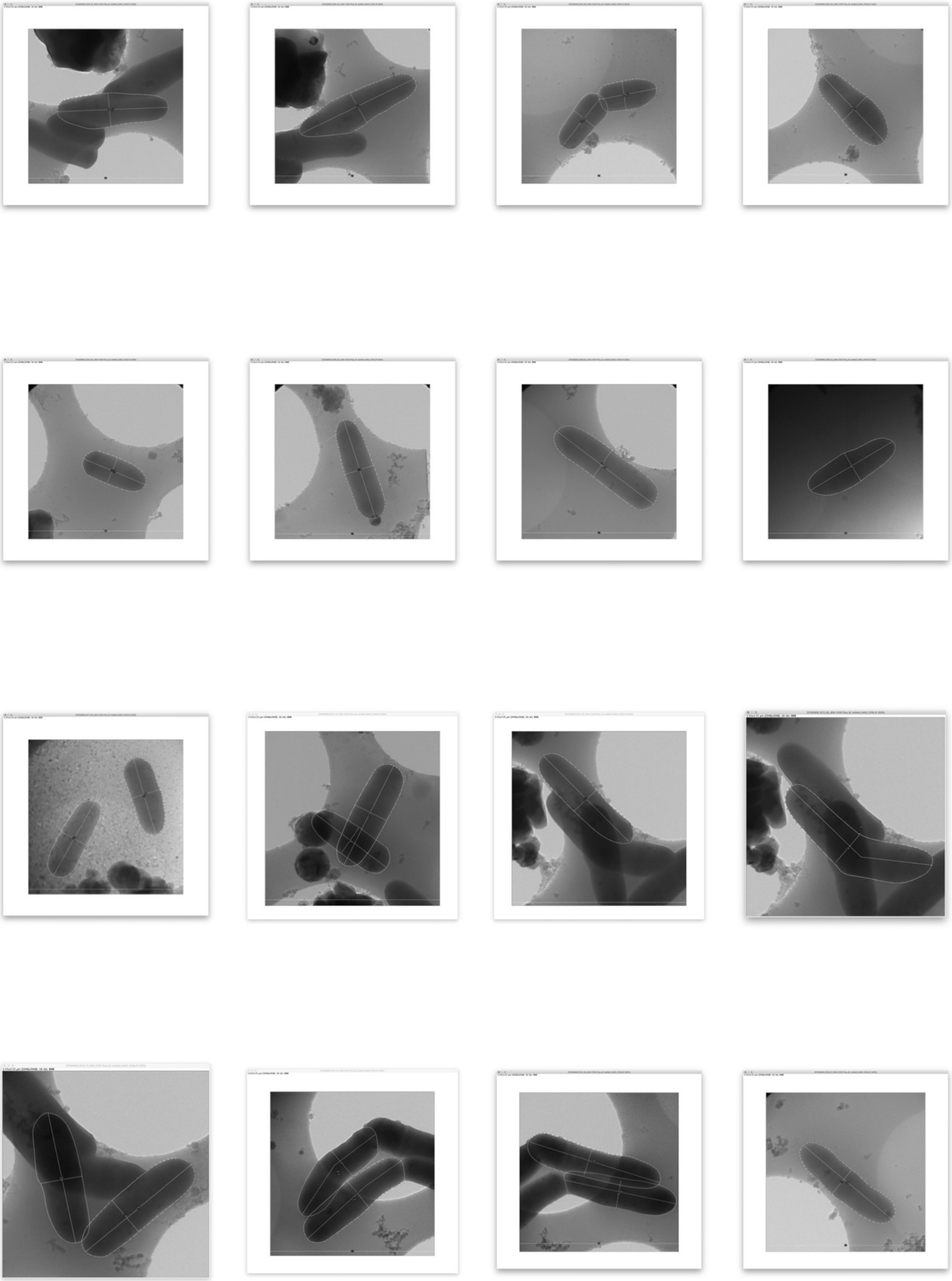

Mycobacteroides abscessus subsp. bolletti

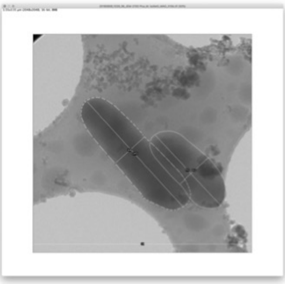
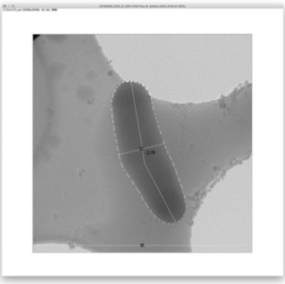
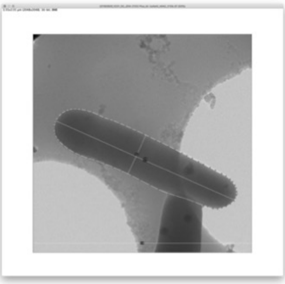
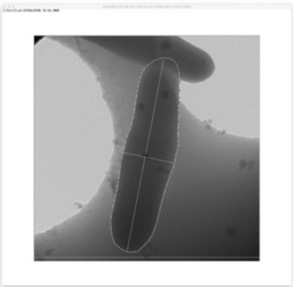

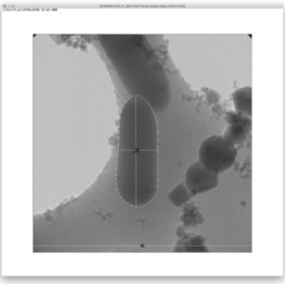
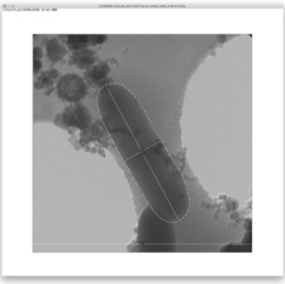
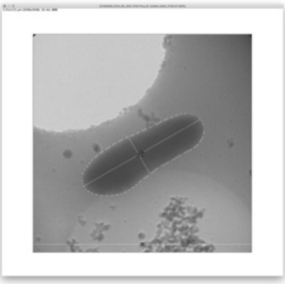
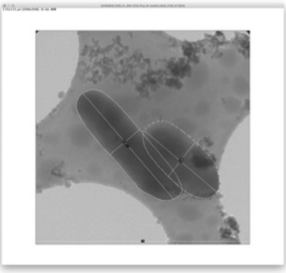

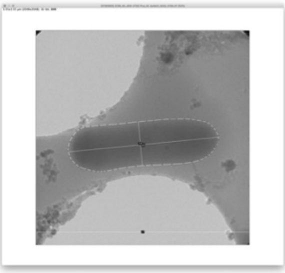
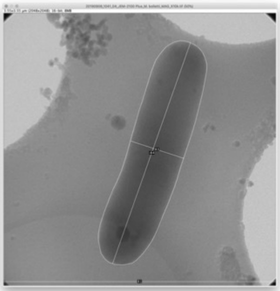
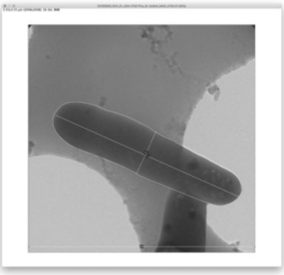
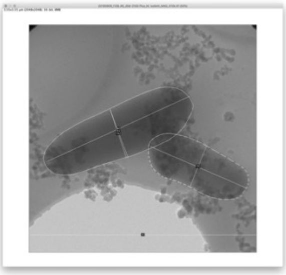

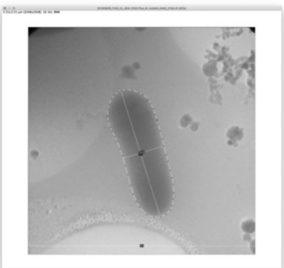
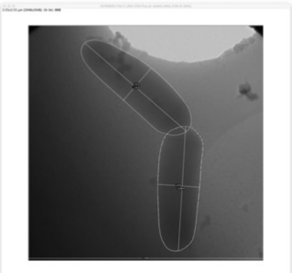
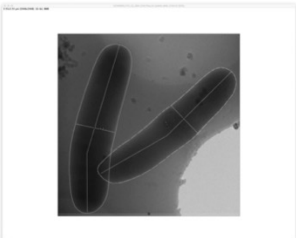
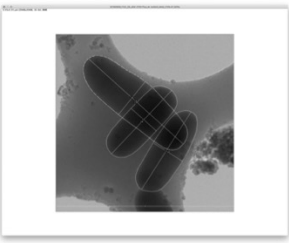

Mycobacteroides abscessus subsp. bolletti

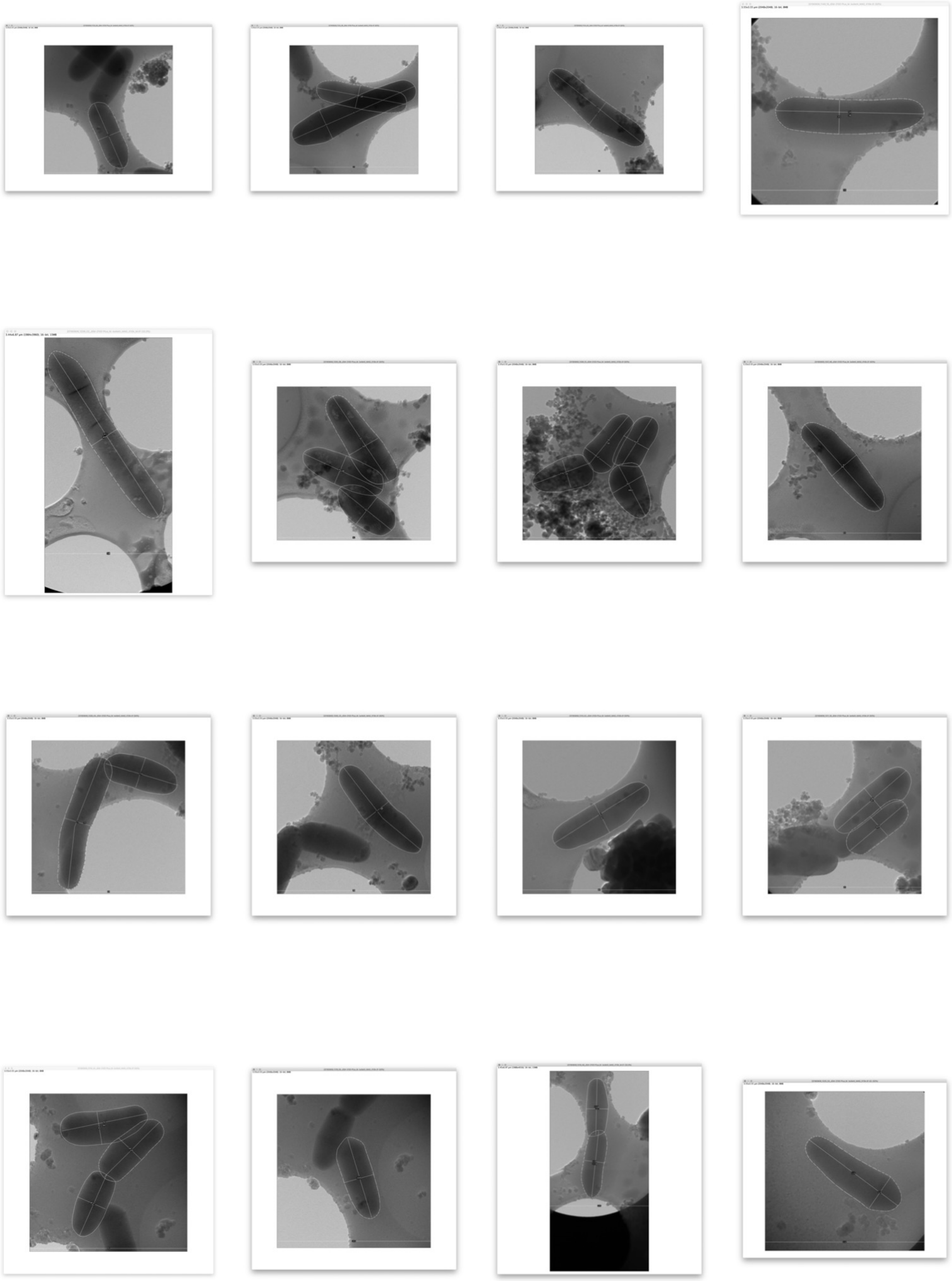

Mycobacteroides abscessus subsp. bolletti

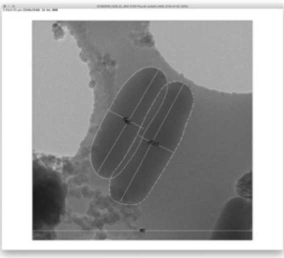
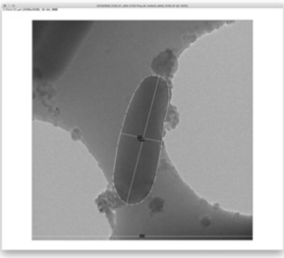
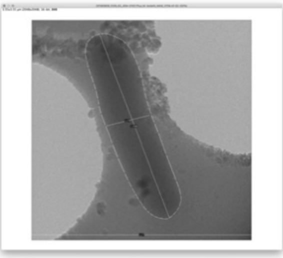
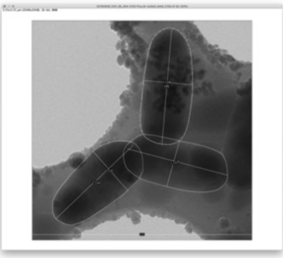

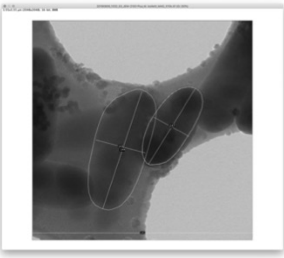
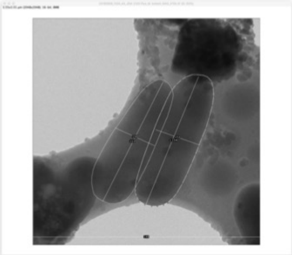
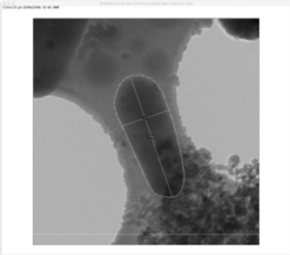
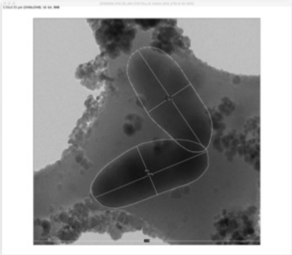

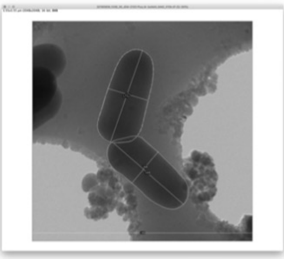
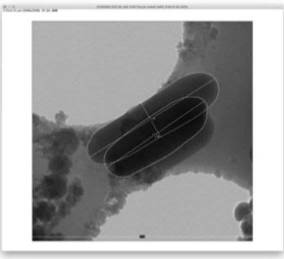
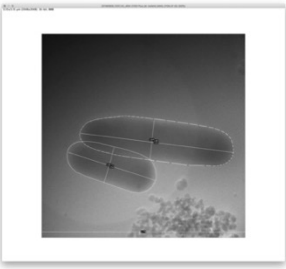
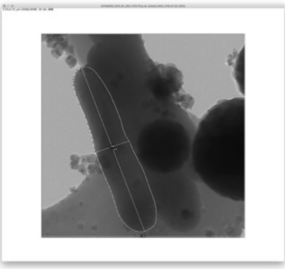

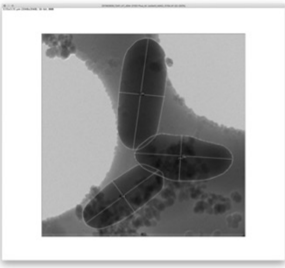
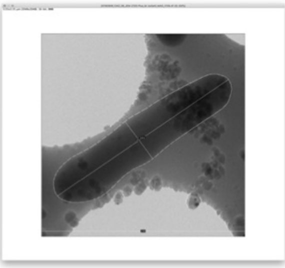
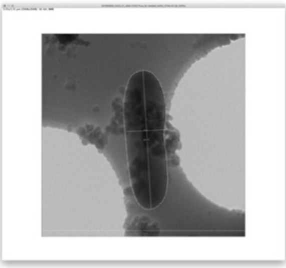
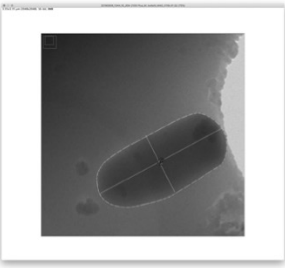

Mycobacteroides abscessus subsp. bolletti

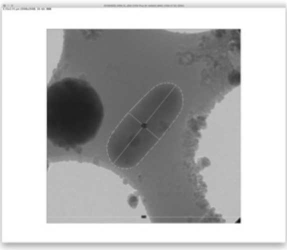

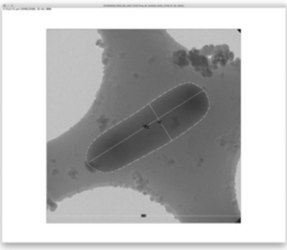

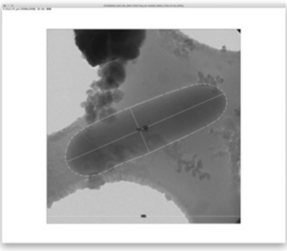

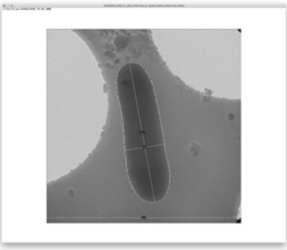

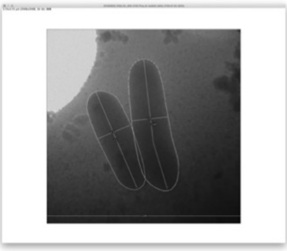

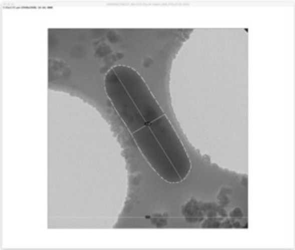

Mycobacteroides abscessus subsp. massiliense

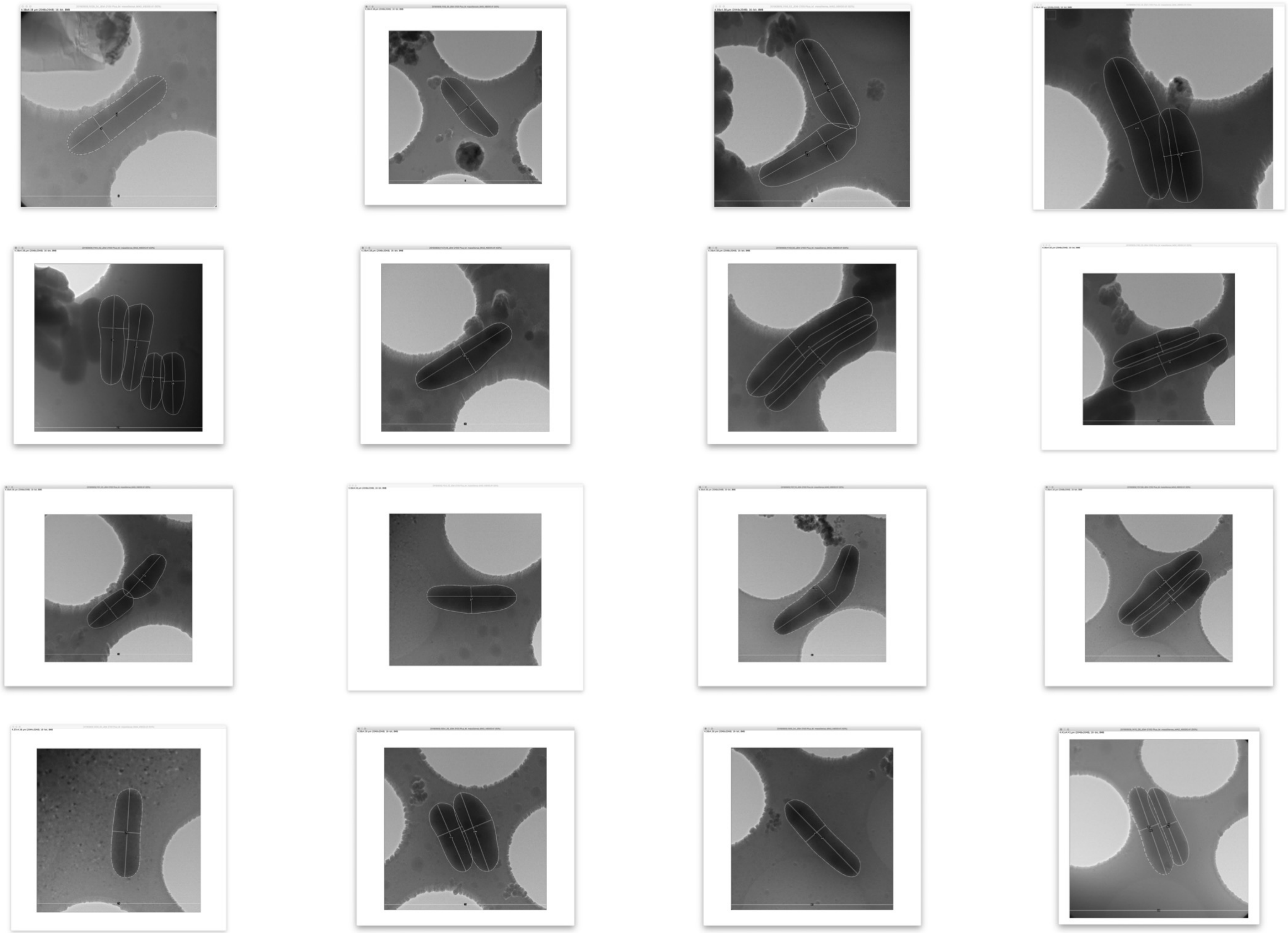

Mycobacteroides abscessus subsp. massiliense

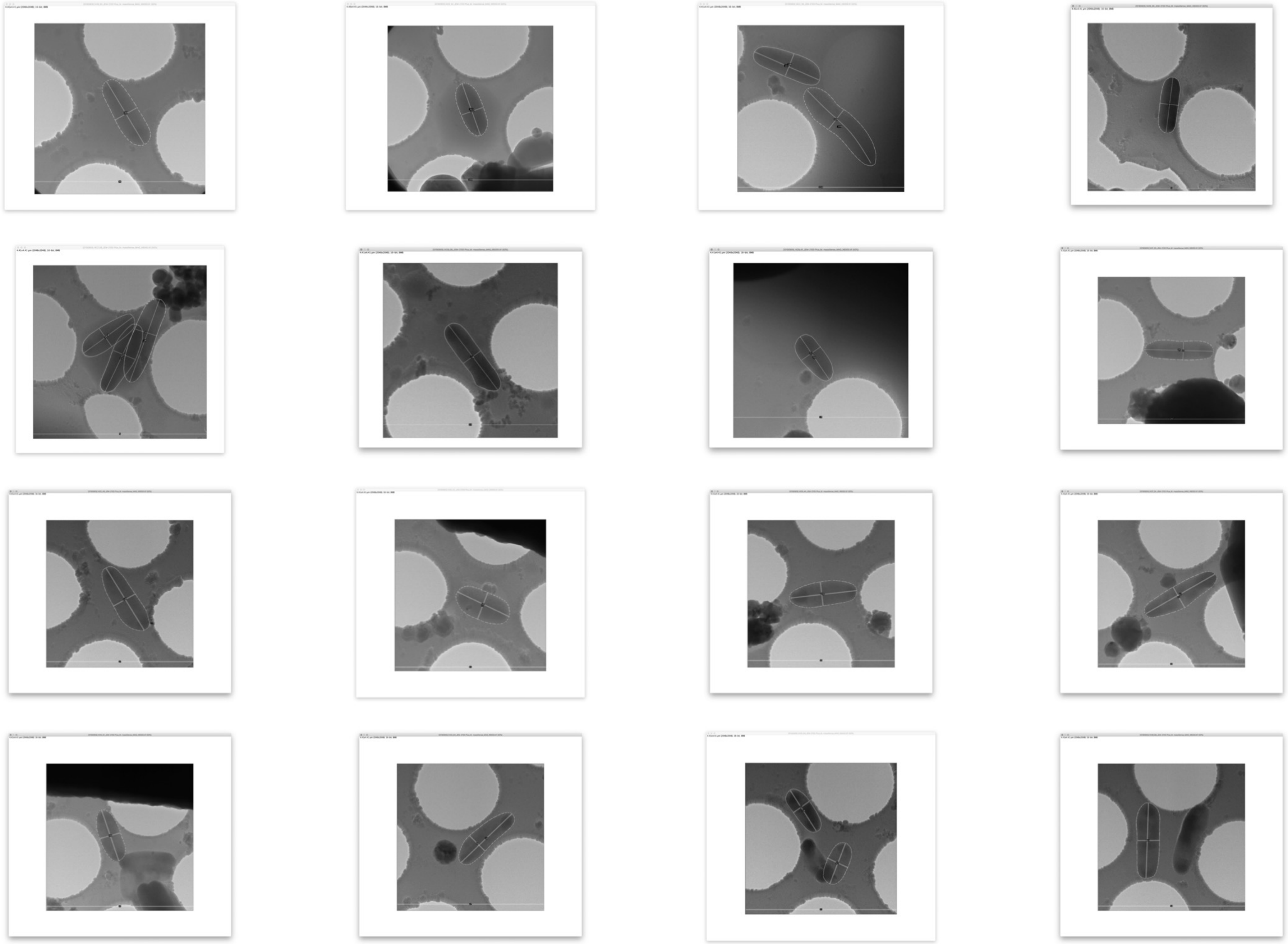

Mycobacteroides abscessus subsp. massiliense

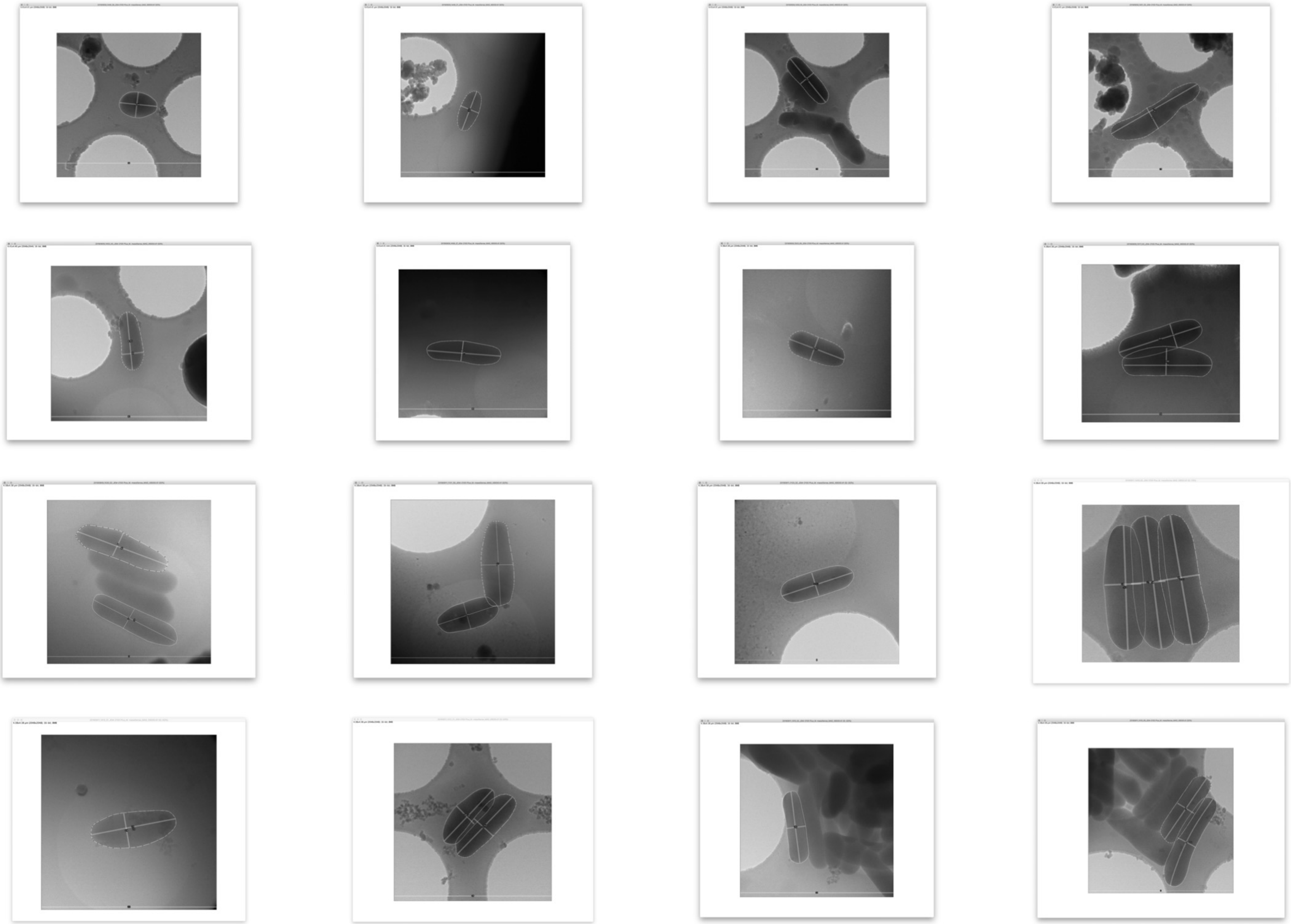

Mycobacteroides abscessus subsp. massiliense

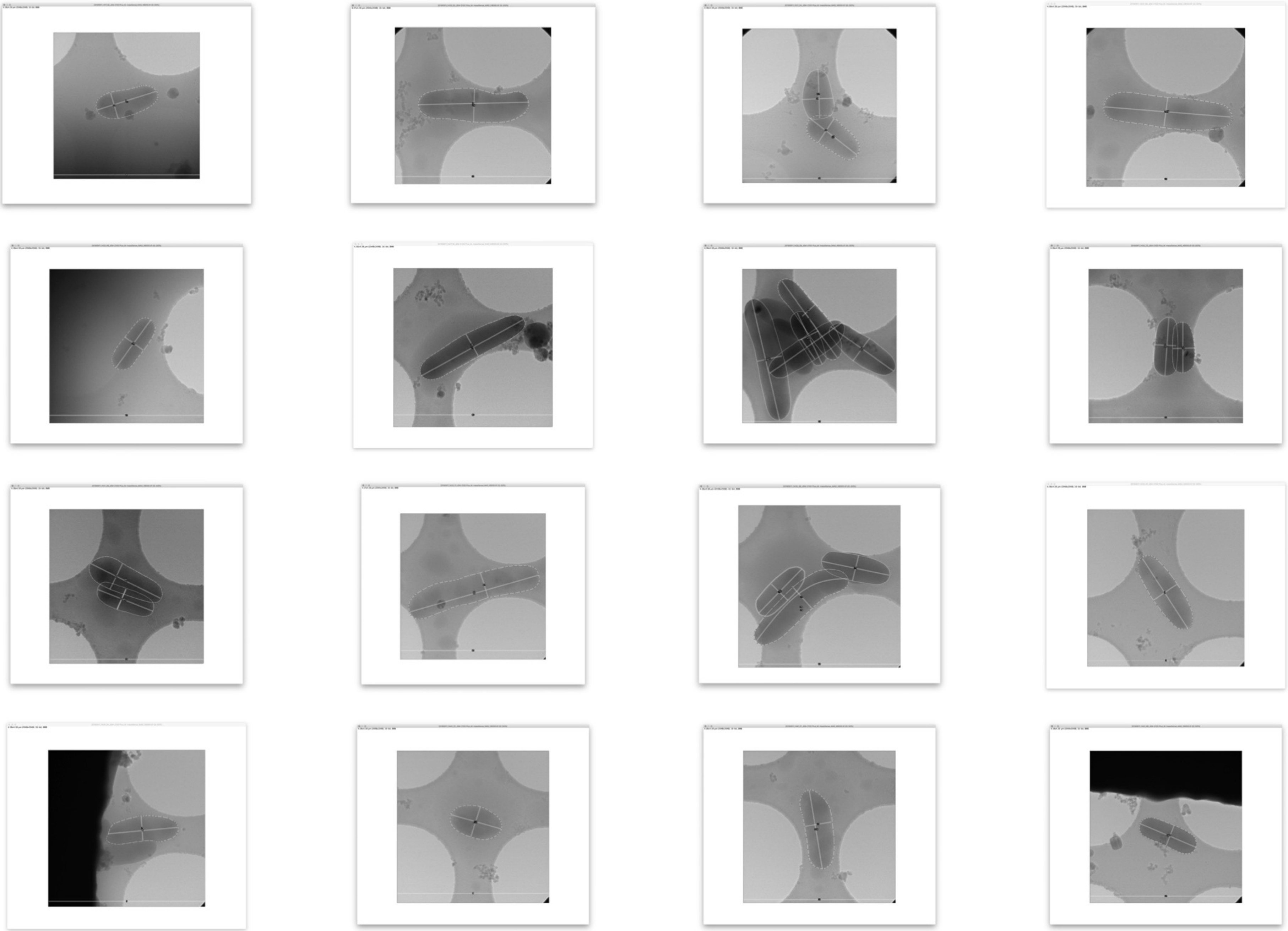

Mycobacteroides abscessus subsp. massiliense

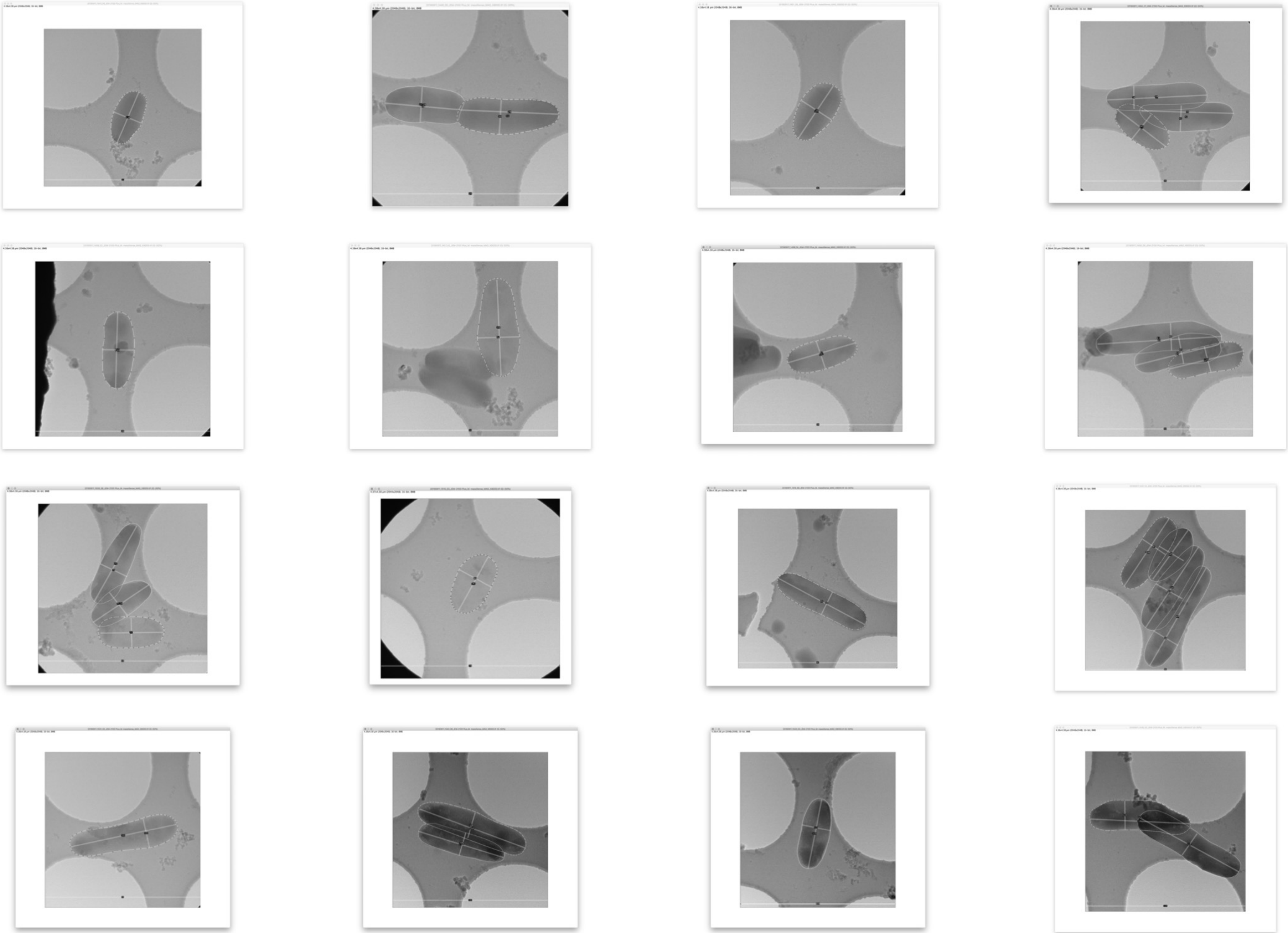

Mycobacteroides abscessus subsp. massiliense

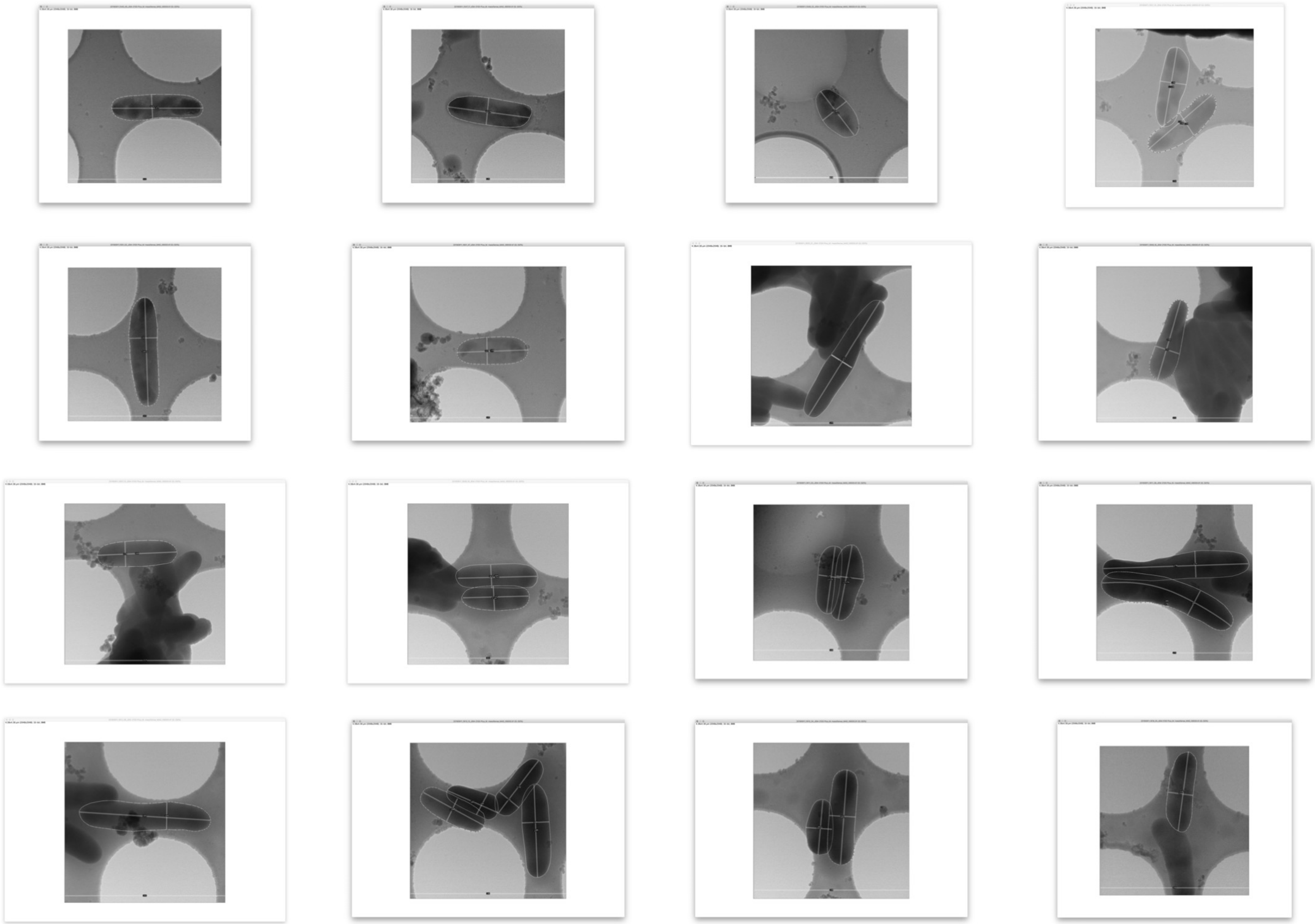

Mycobacteroides abscessus subsp. massiliense

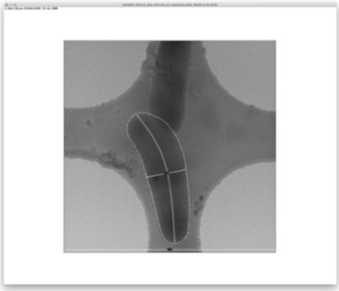

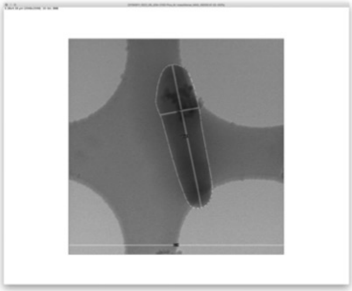

Mycobacteroides chelonae

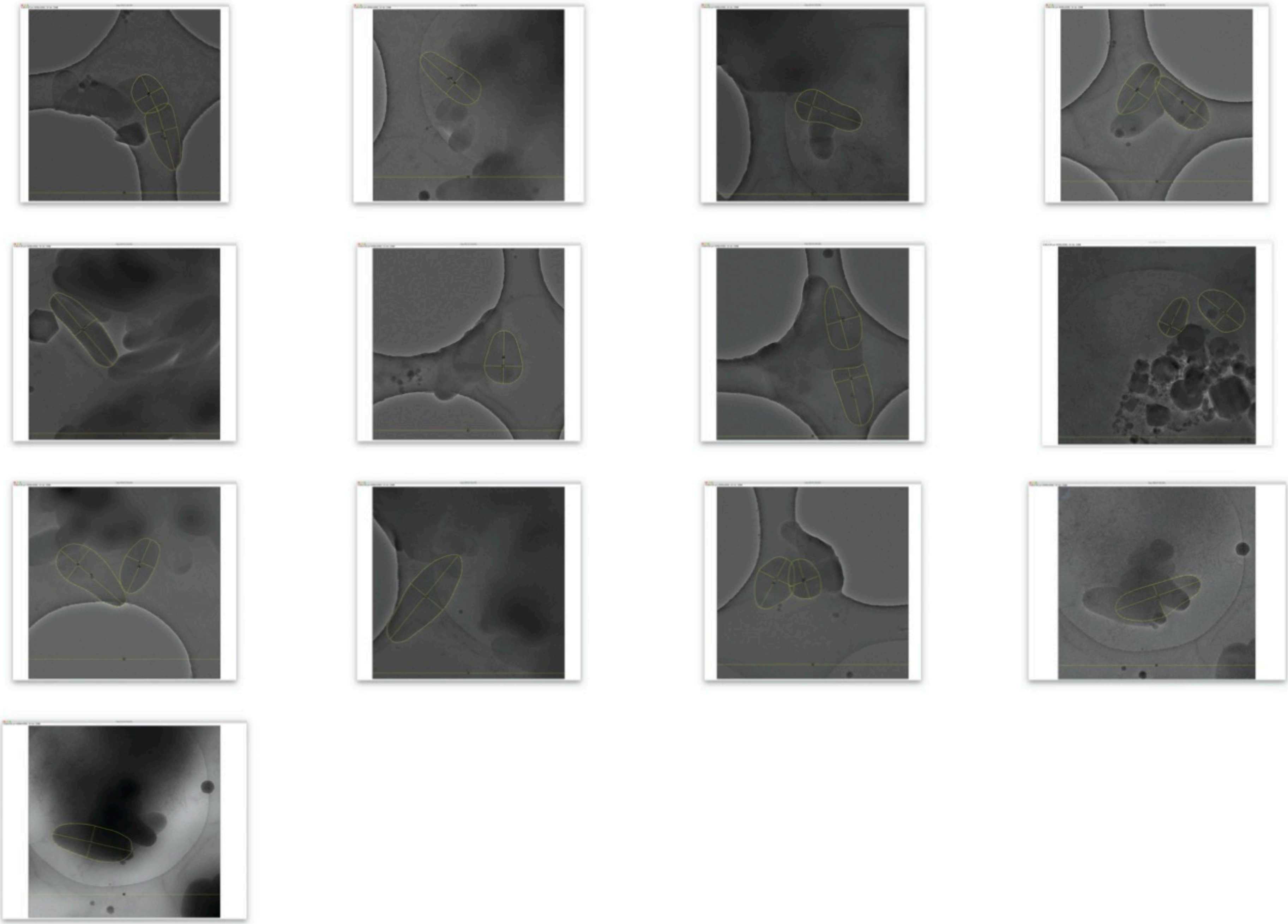

Mycobacteroides immunogenum

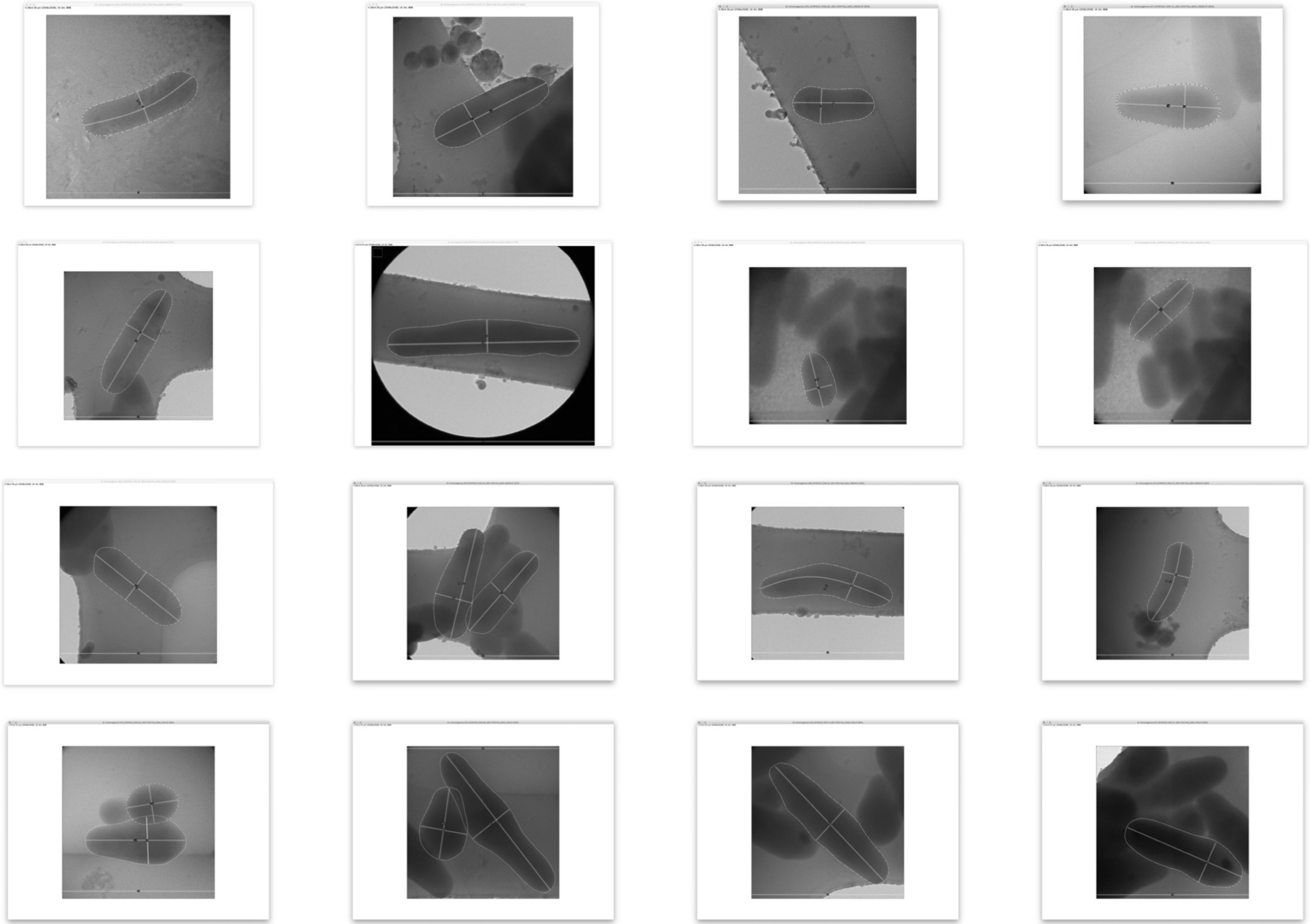

Mycobacteroides immunogenum

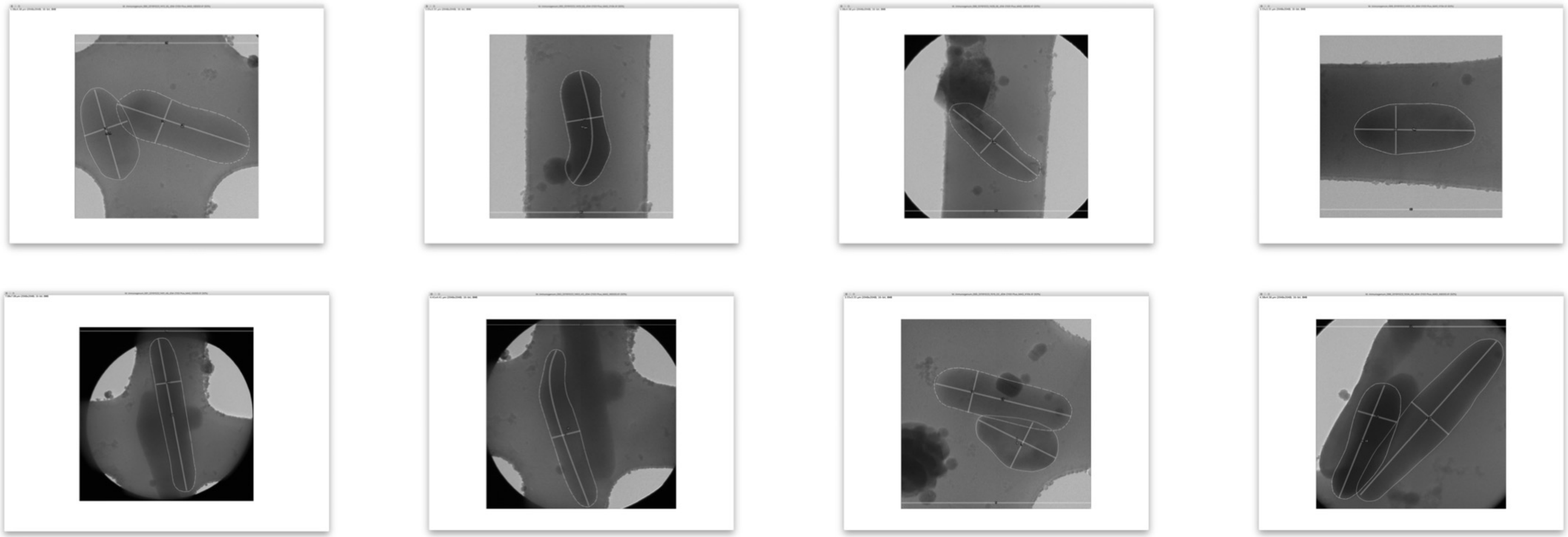

Mycobacteroides salmoniphilum

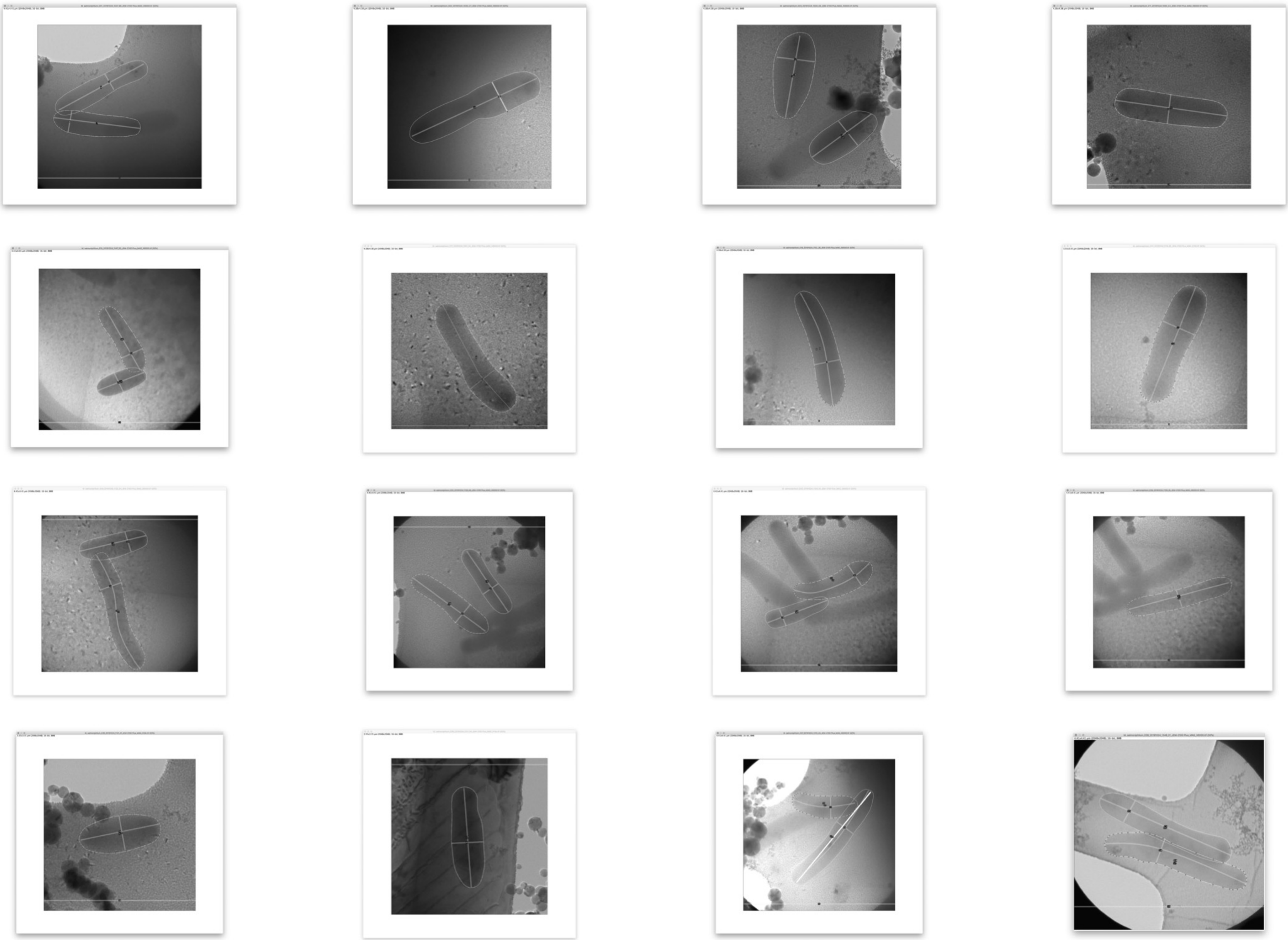

Mycobacteroides salmoniphilum

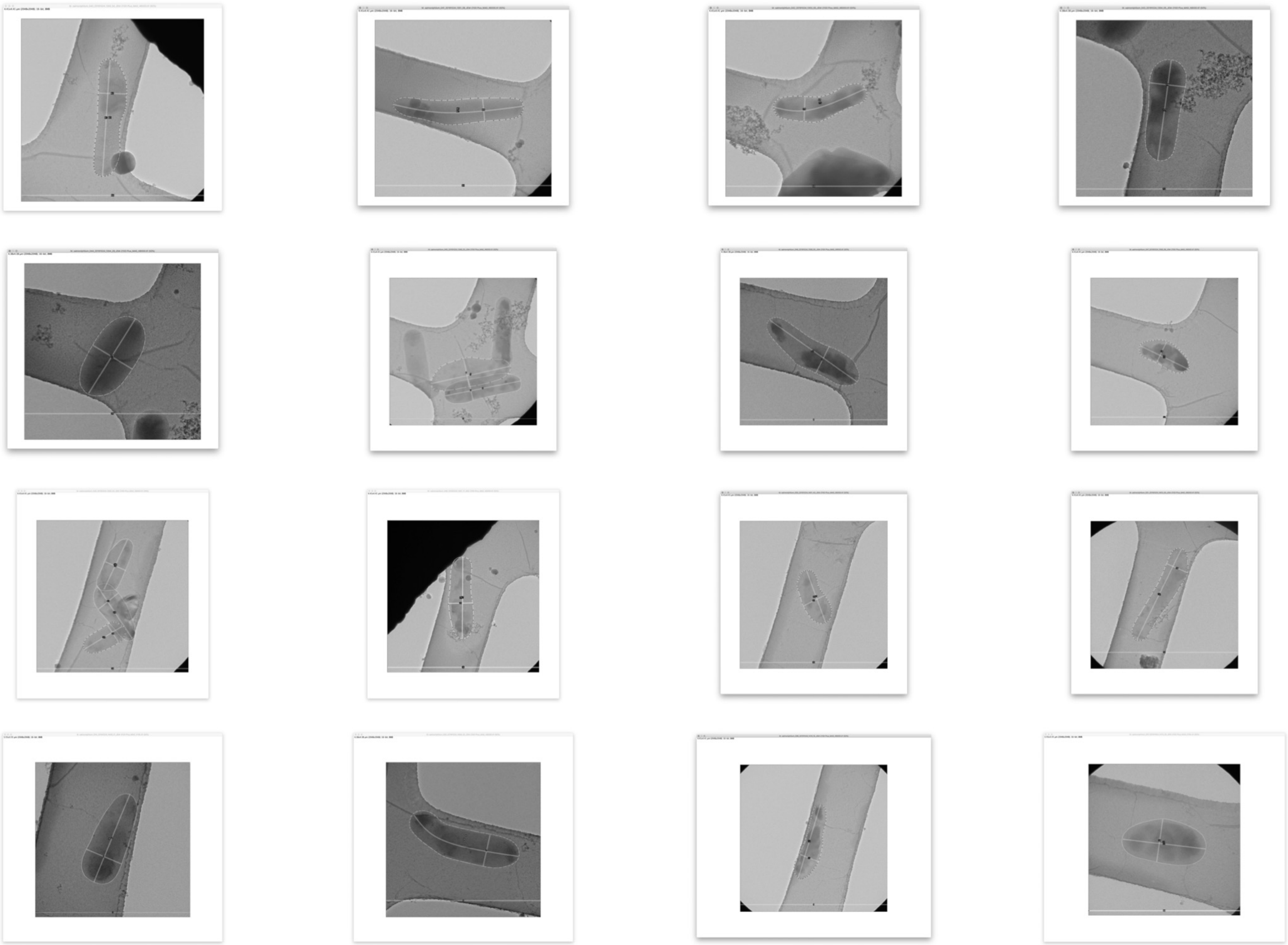

Mycobacteroides salmoniphilum

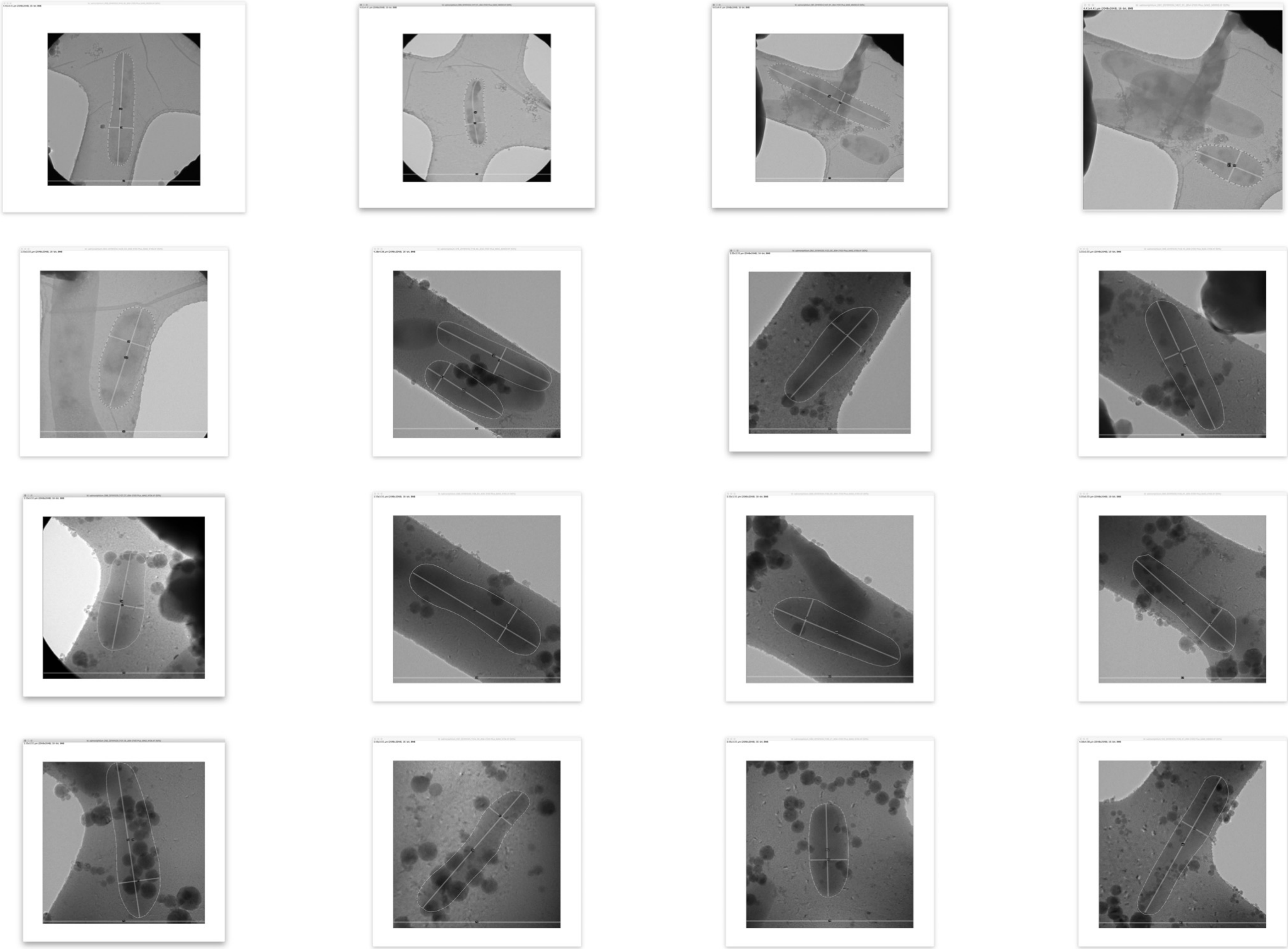

Mycobacteroides salmoniphilum

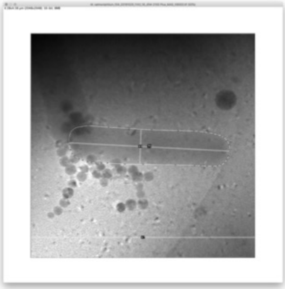

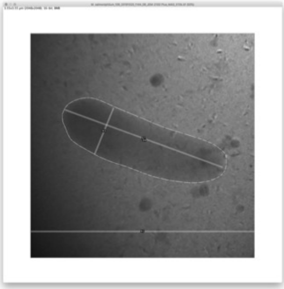

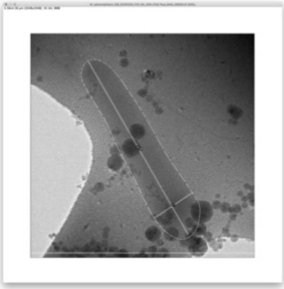

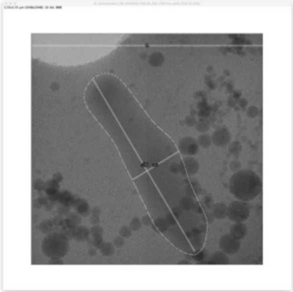

Supplement: Supplementary file 2 [file Data_Sheet_2.PDF]
