# Supplementary material for: Fundamental Cell Morphologies Examined With Cryo-TEM of the Species in the Novel Five Genera Robustly Correlate With New Classification in Family Mycobacteriaceae
Source: Front Microbiol. 2020 Nov 16;11:562395. doi: 10.3389/fmicb.2020.562395 (PMC7701246; doi:10.3389/fmicb.2020.562395)

Supplementary Figure S 5 *Genus Mycolicibacterium*

Mycolicibacterium aurum

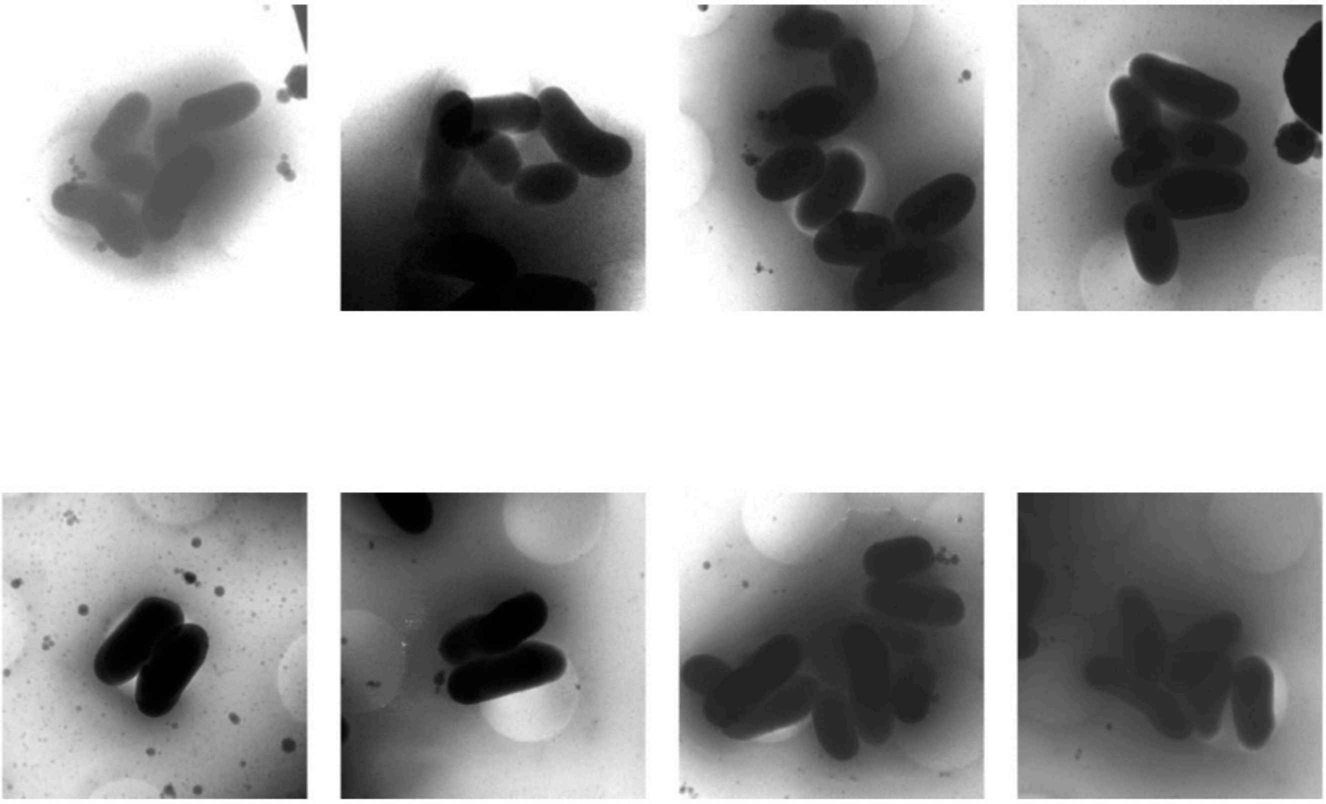

Mycolicibacterium austroafricanum

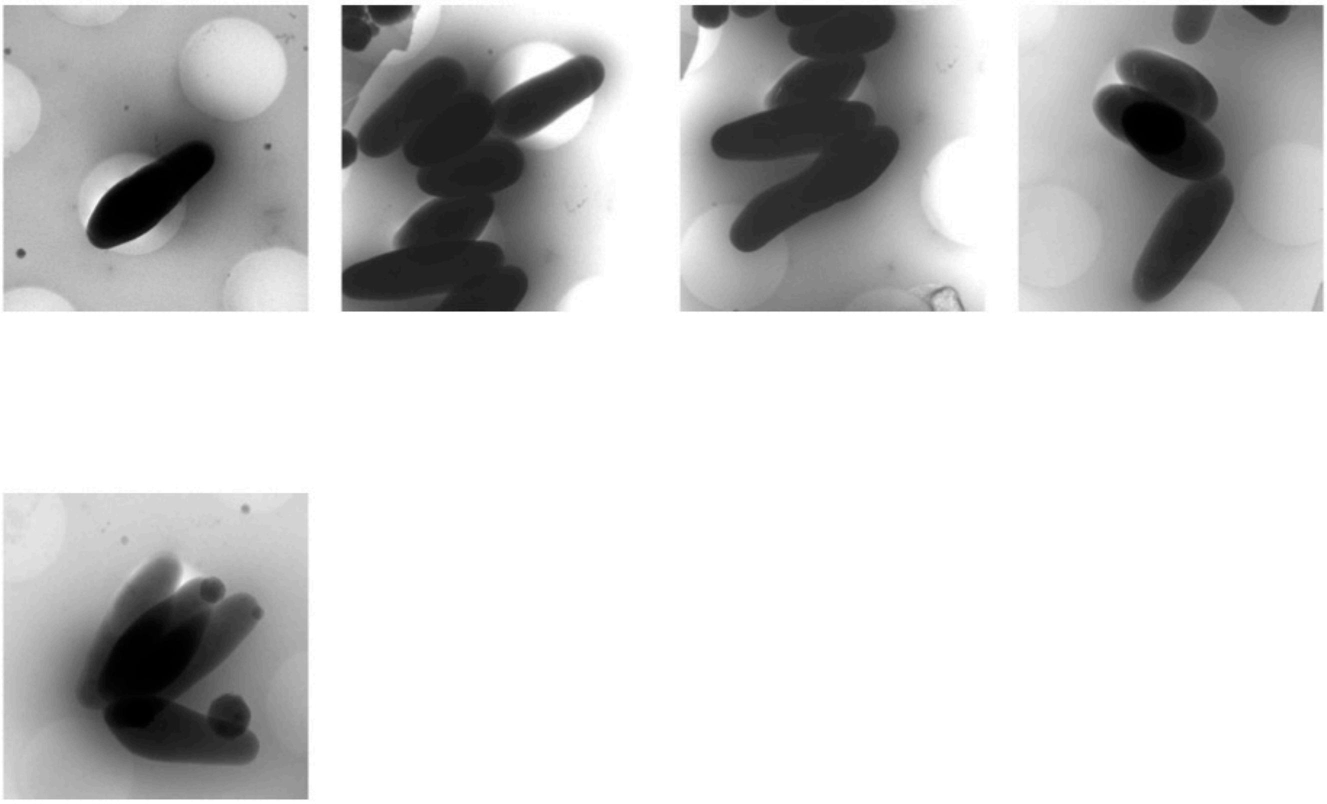

Mycolicibacterium chitae

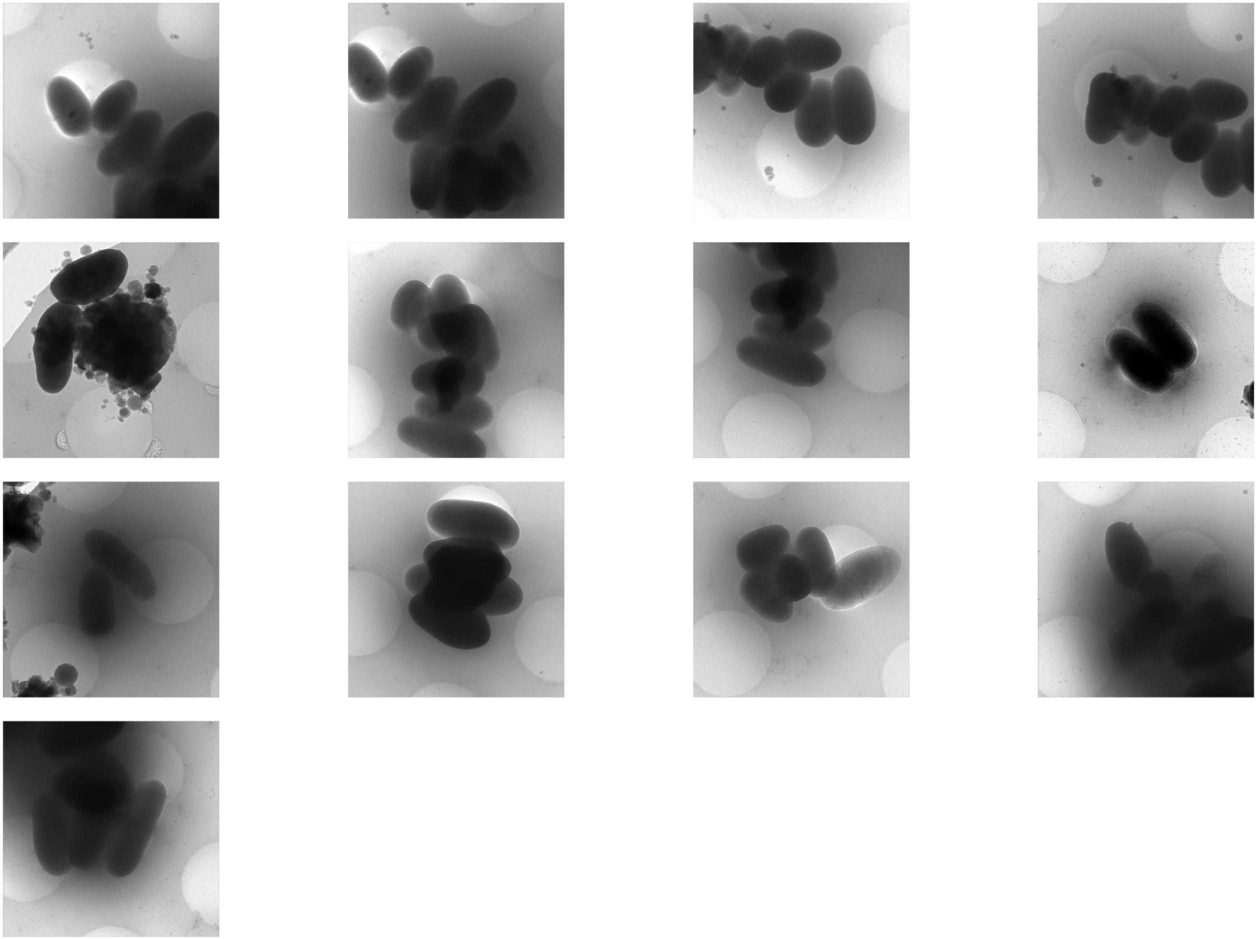

Mycolicibacterium fortuitum subsp. fortuitum (ATCC 6841)

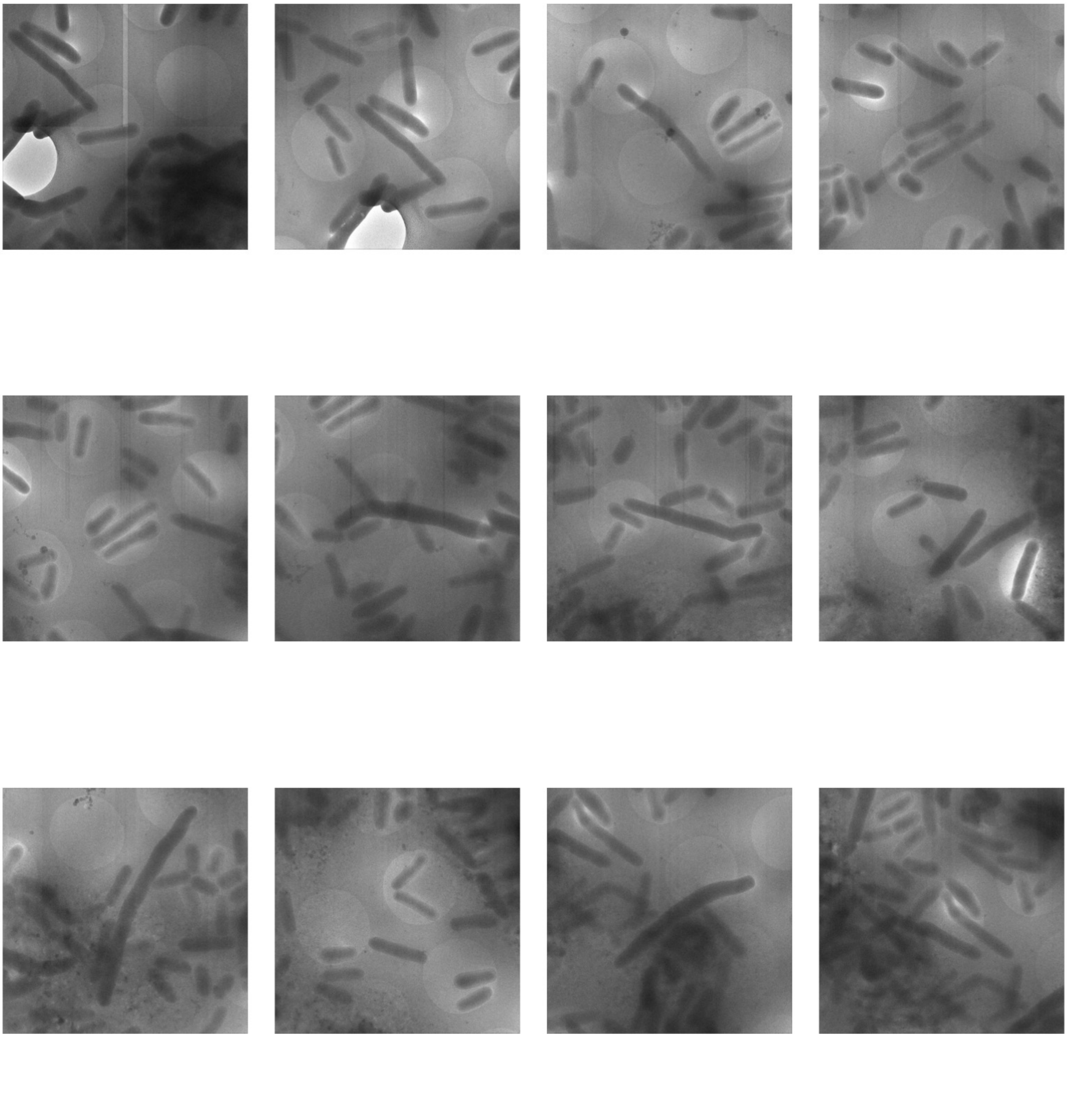

Mycolicibacterium fortuitum subsp. fortuitum (ATCC 6841)

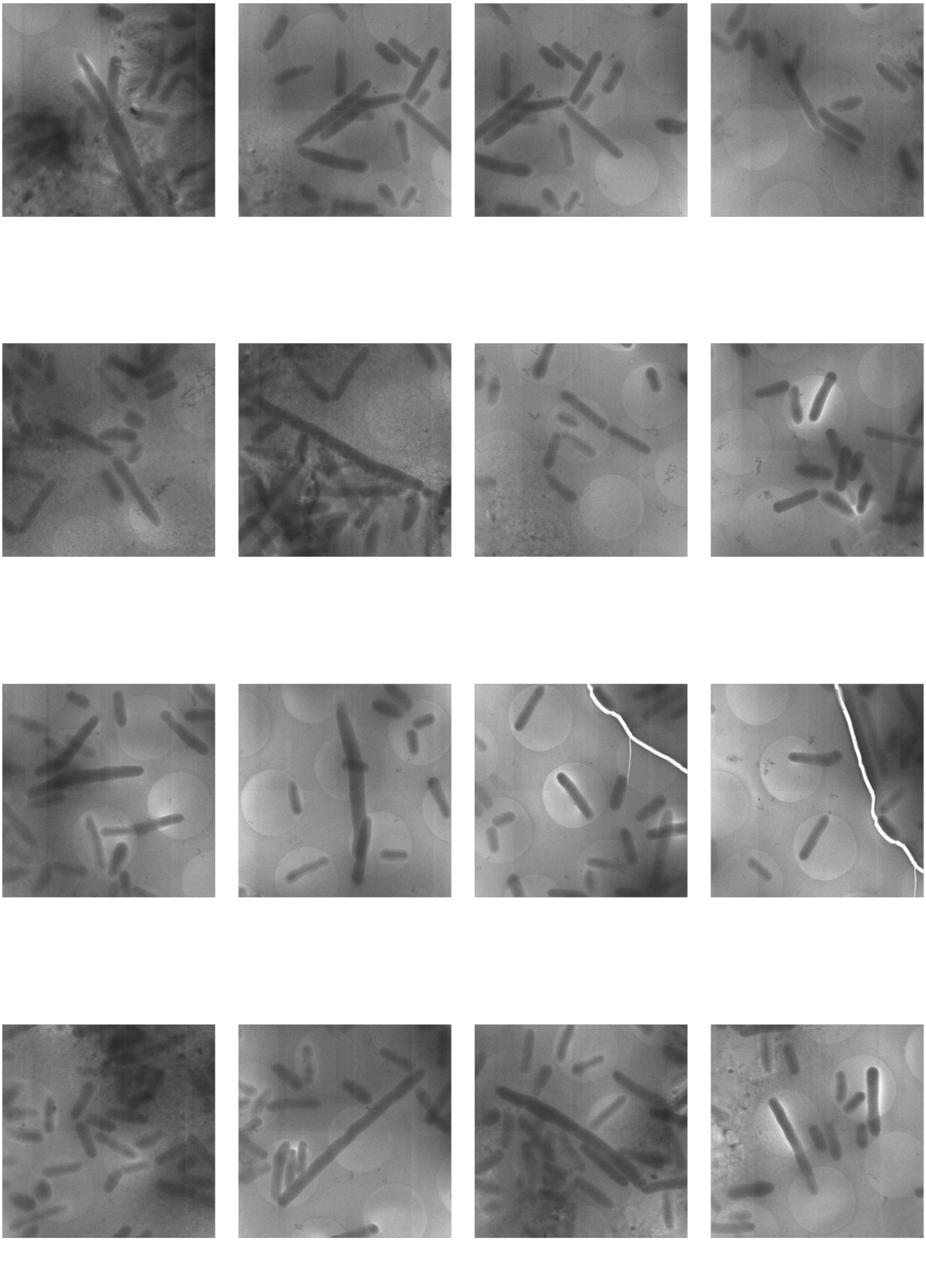

Mycolicibacterium fortuitum subsp. fortuitum (ATCC 6841)

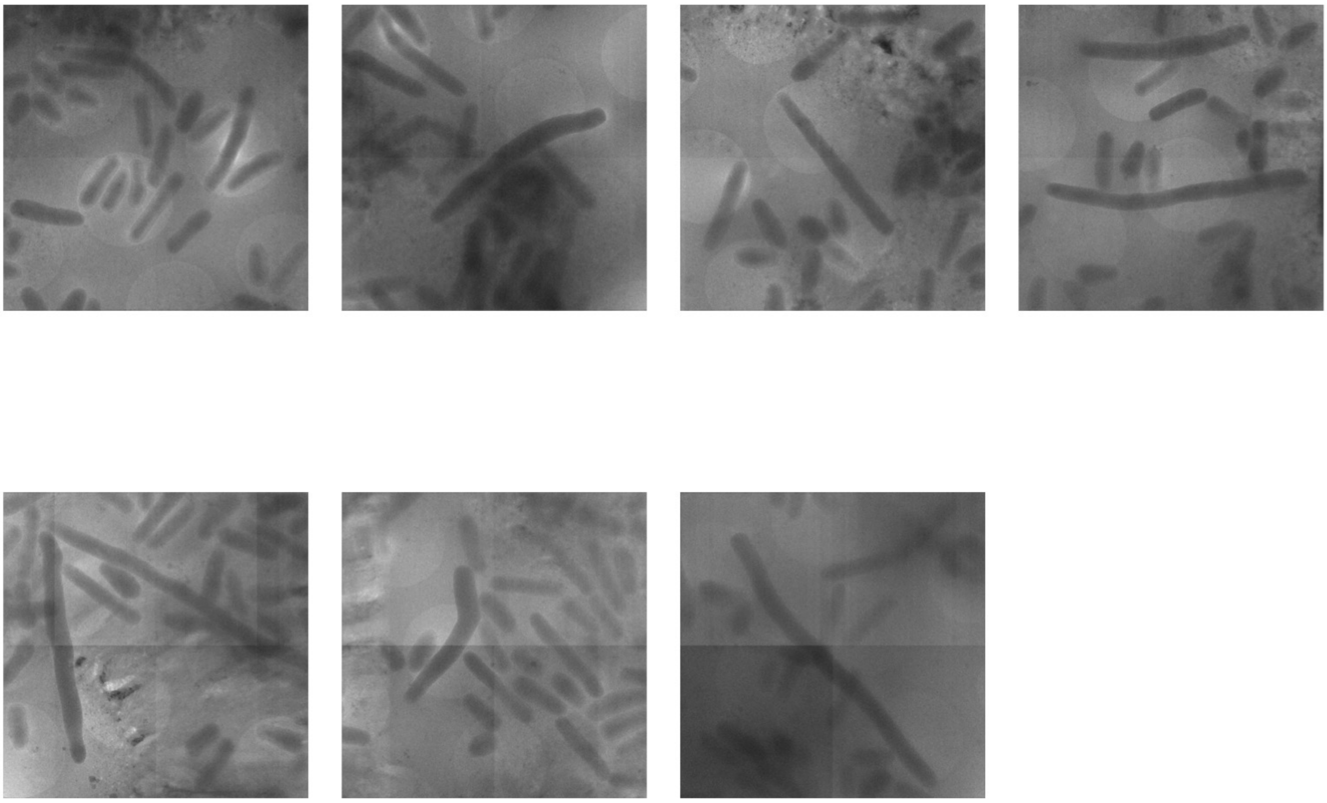

Mycolicibacterium fortuitum subsp. fortuitum (ATCC 11440)

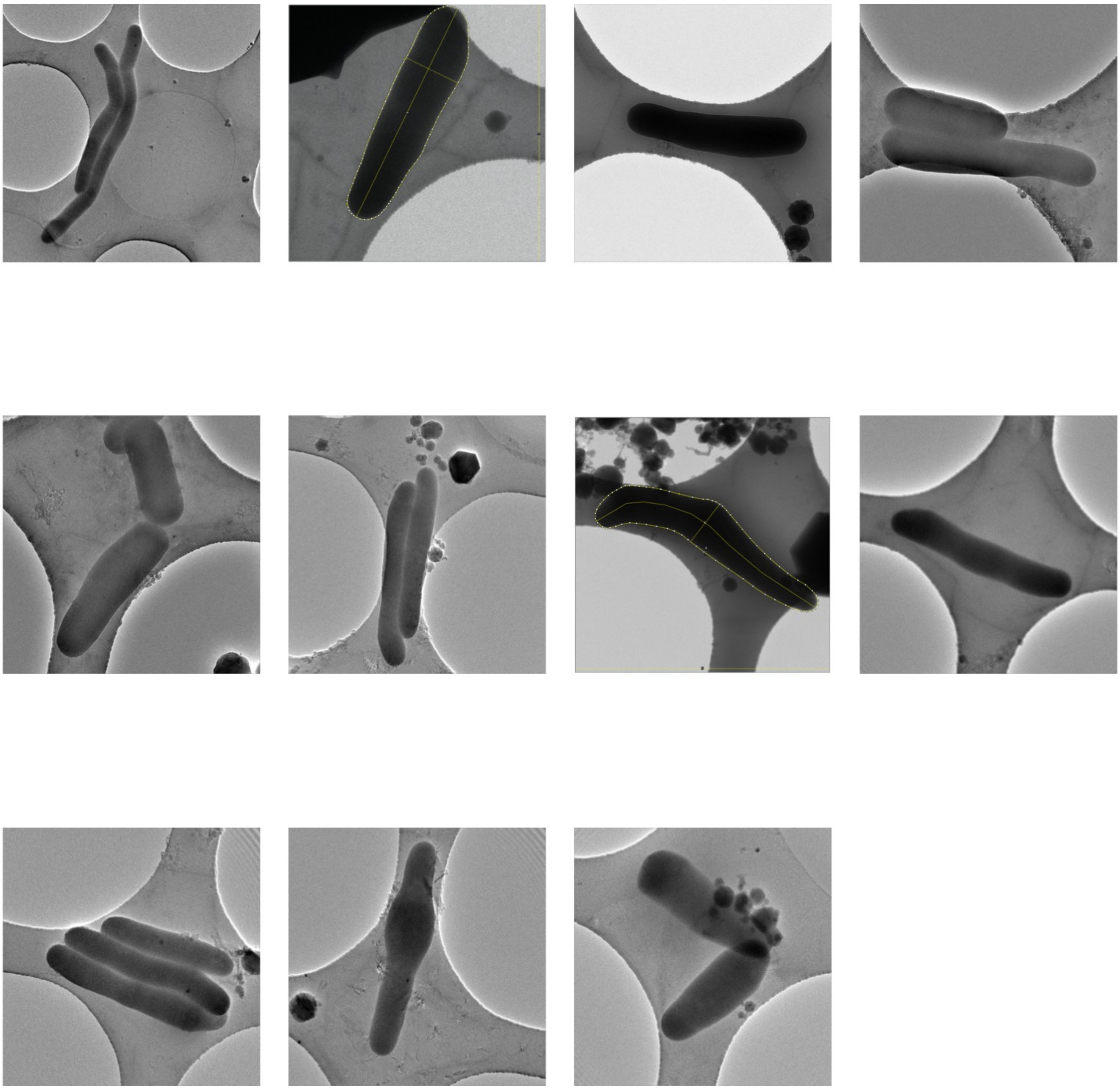

Mycolicibacterium gilvum

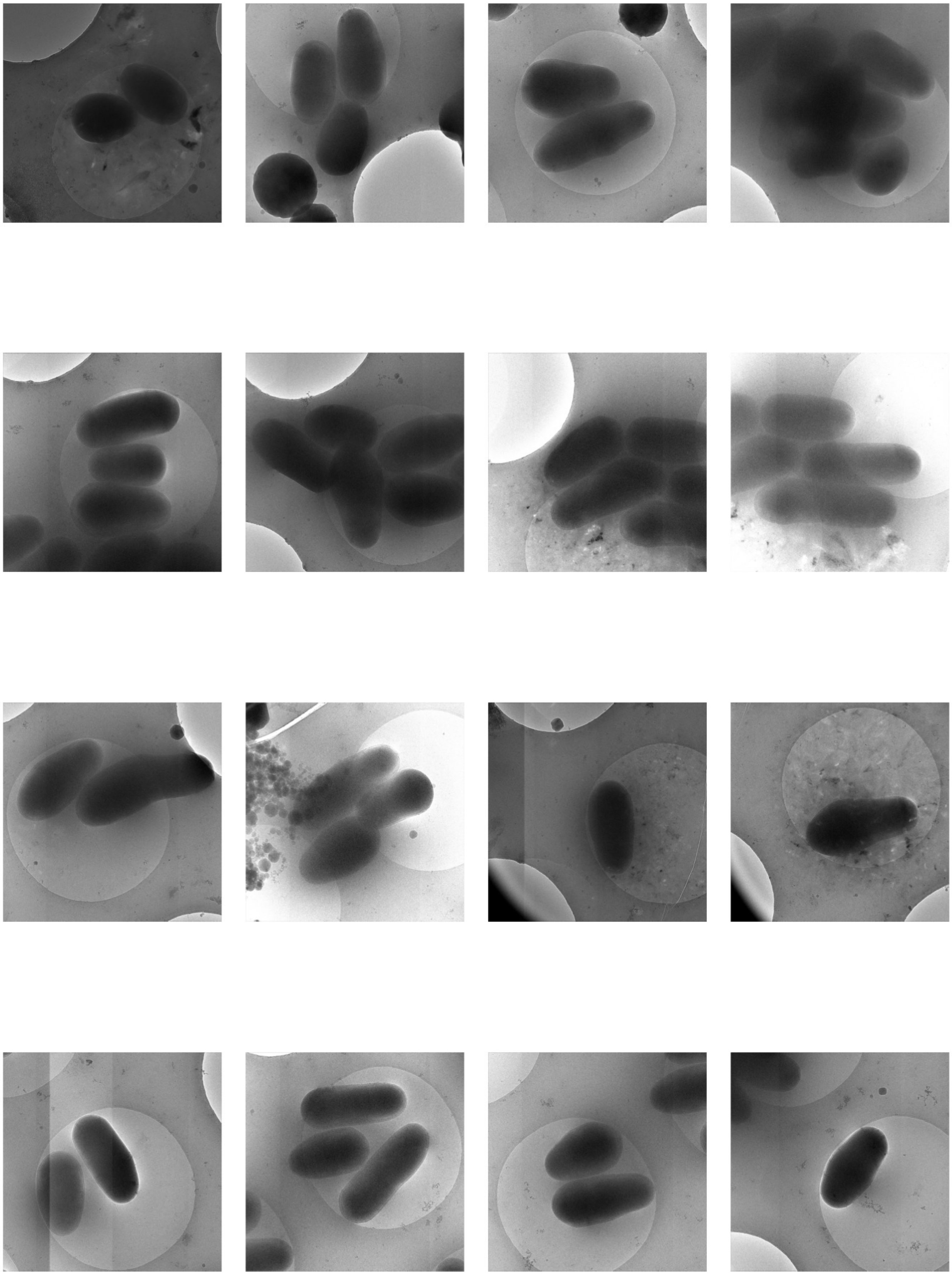

Mycolicibacterium gilvum

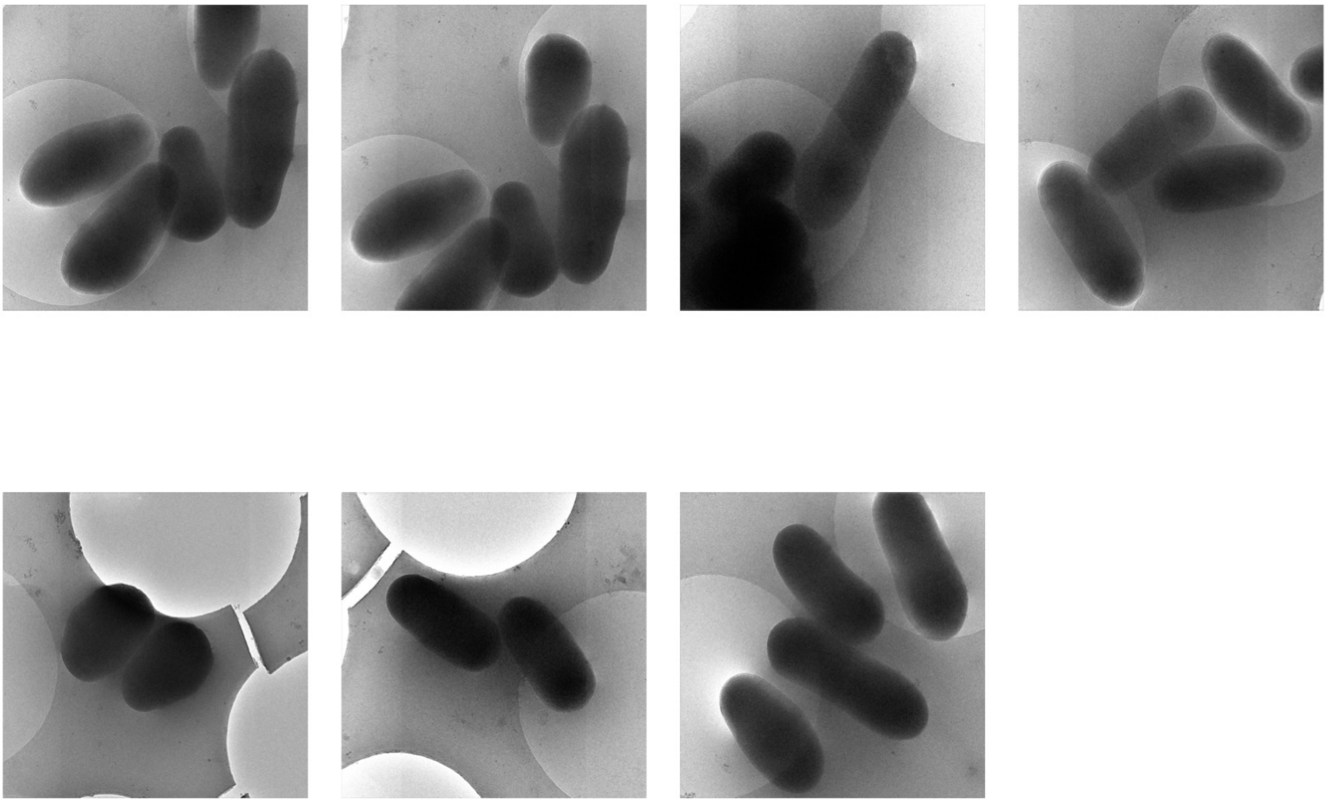

Mycolicibacterium smegmatis

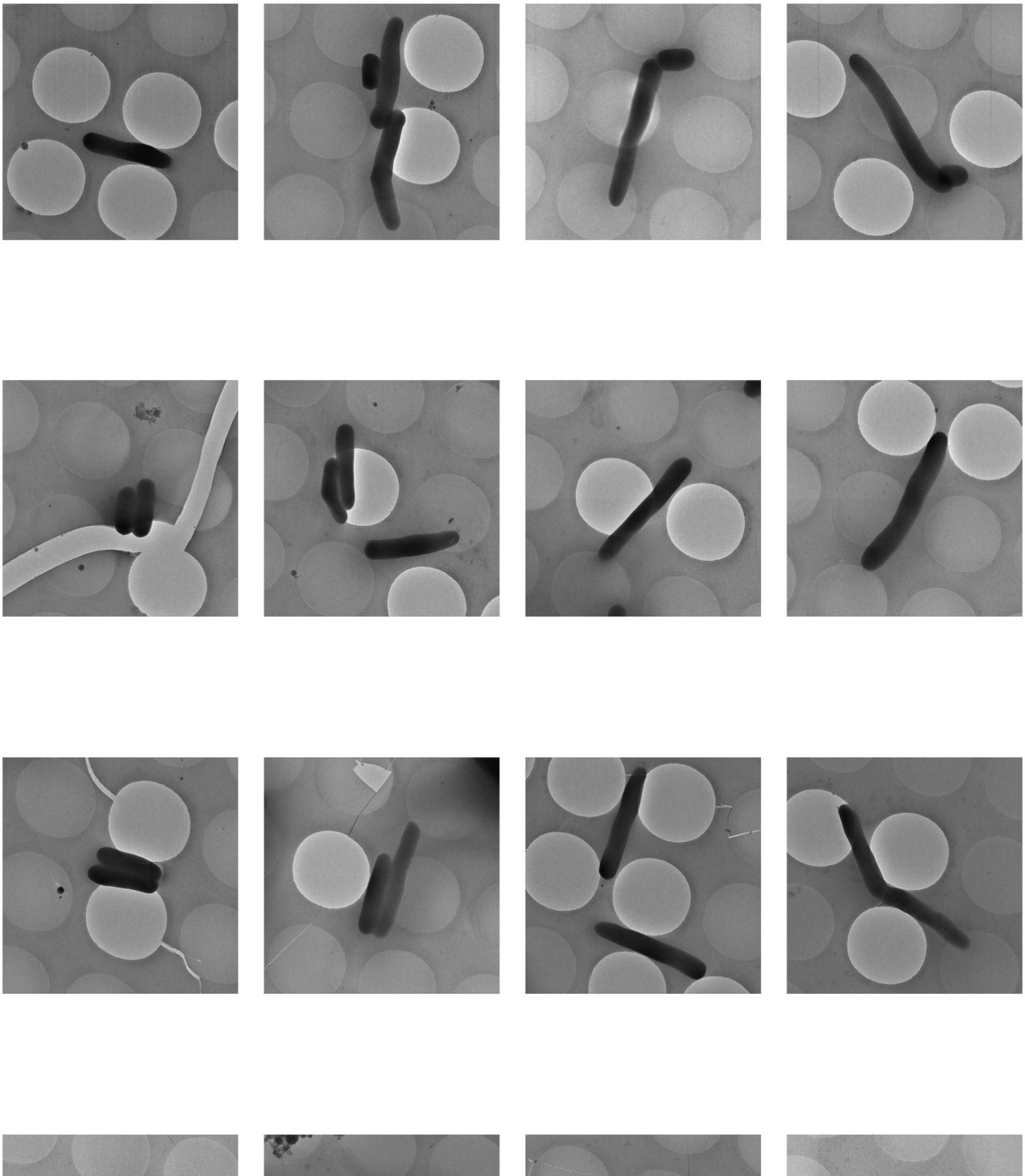

Mycolicibacterium smegmatis

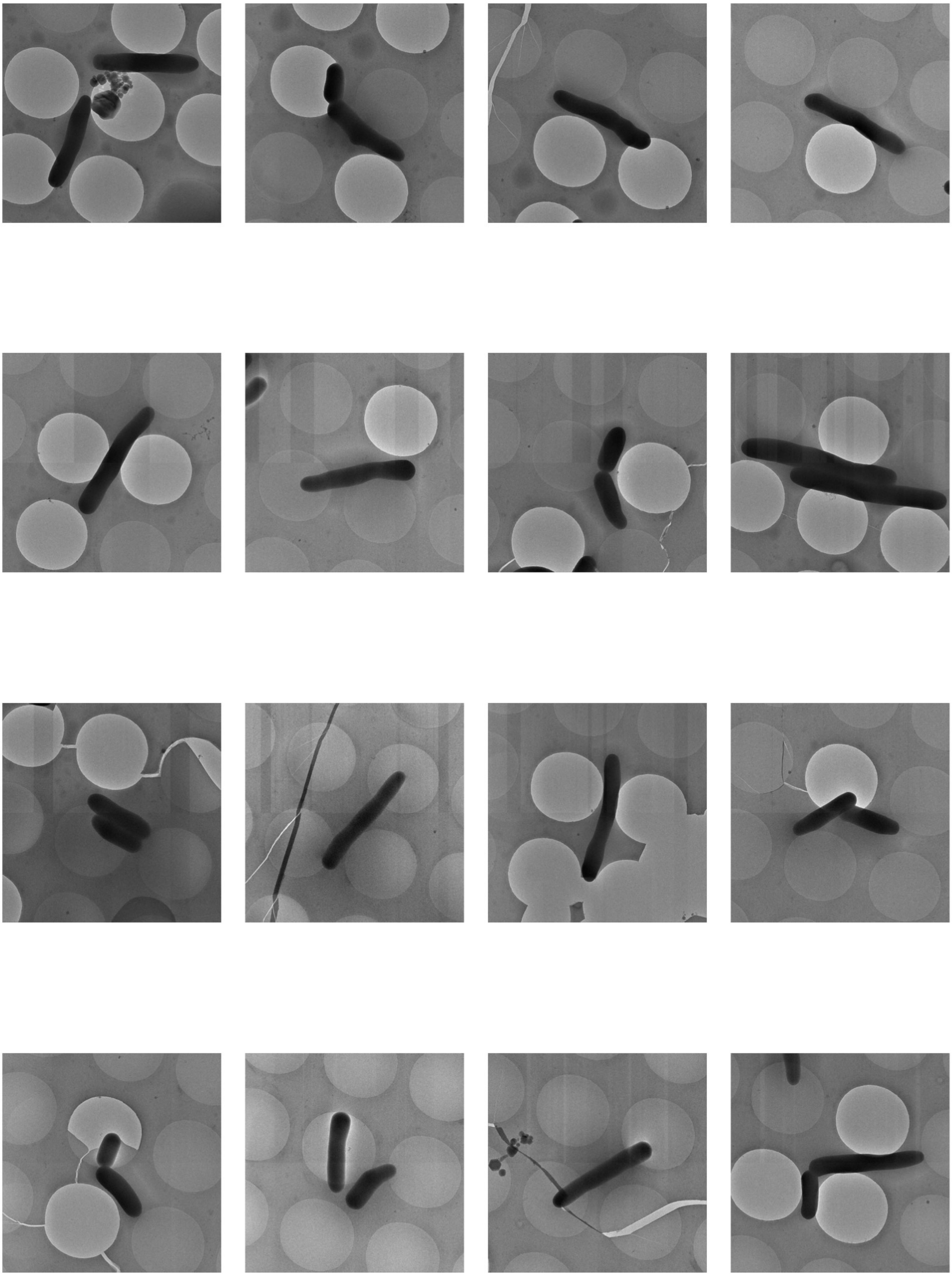

Mycolicibacterium smegmatis

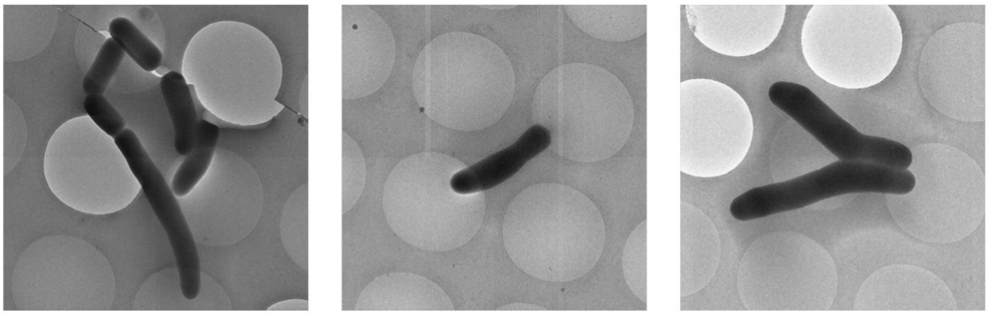

Supplement: Supplementary file 3 [file Data_Sheet_3.PDF]
