# Supplementary material for: Fundamental Cell Morphologies Examined With Cryo-TEM of the Species in the Novel Five Genera Robustly Correlate With New Classification in Family Mycobacteriaceae
Source: Front Microbiol. 2020 Nov 16;11:562395. doi: 10.3389/fmicb.2020.562395 (PMC7701246; doi:10.3389/fmicb.2020.562395)

Supplementary Figure 8

Genus Mycolicibacillus

Mycolicibacillus koreensis

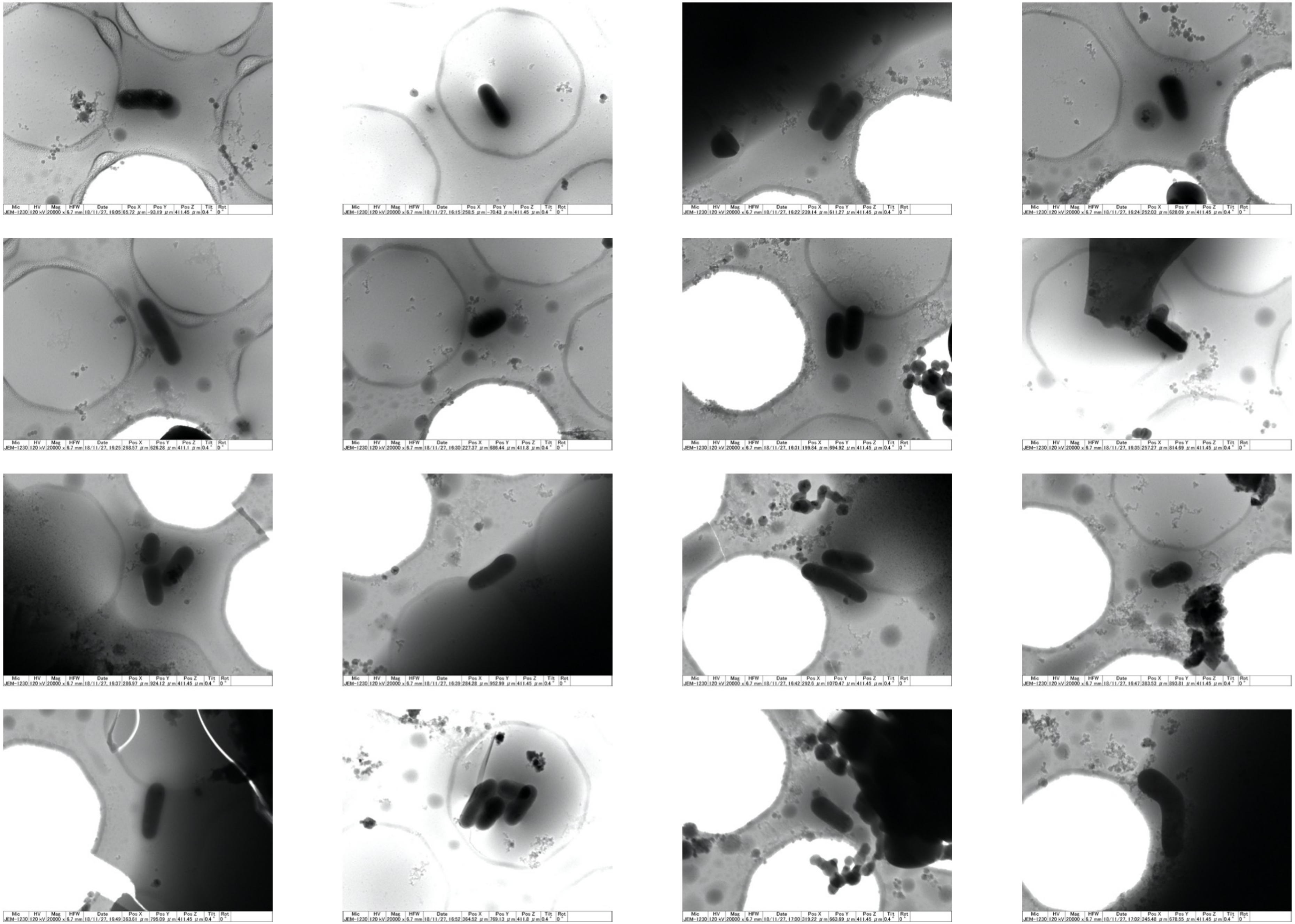

Mycolicibacillus koreensis

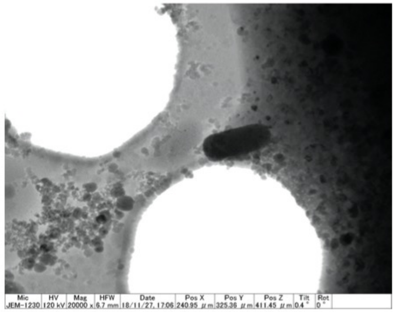

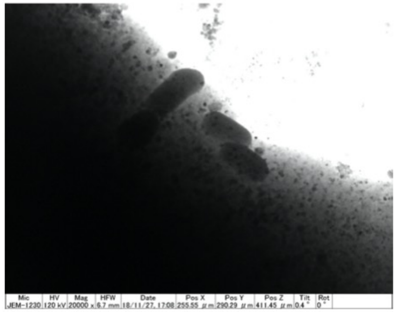

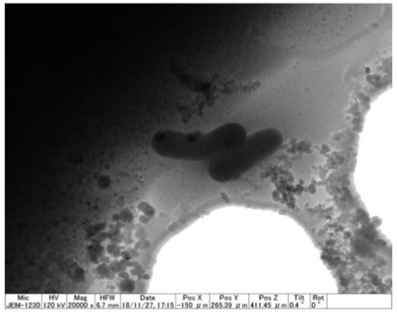

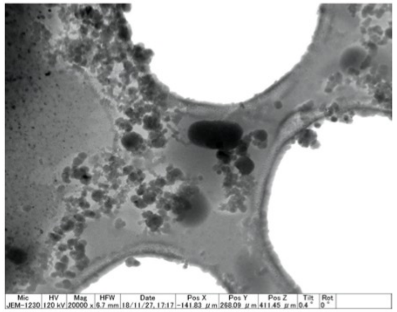

Mycolicibacillus parakoreensis

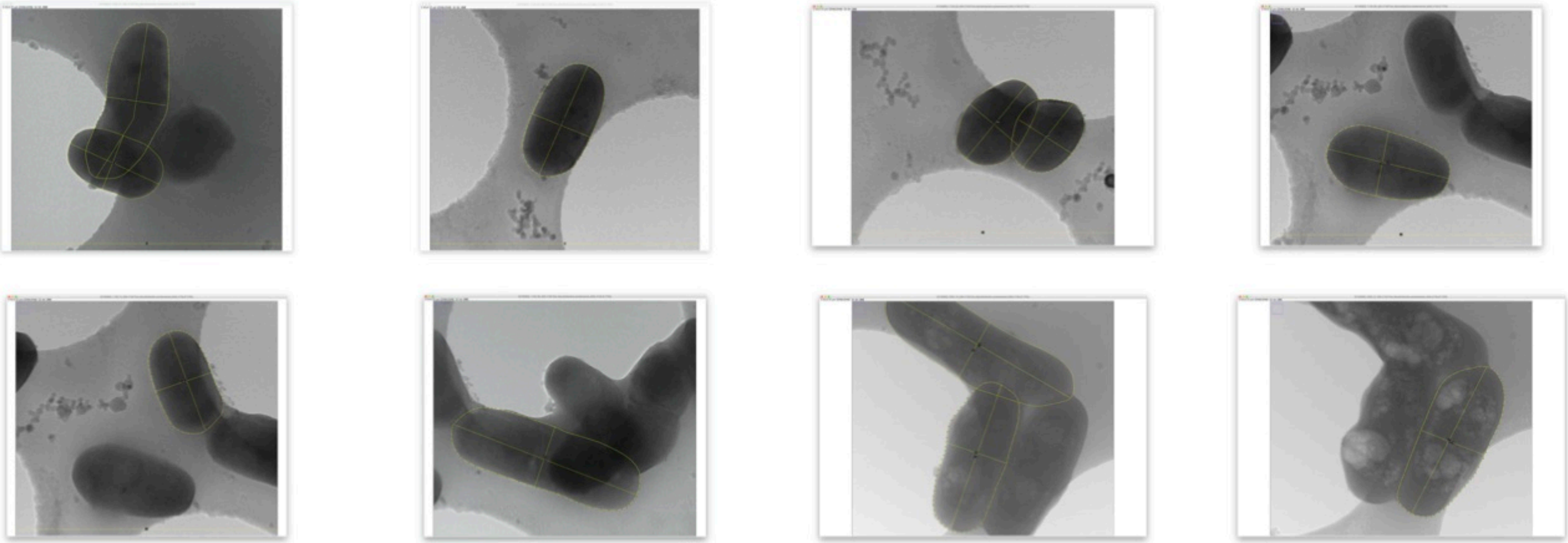

Mycolicibacillus trivialis

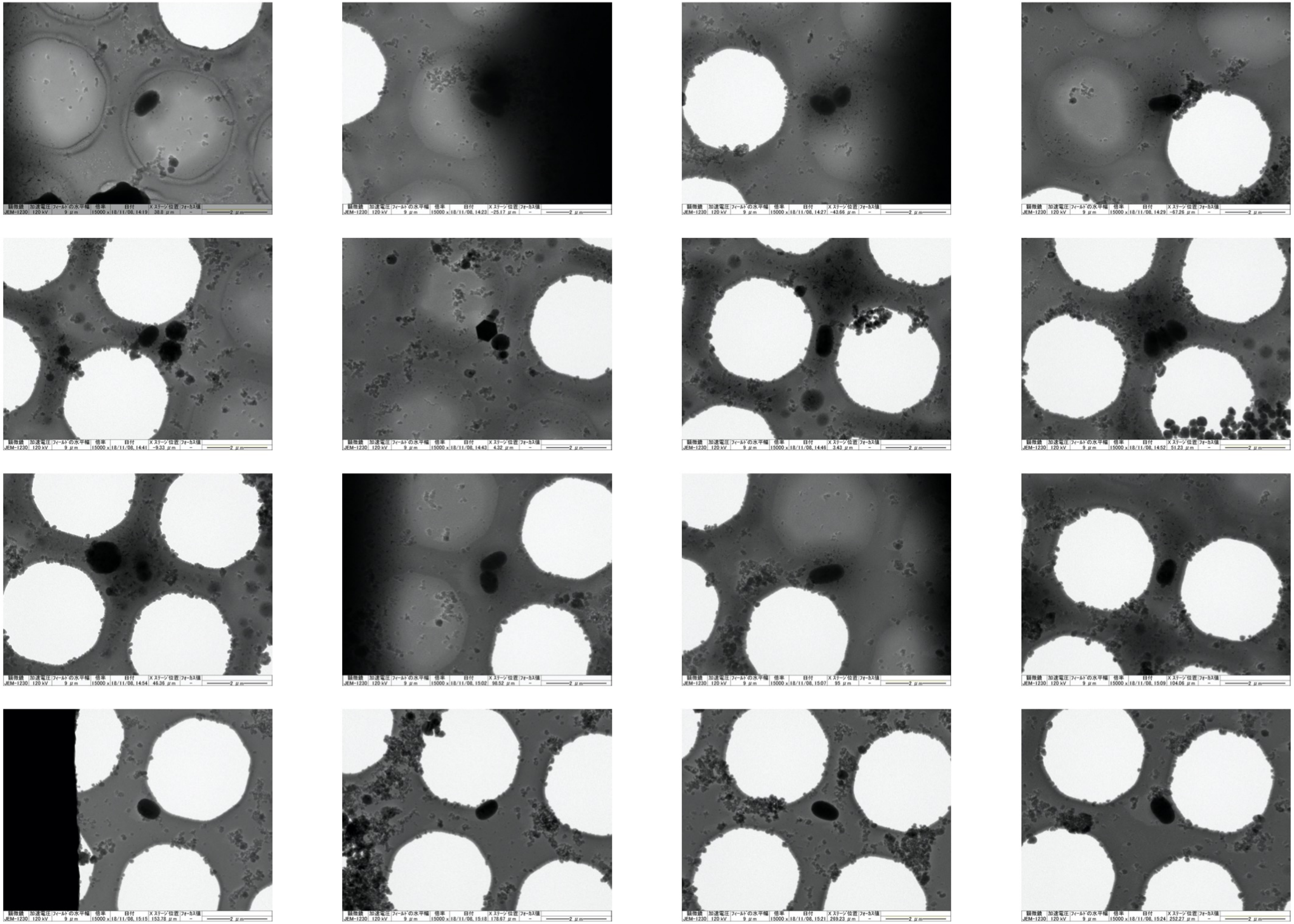

Mycolicibacillus trivialis

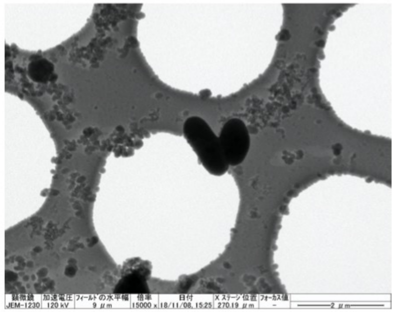

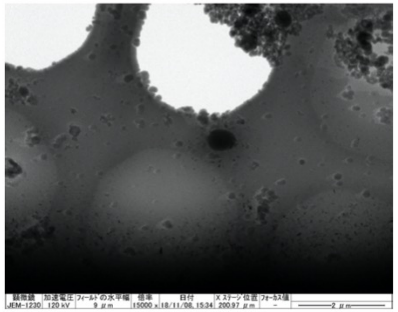

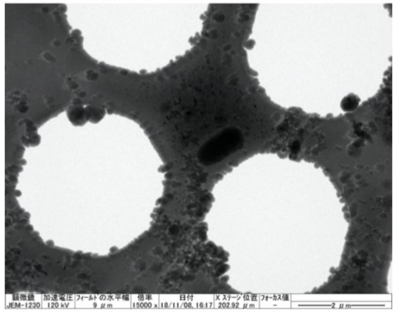

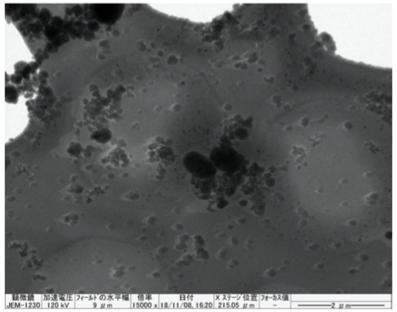

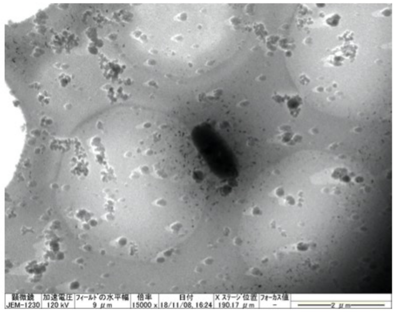

Supplement: Supplementary file 5 [file Data_Sheet_5.PDF]
