# Supplementary material for: Fundamental Cell Morphologies Examined With Cryo-TEM of the Species in the Novel Five Genera Robustly Correlate With New Classification in Family Mycobacteriaceae
Source: Front Microbiol. 2020 Nov 16;11:562395. doi: 10.3389/fmicb.2020.562395 (PMC7701246; doi:10.3389/fmicb.2020.562395)

Supplementary Figure 9

Genus Mycobacterium

Mycobacterium tuberculosis H37Rv

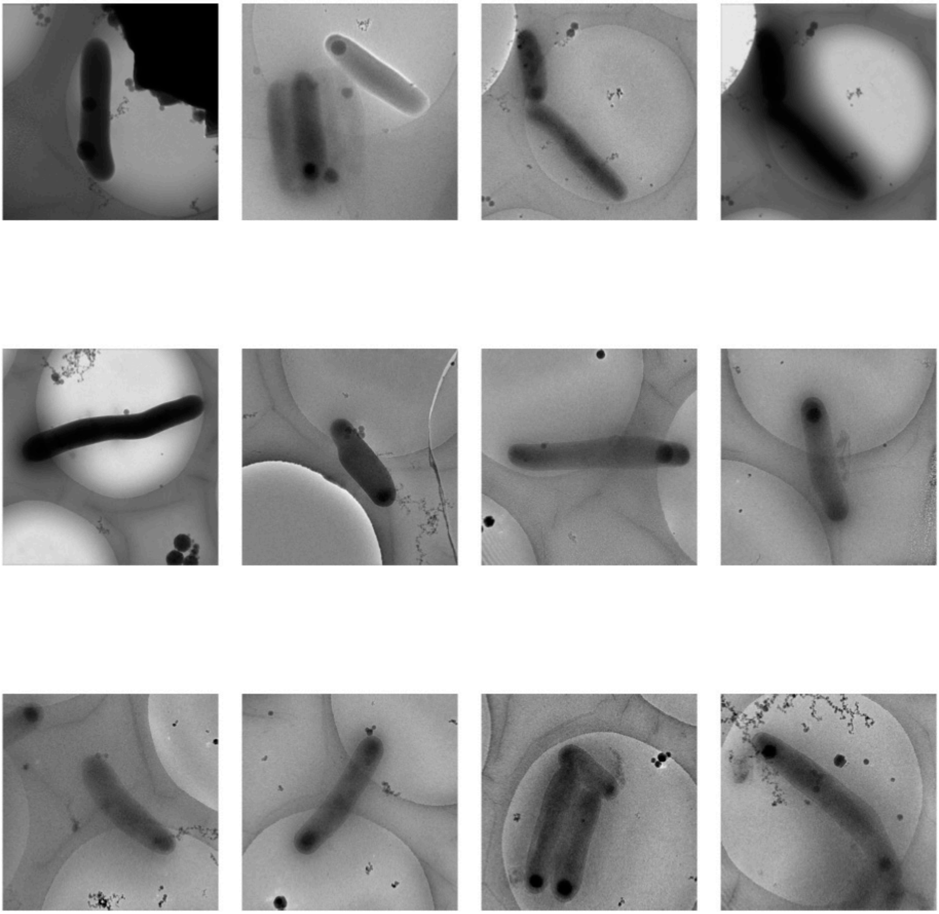

Mycobacterium tuberculosis CDC1551

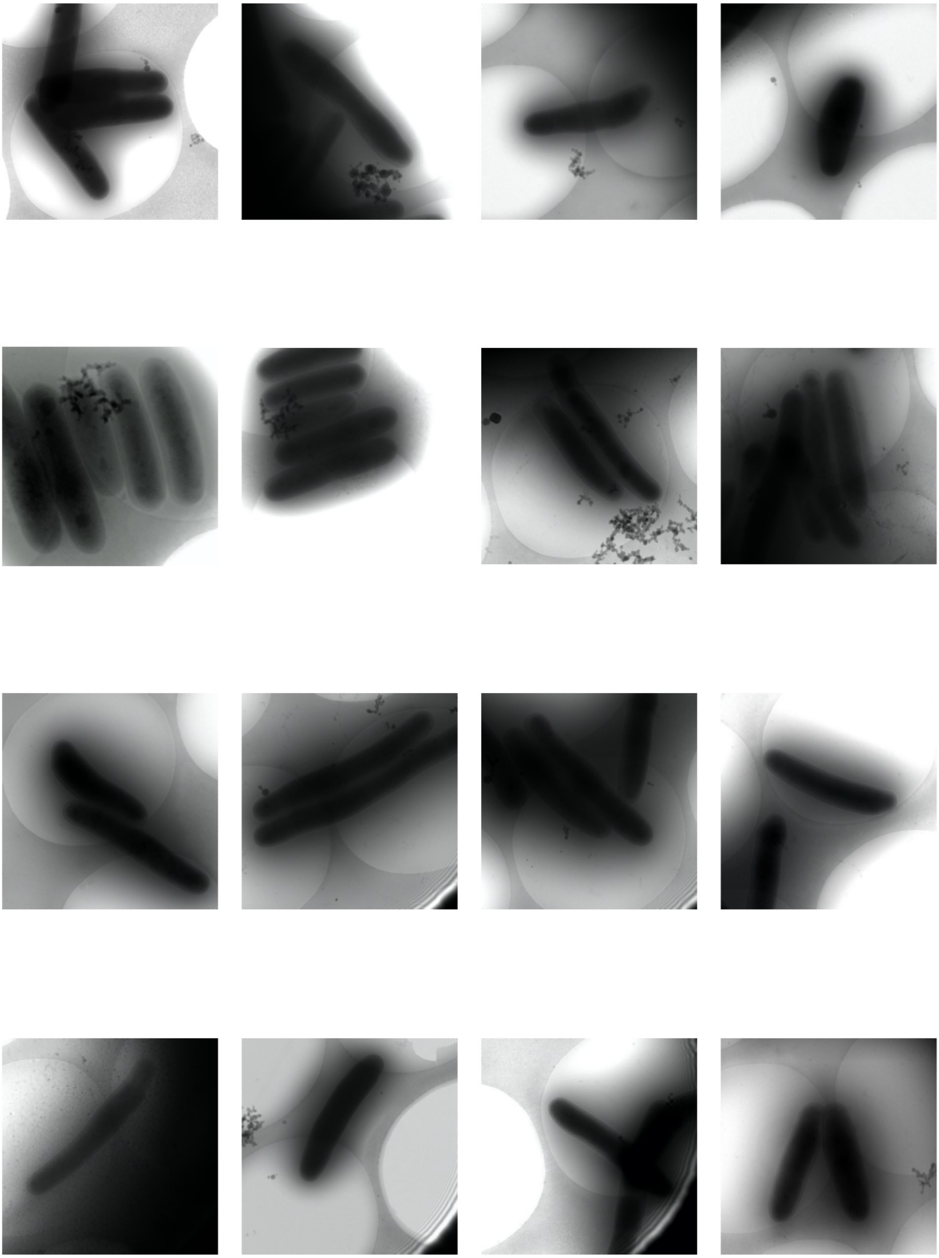

Mycobacterium tuberculosis CDC1551

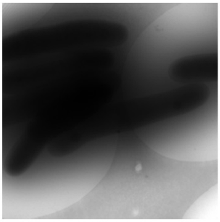

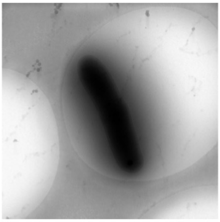

Mycobacterium africanum

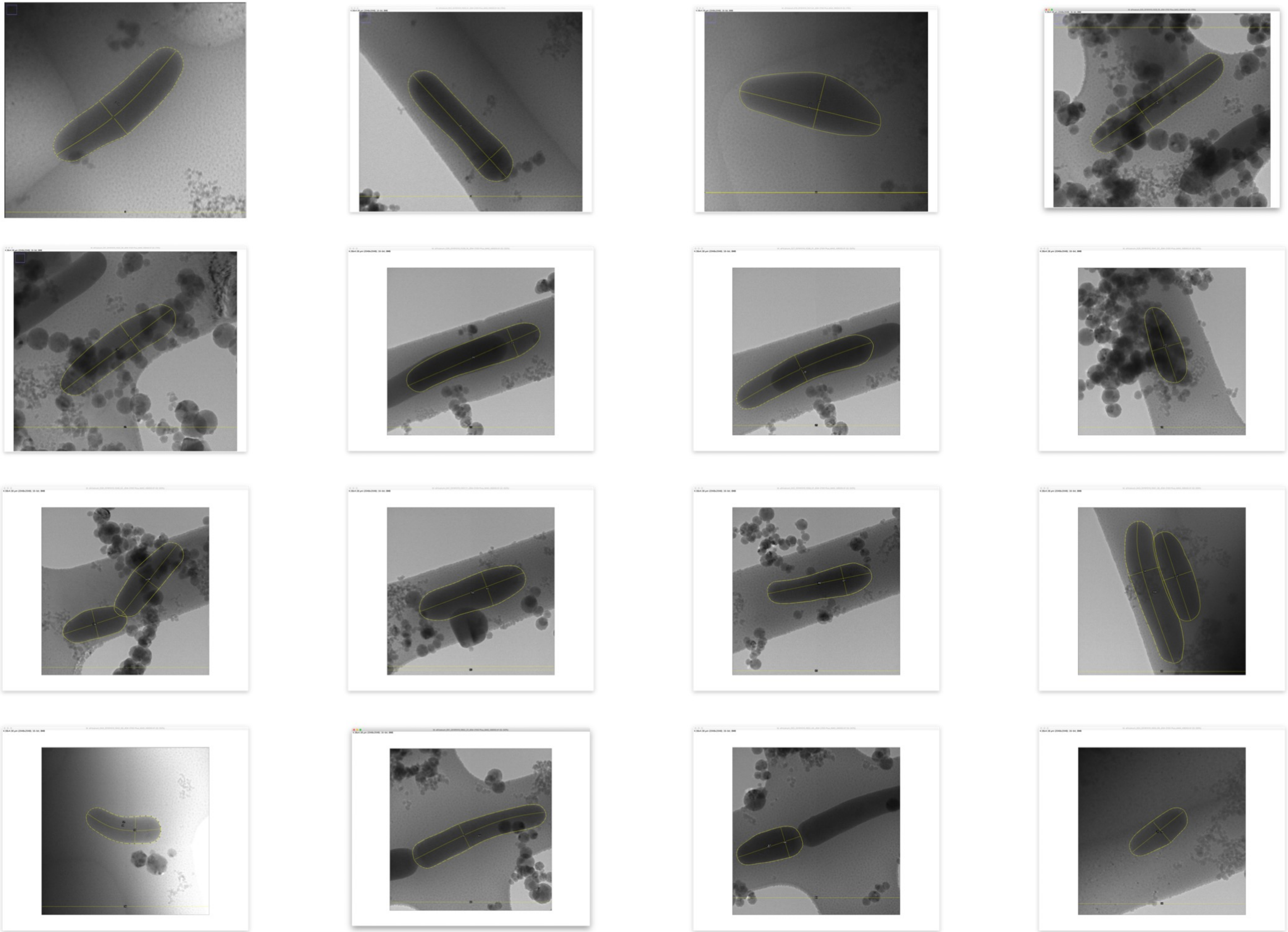

Mycobacterium africanum

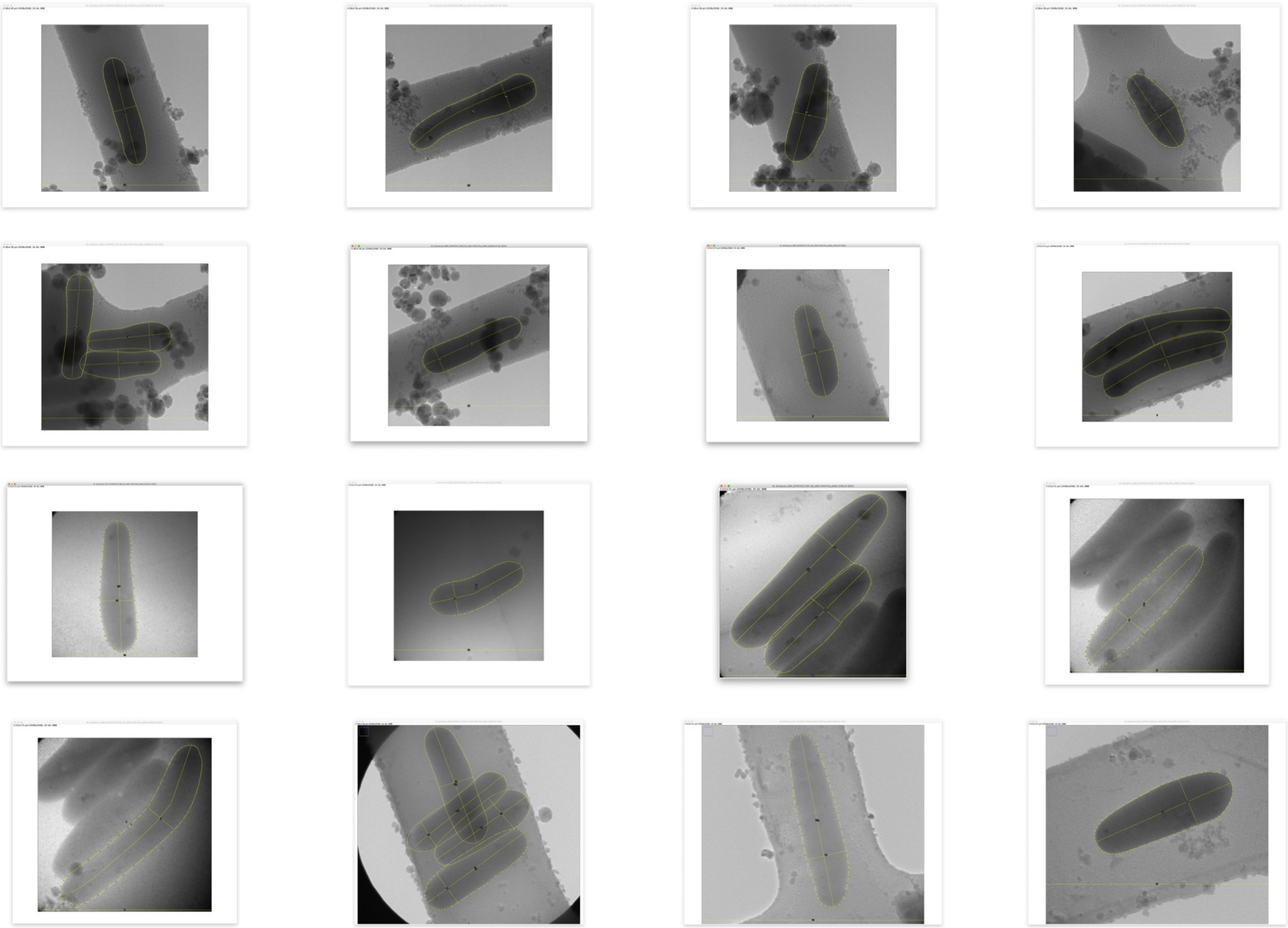

Mycobacterium africanum

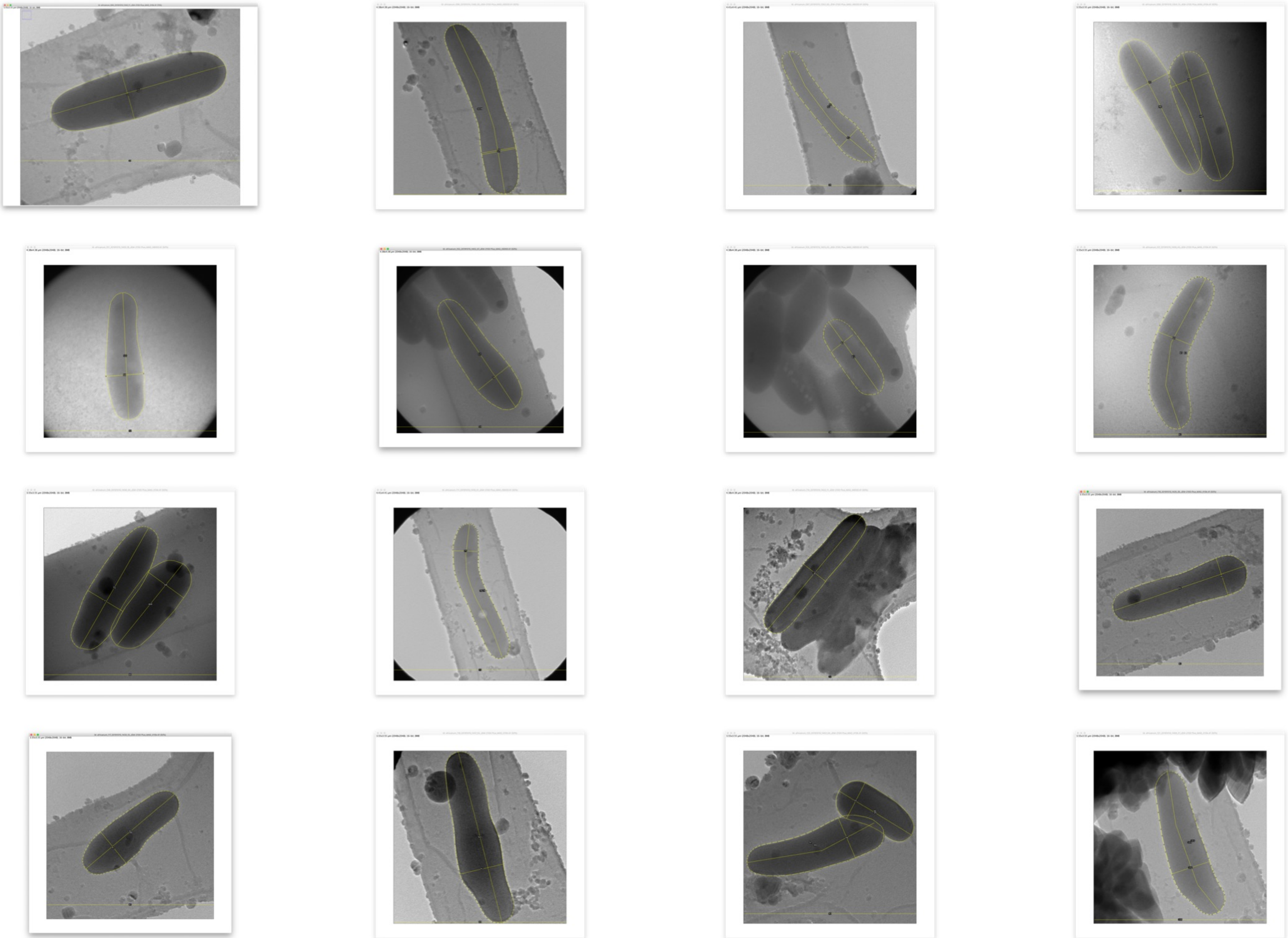

Mycobacterium africanum

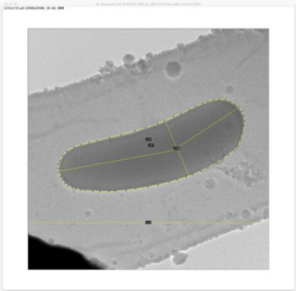

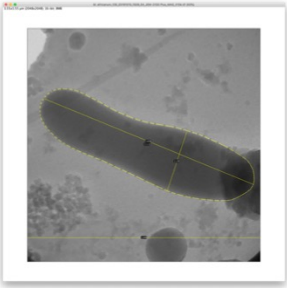

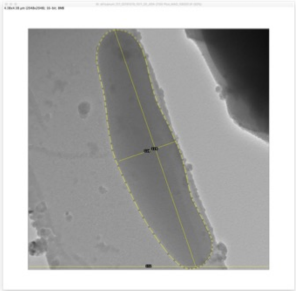

Mycobacterium bovis

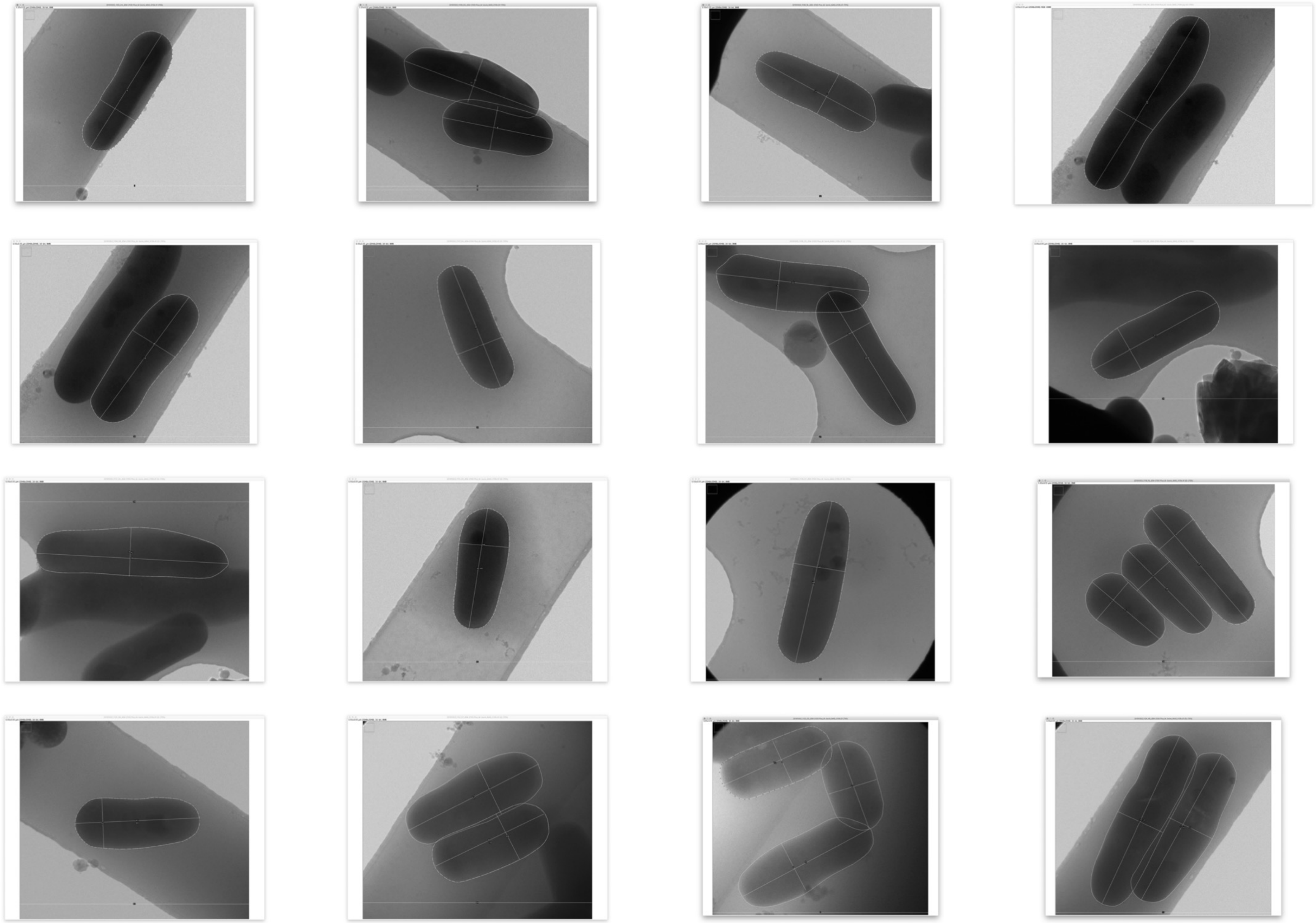

Mycobacterium bovis

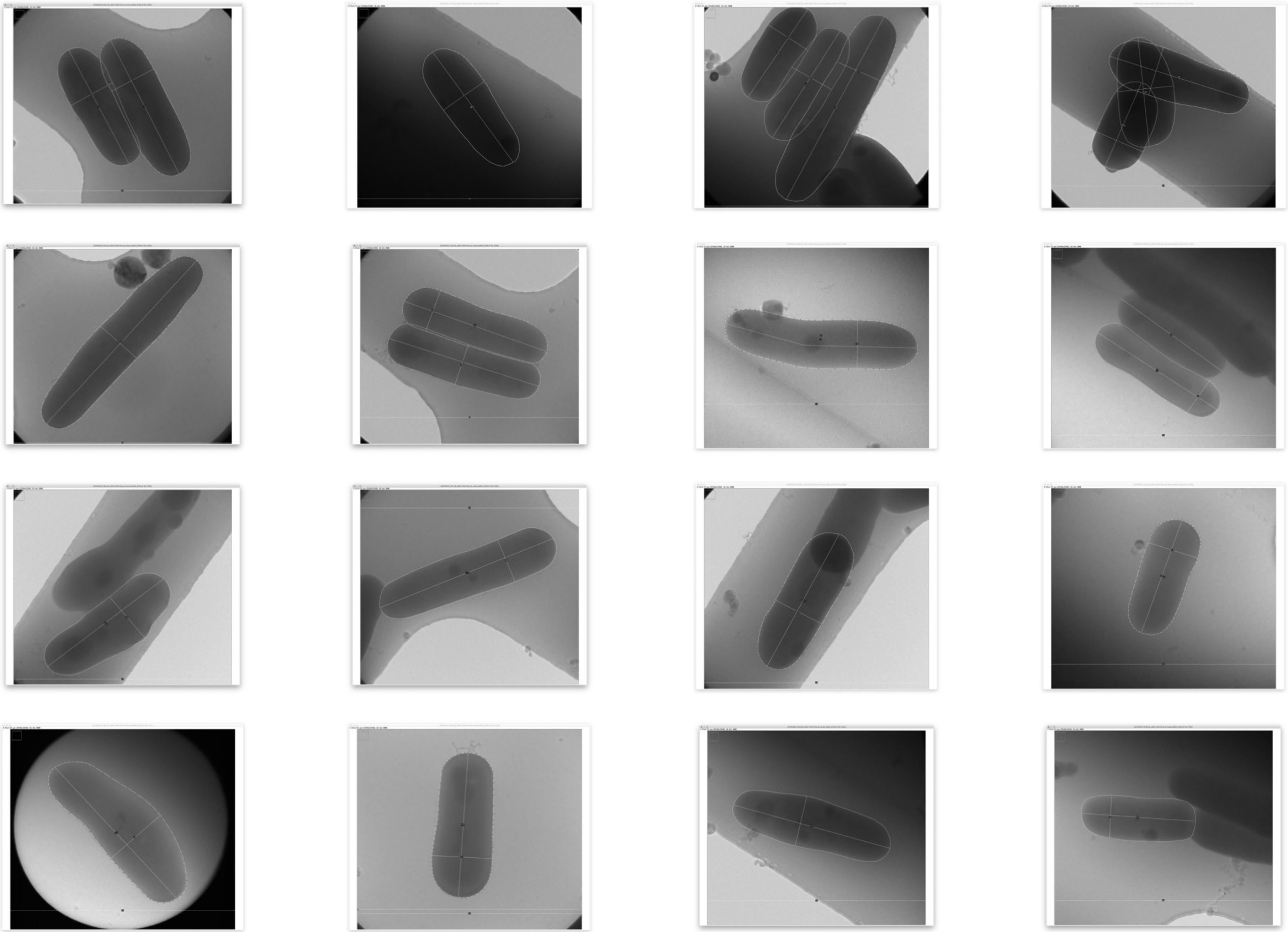

Mycobacterium bovis

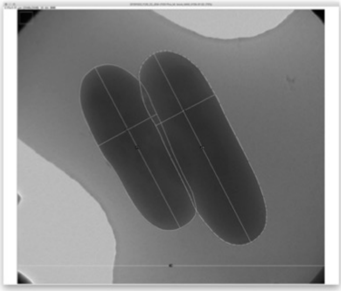
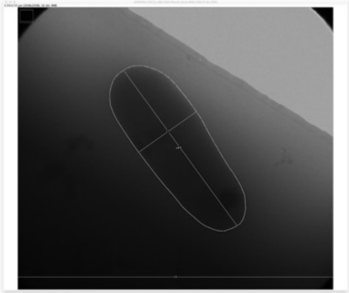
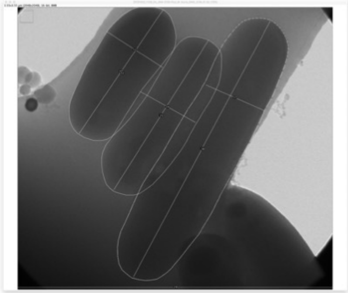
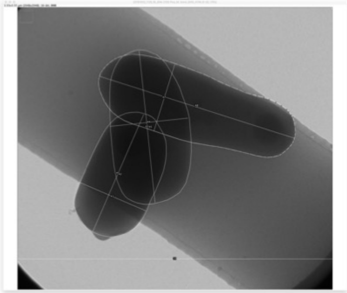
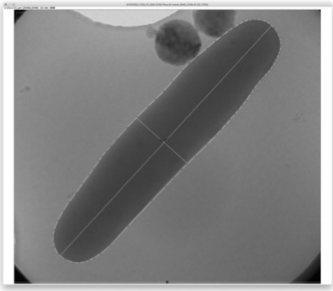
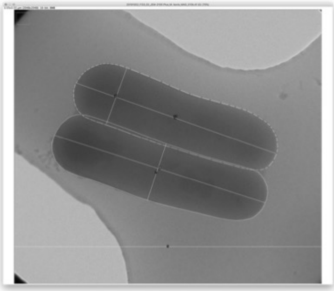
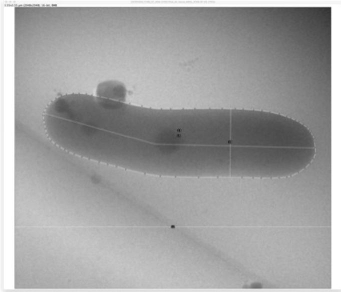
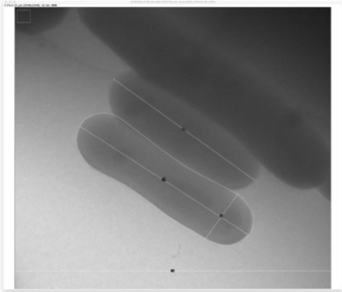
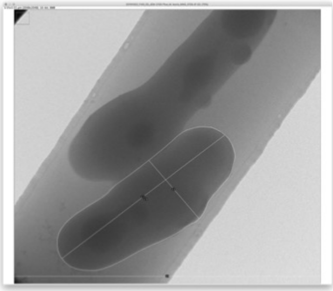
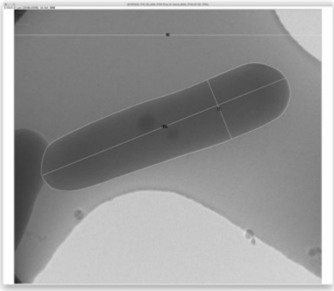
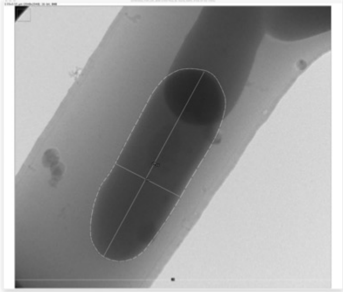
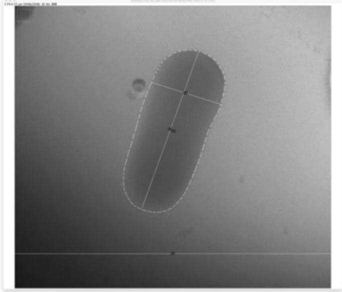
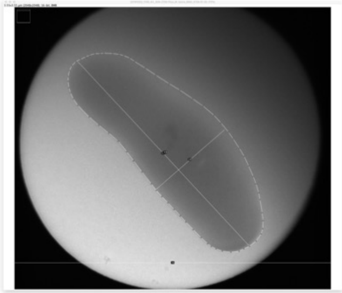
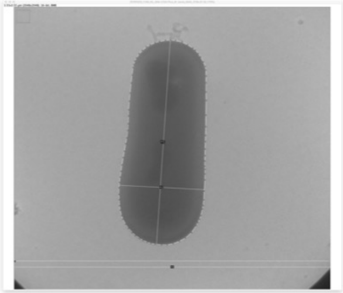
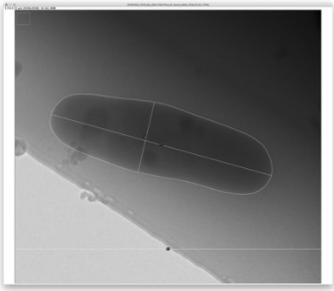
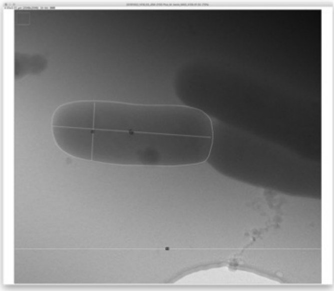

Mycobacterium bovis

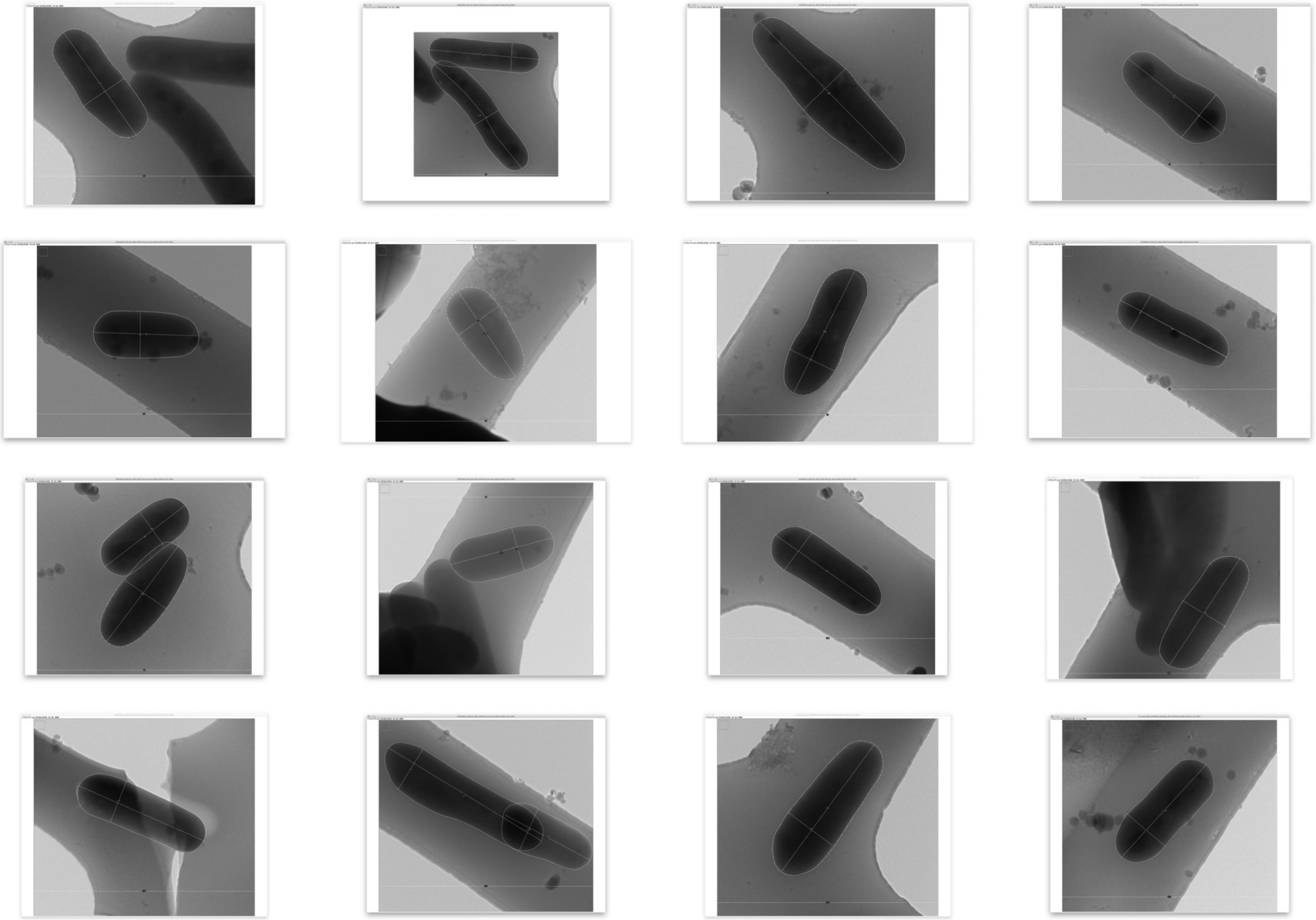

Mycobacterium bovis

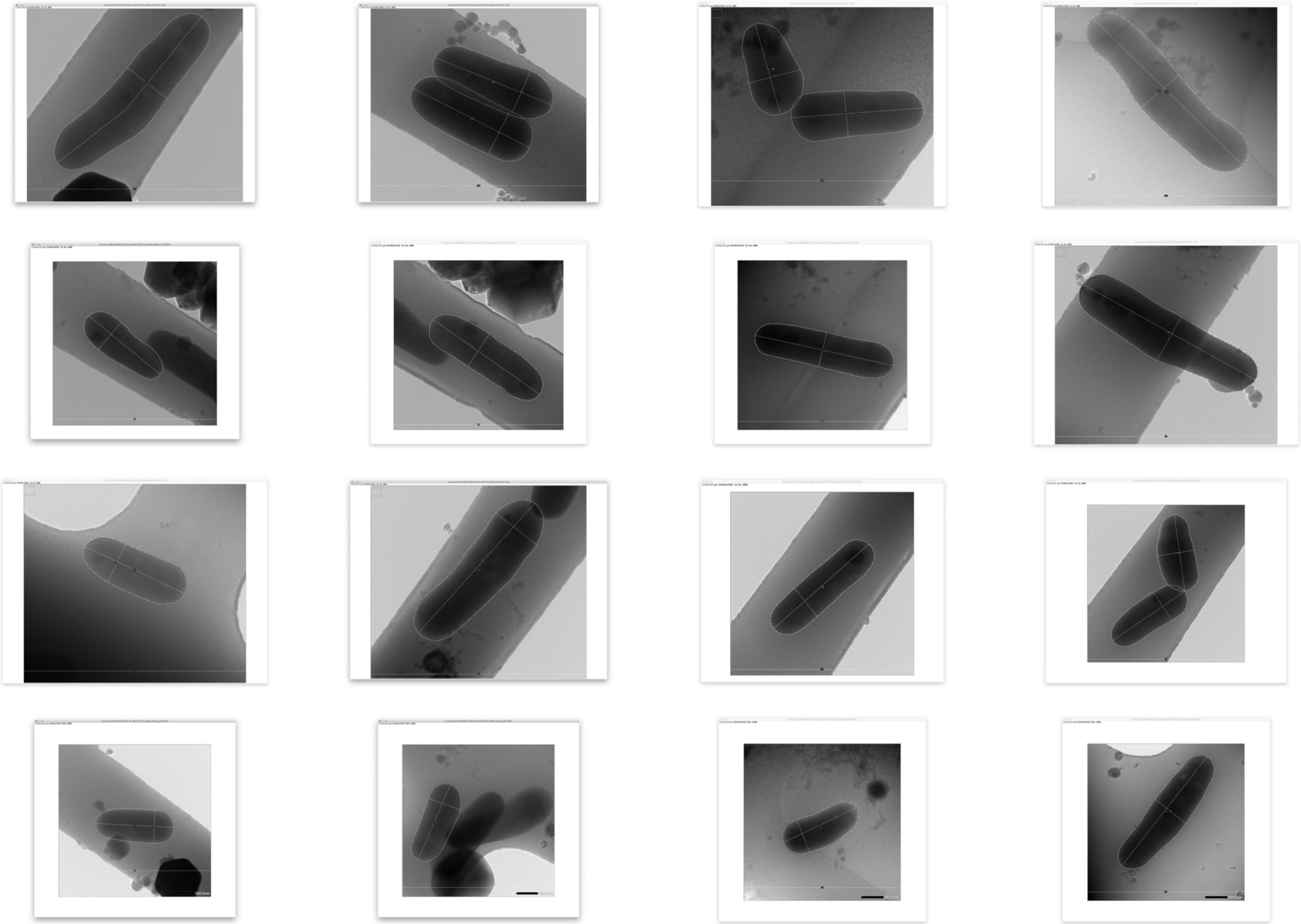

Mycobacterium bovis

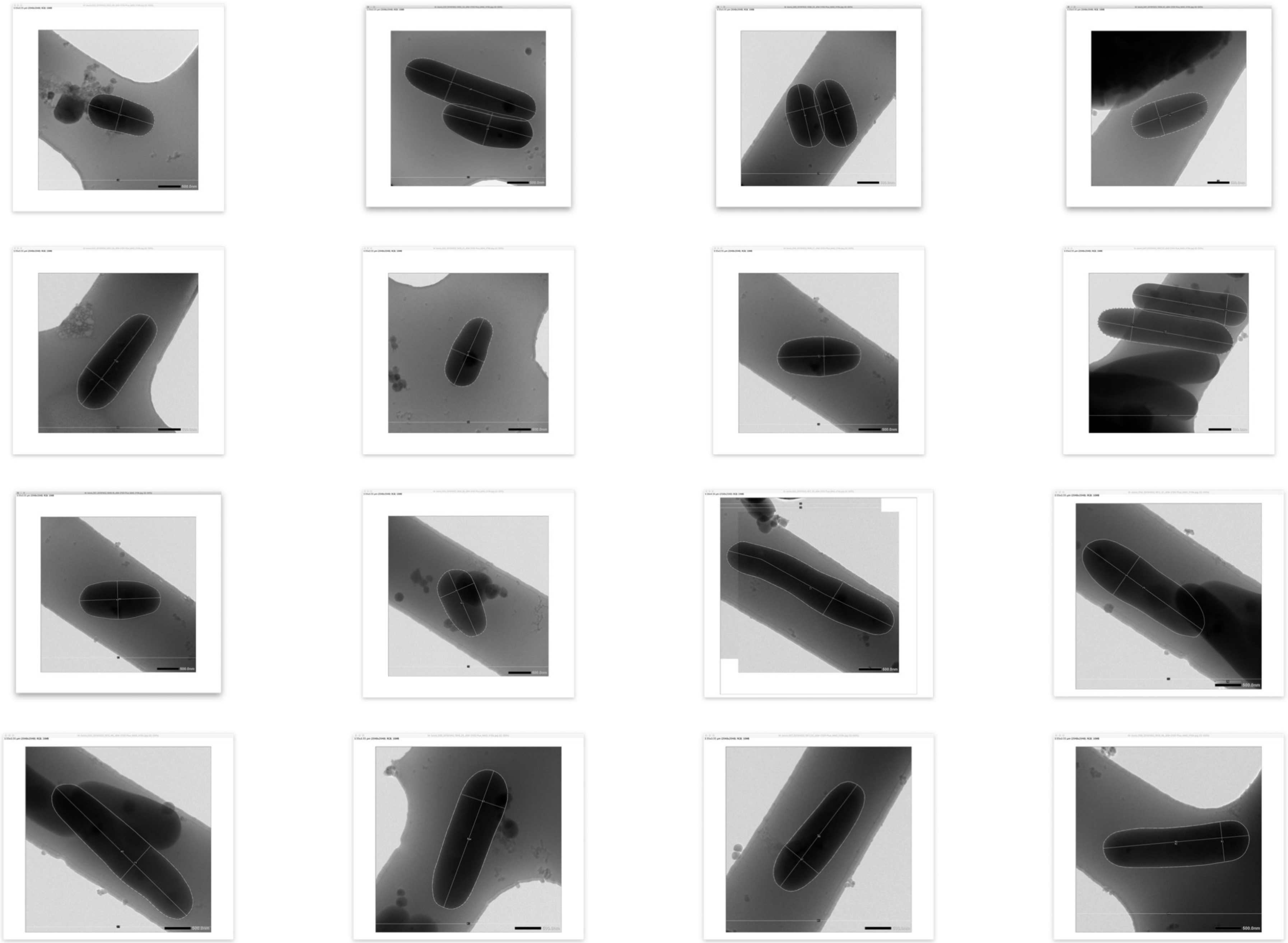

Mycobacterium bovis

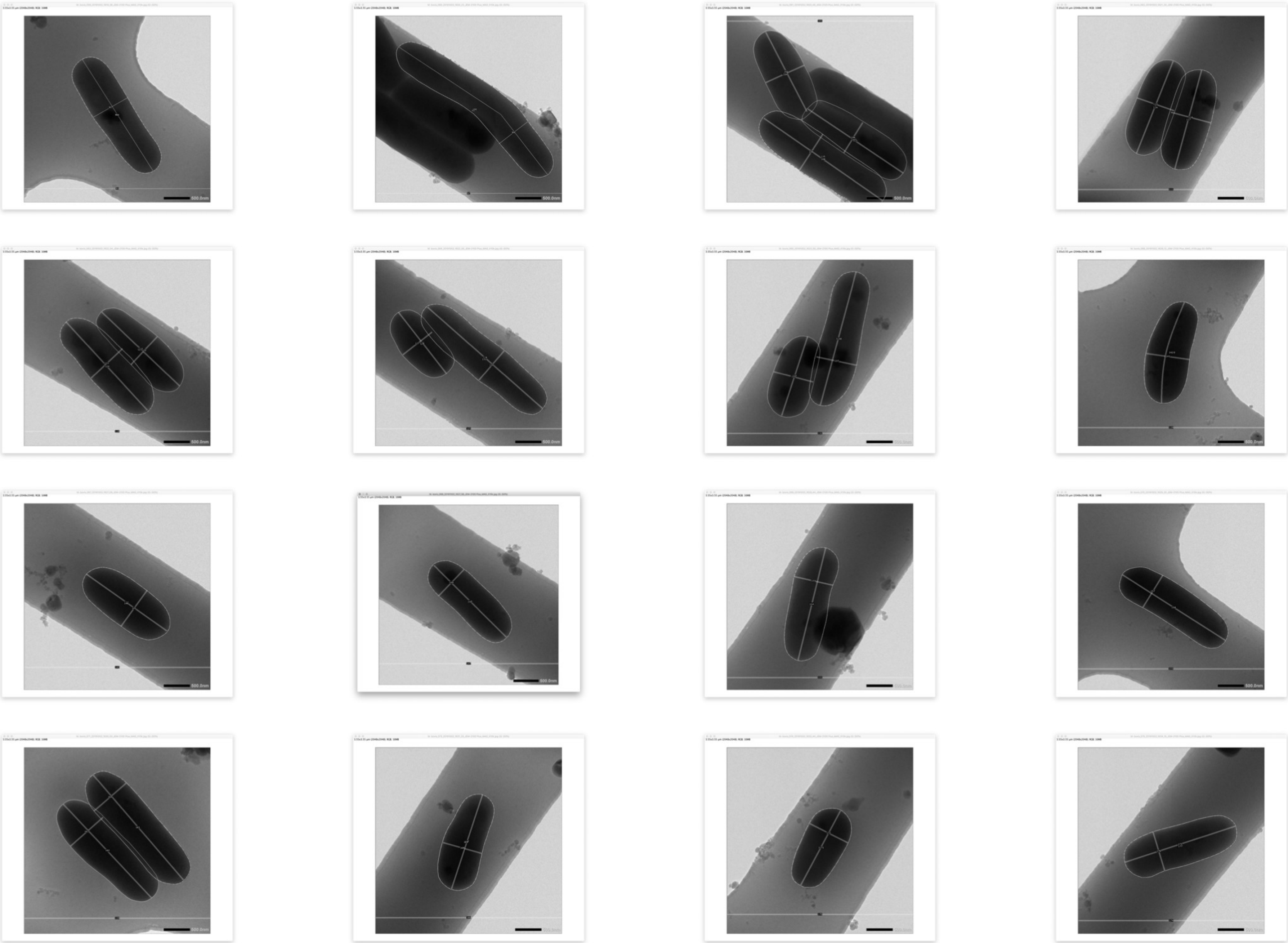

Mycobacterium bovis

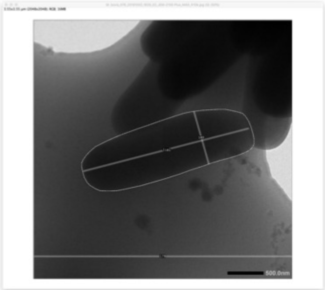

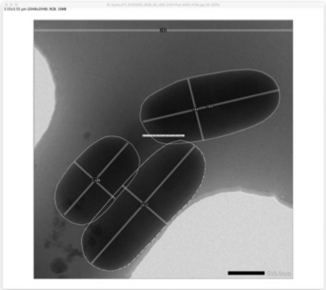

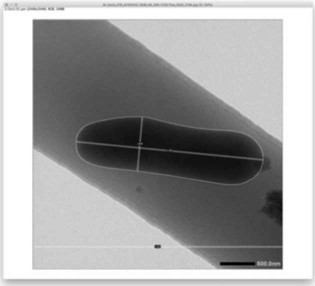

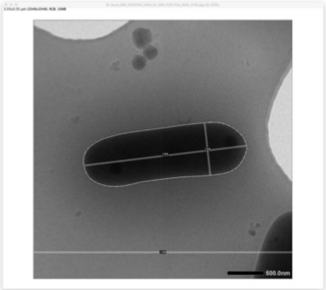

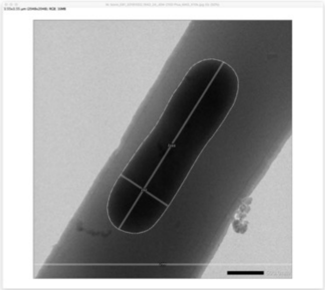

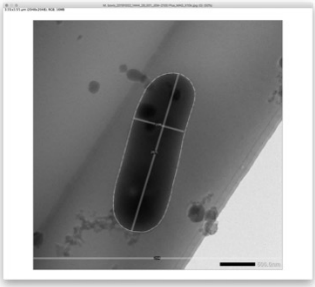

Mycobacterium microti

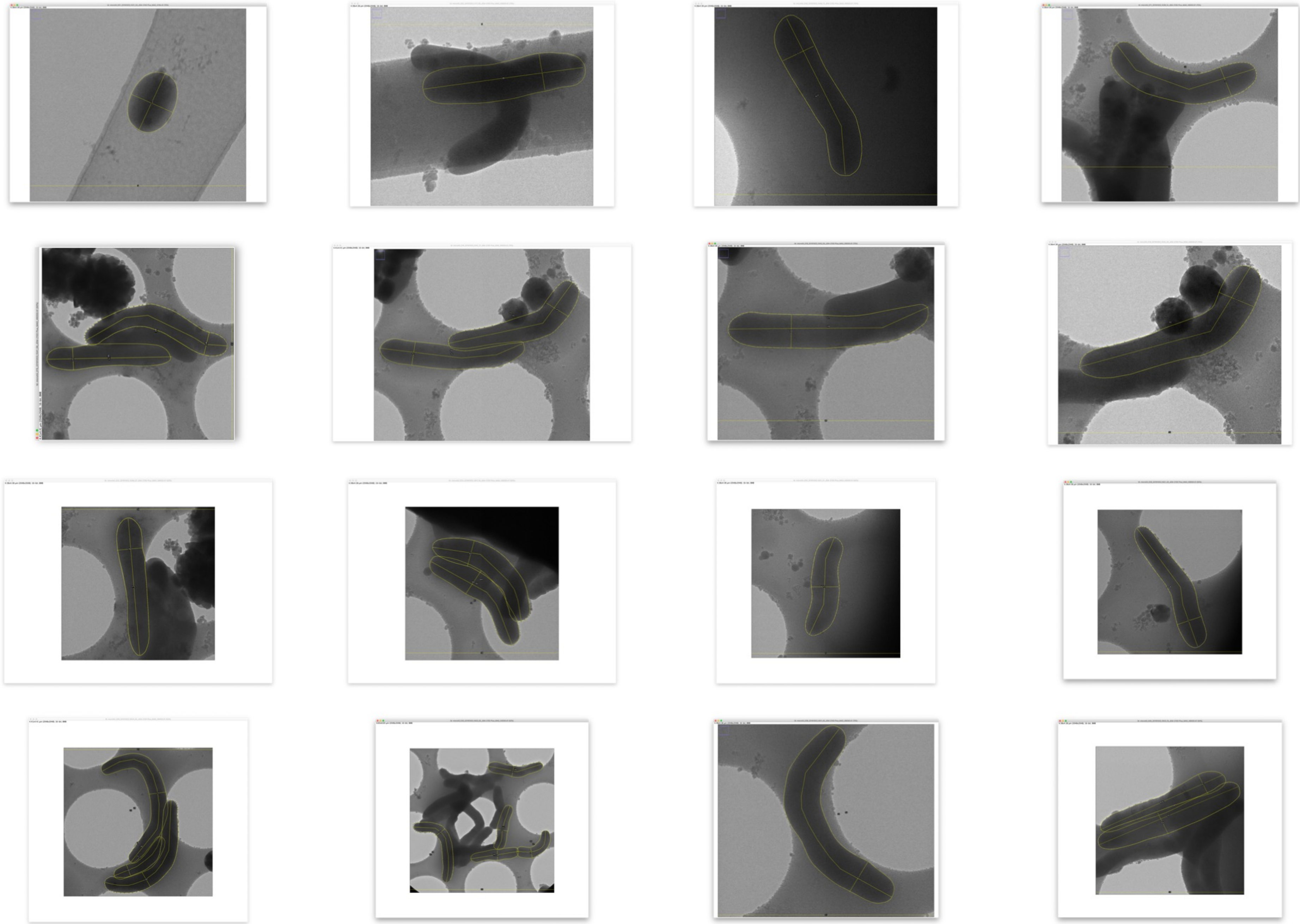

Mycobacterium microti

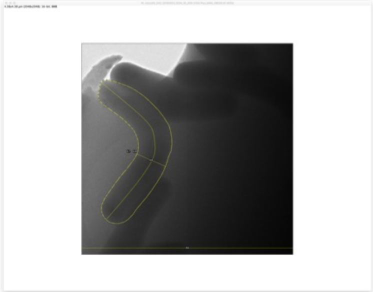

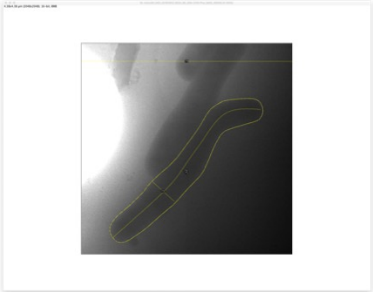

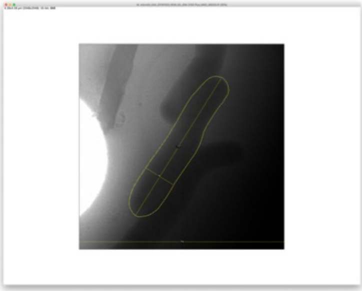

Mycobacterium avium

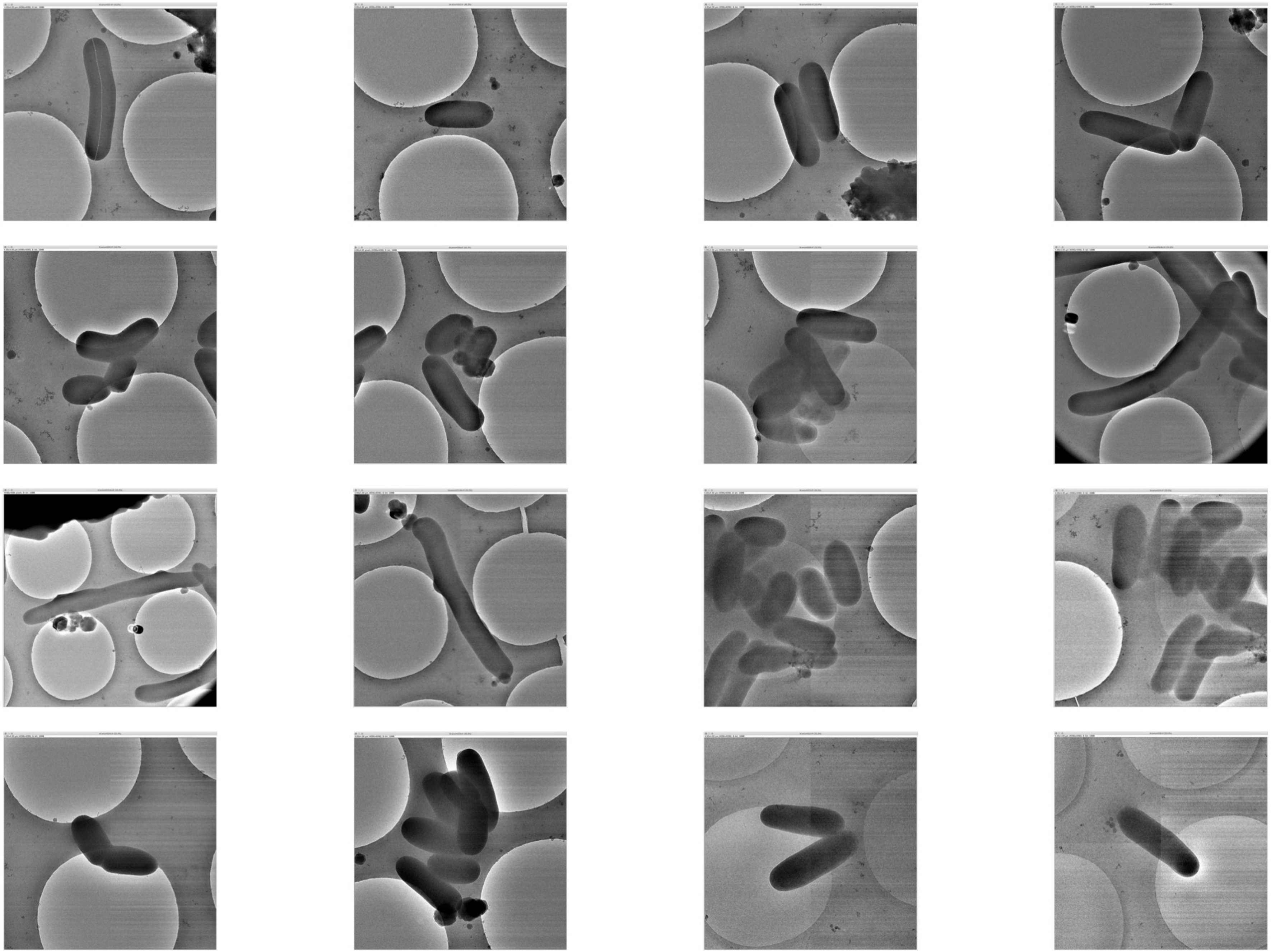

Mycobacterium avium

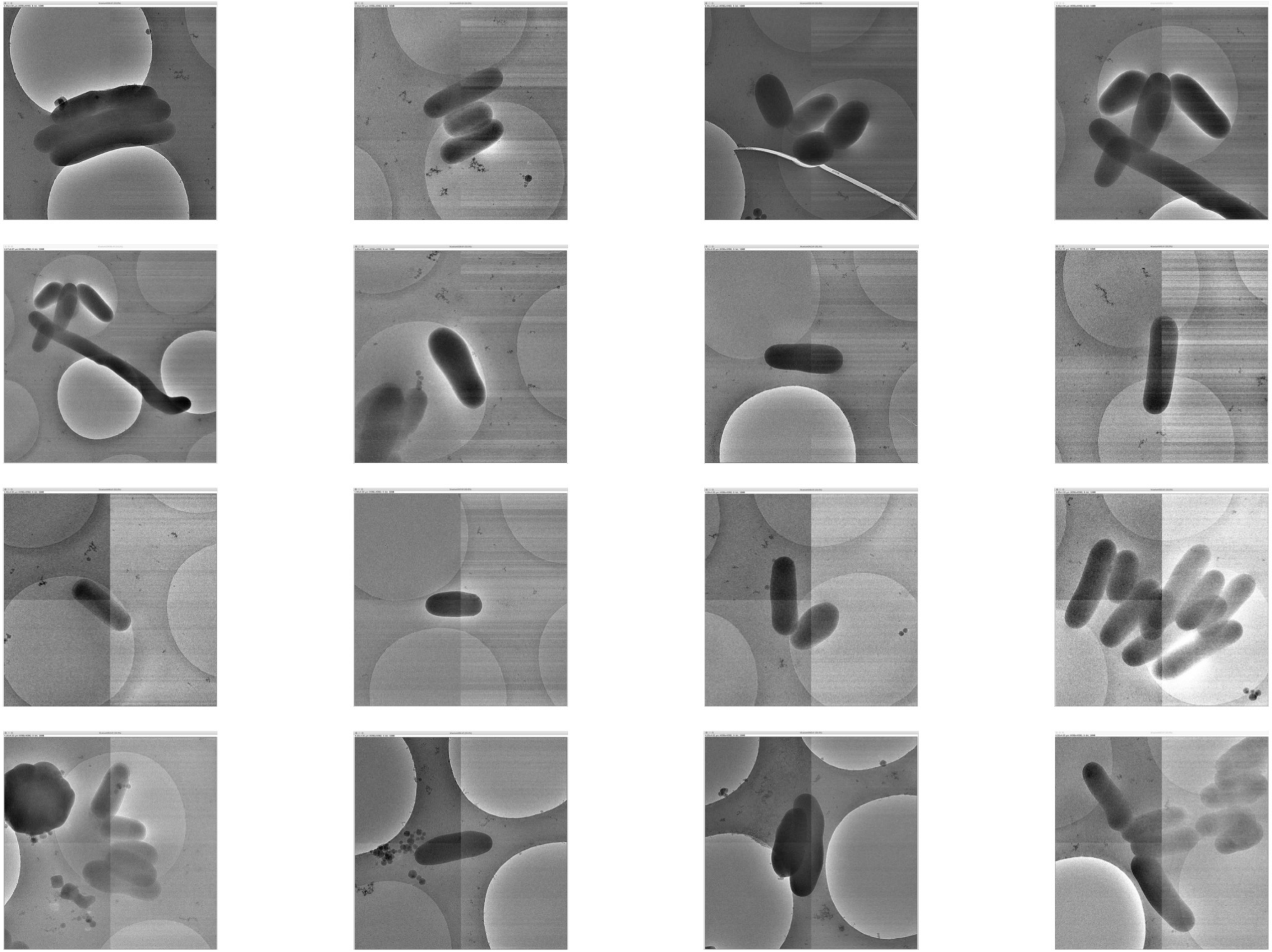

Mycobacterium avium

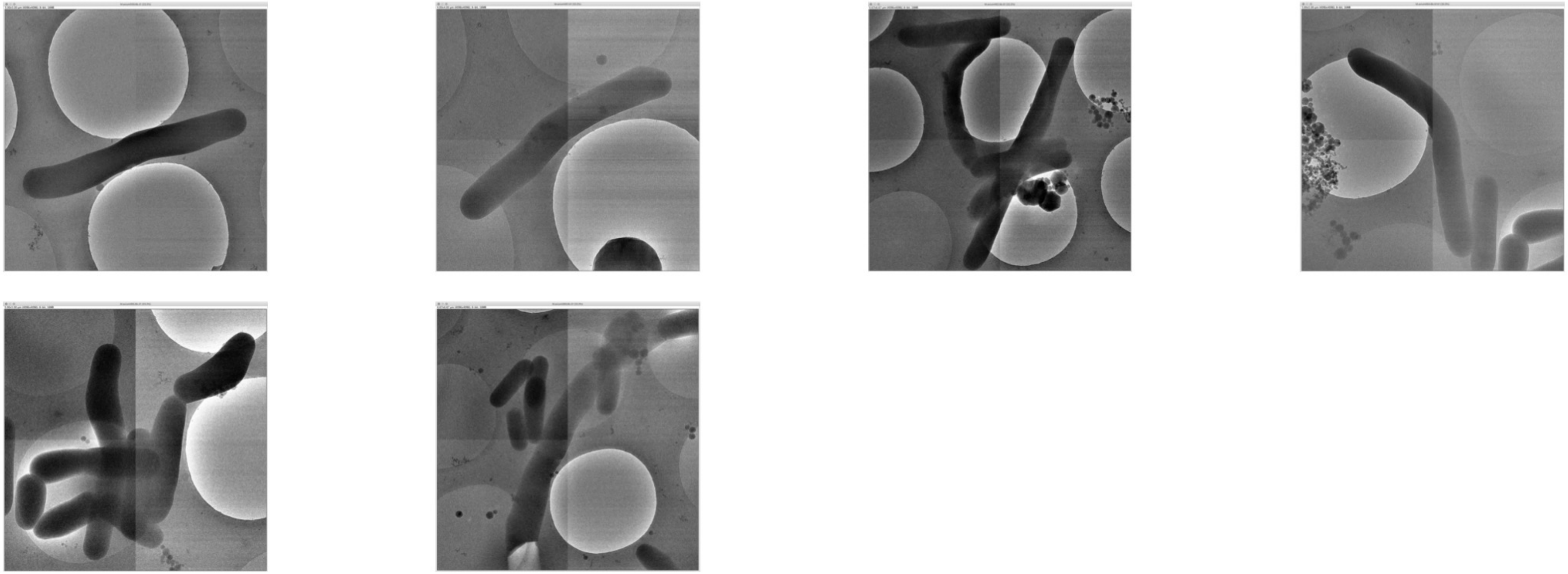

***Mycobacterium celatum* (ATCC 51130)**

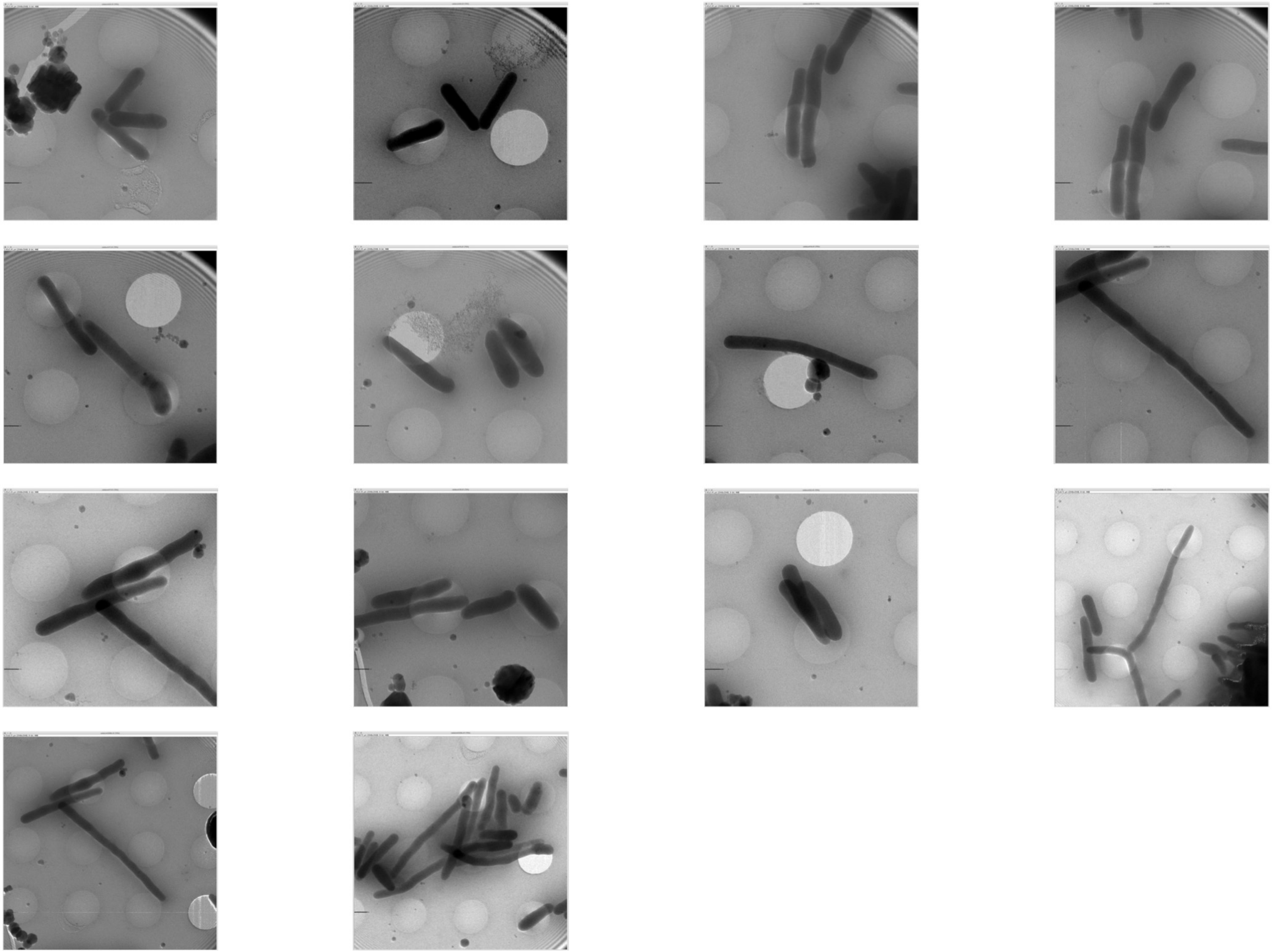

Mycobacterium celatum (ATCC 51131)

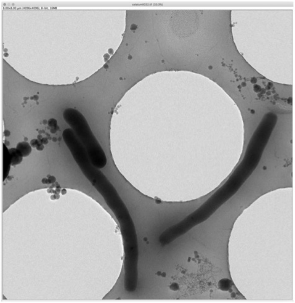

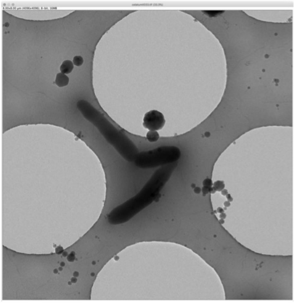

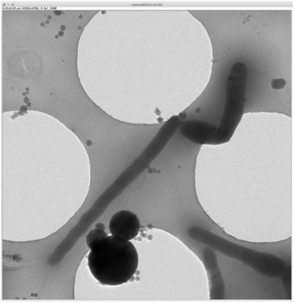

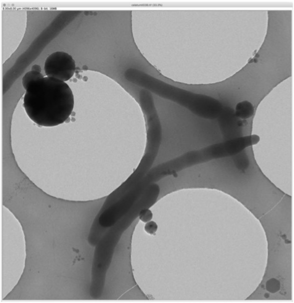

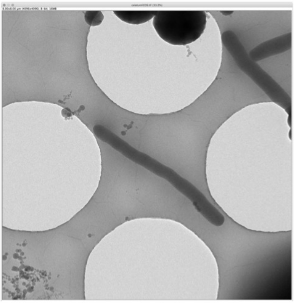

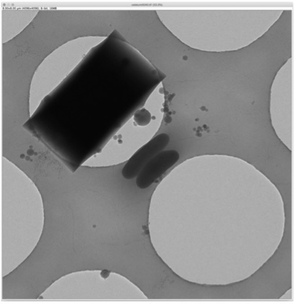

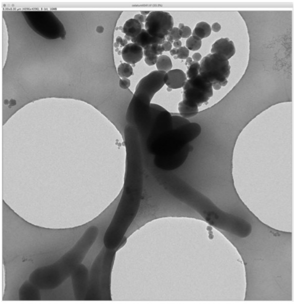

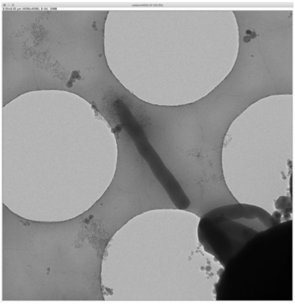

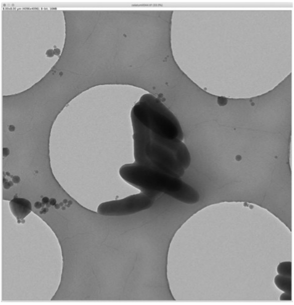

***Mycobacterium celatum* (ATCC 51131)**

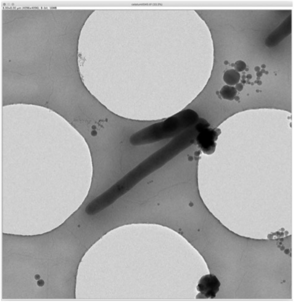

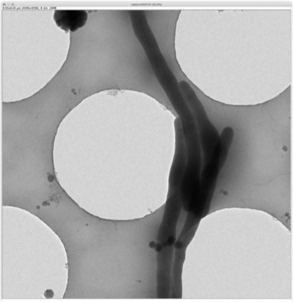

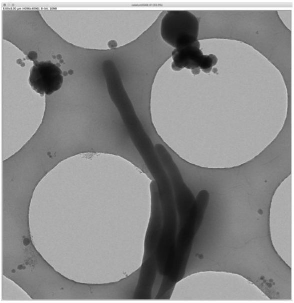

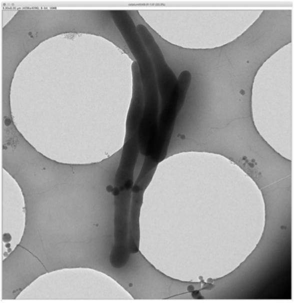

Mycobacterium gordonae

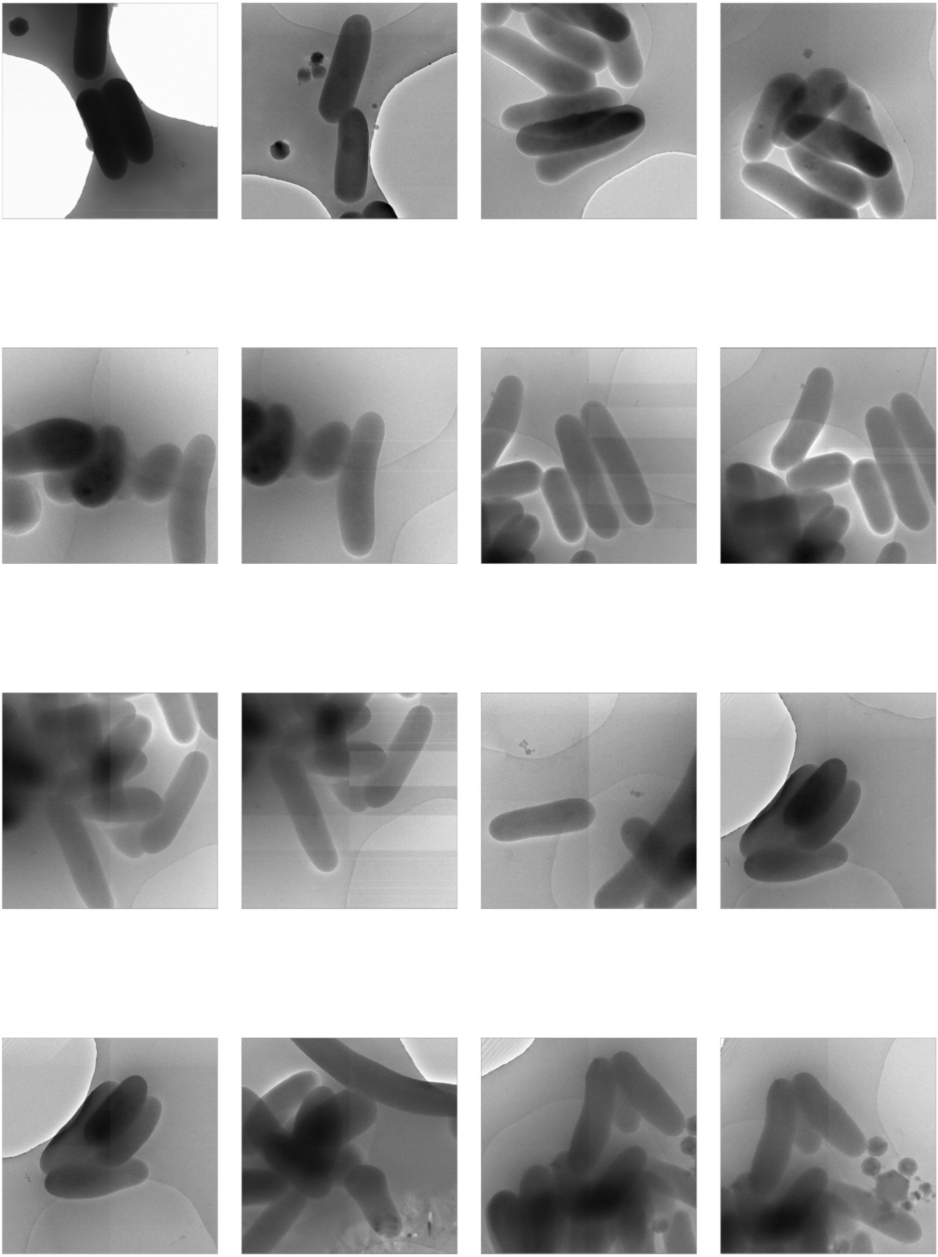

Mycobacterium gordonae

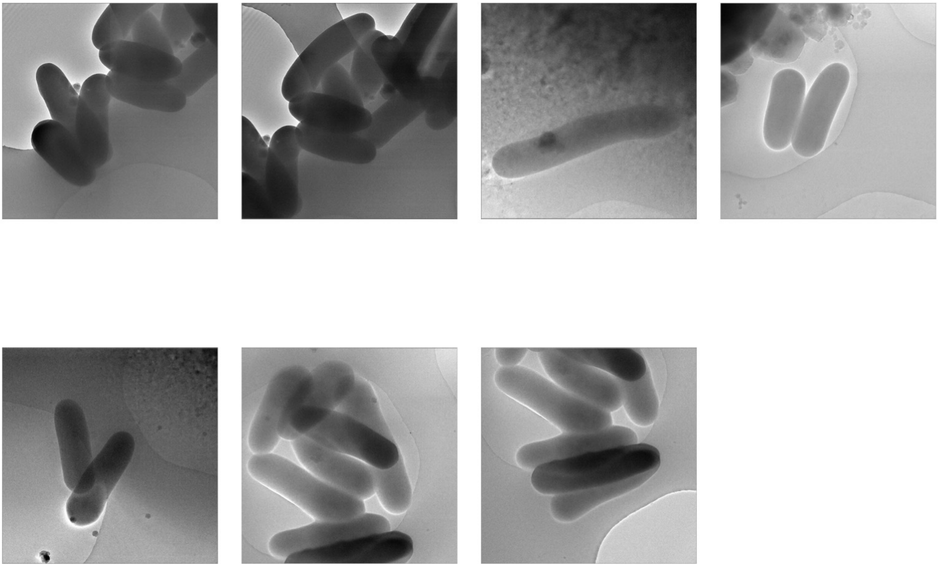

Mycobacterium intermedium

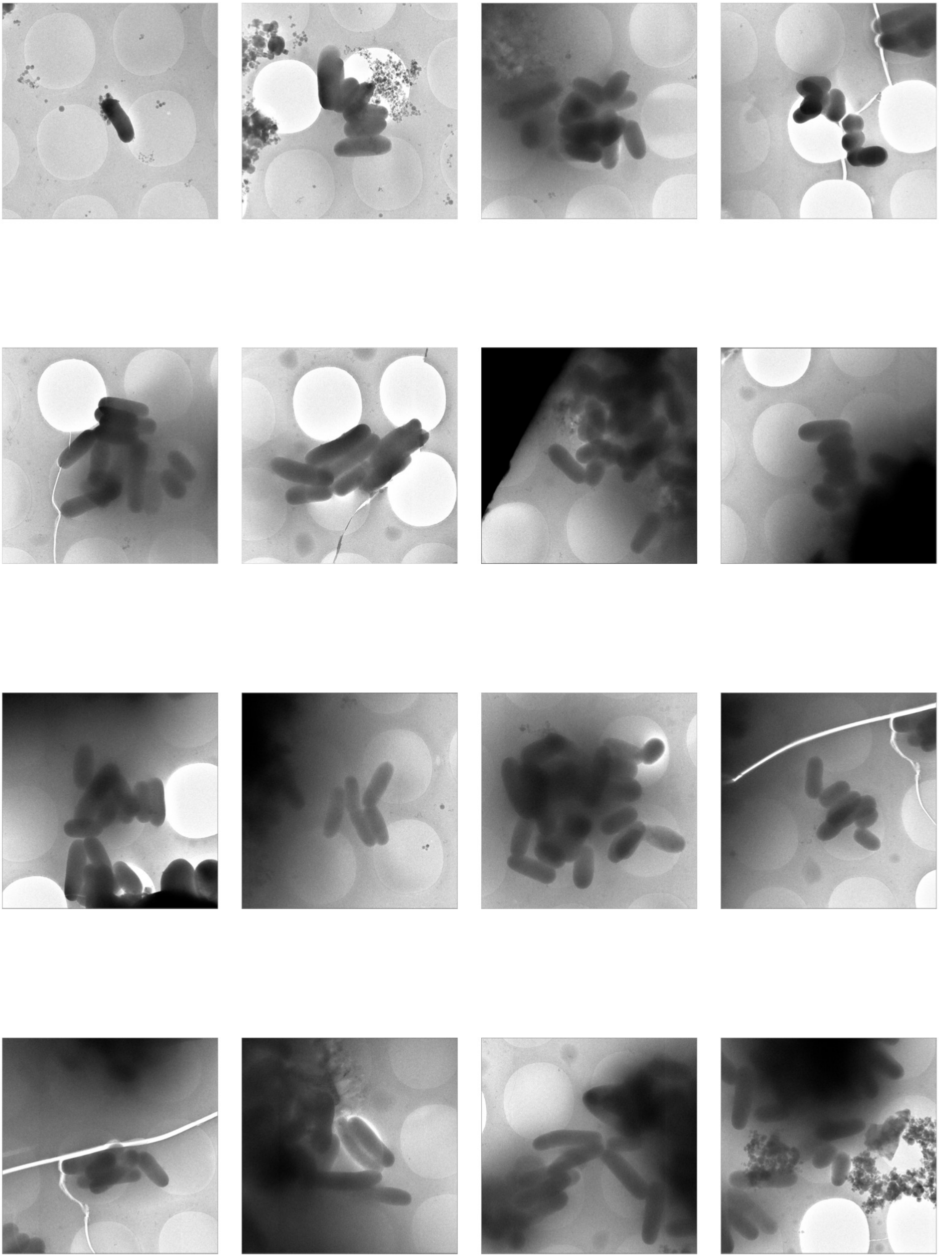

Mycobacterium intermedium

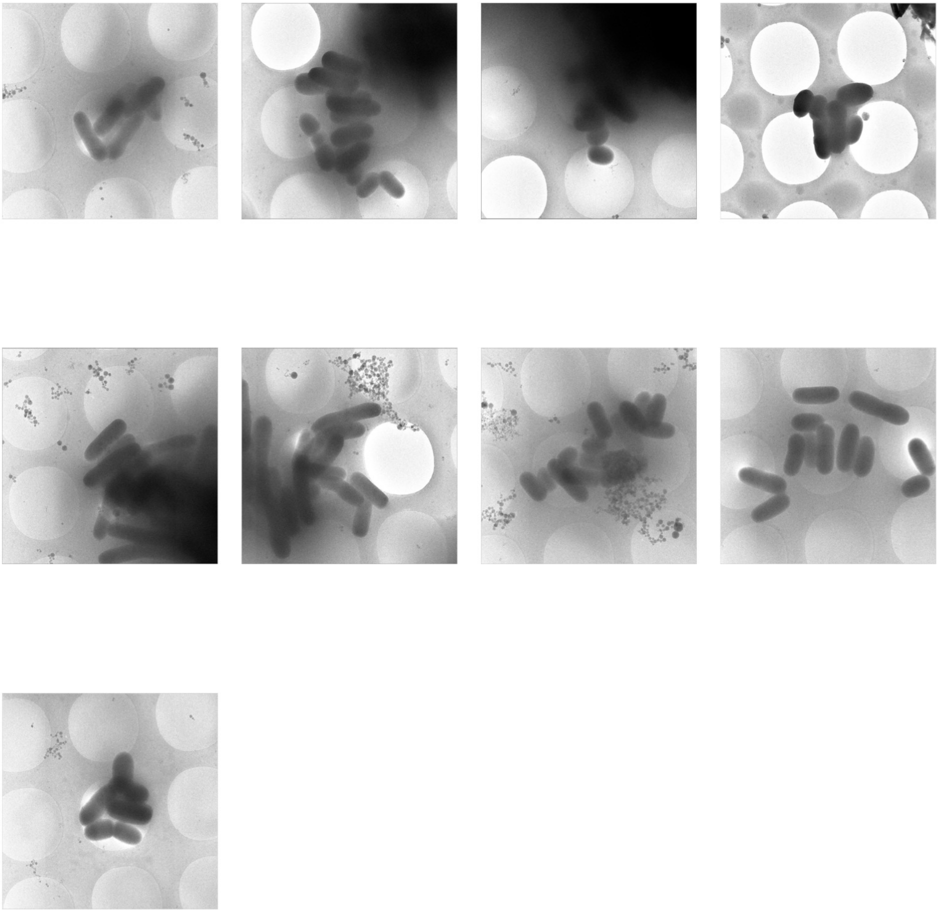

Mycobacterium intracellulare

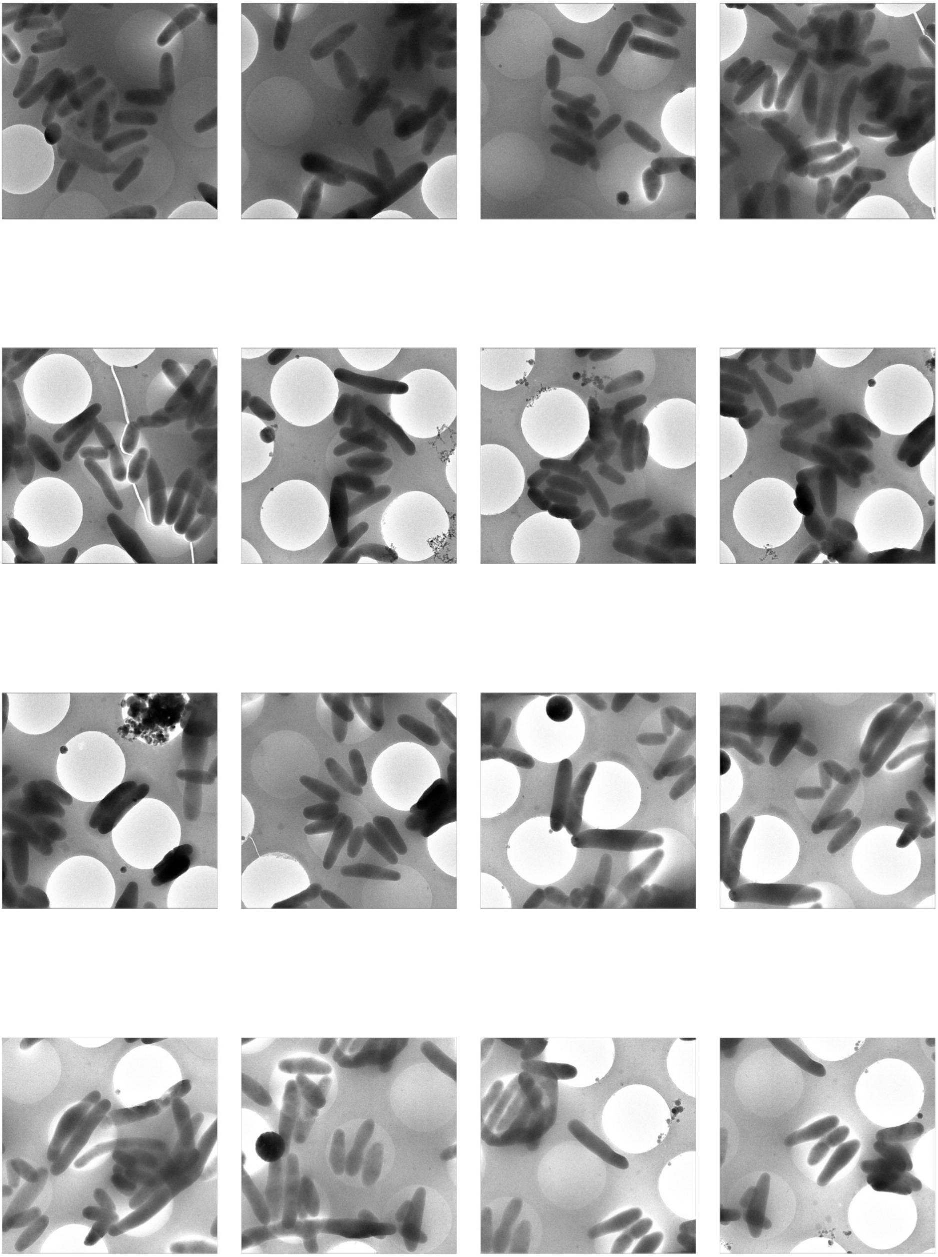

Mycobacterium intracellulare

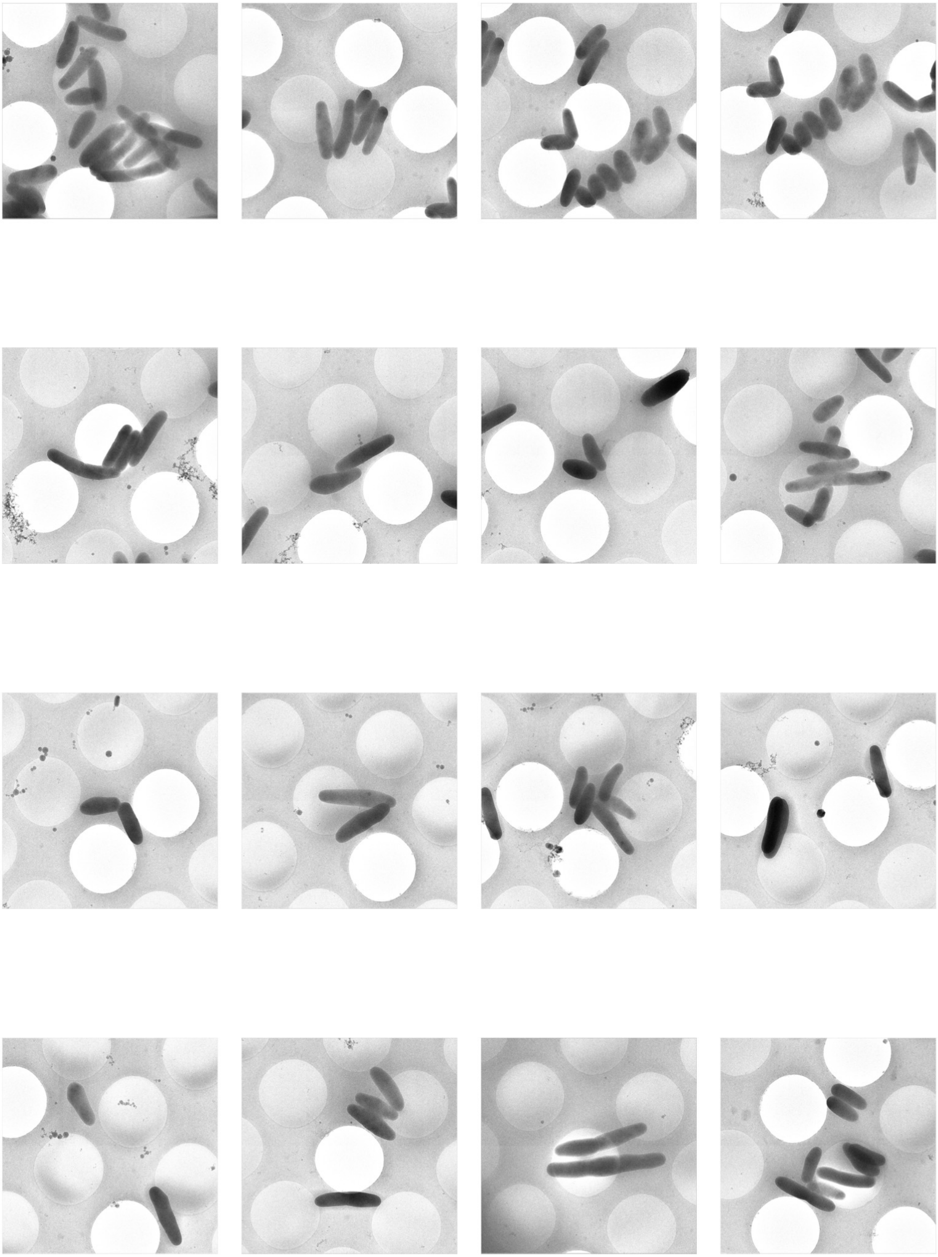

Mycobacterium intracellulare

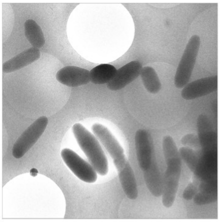

Mycobacterium marinum

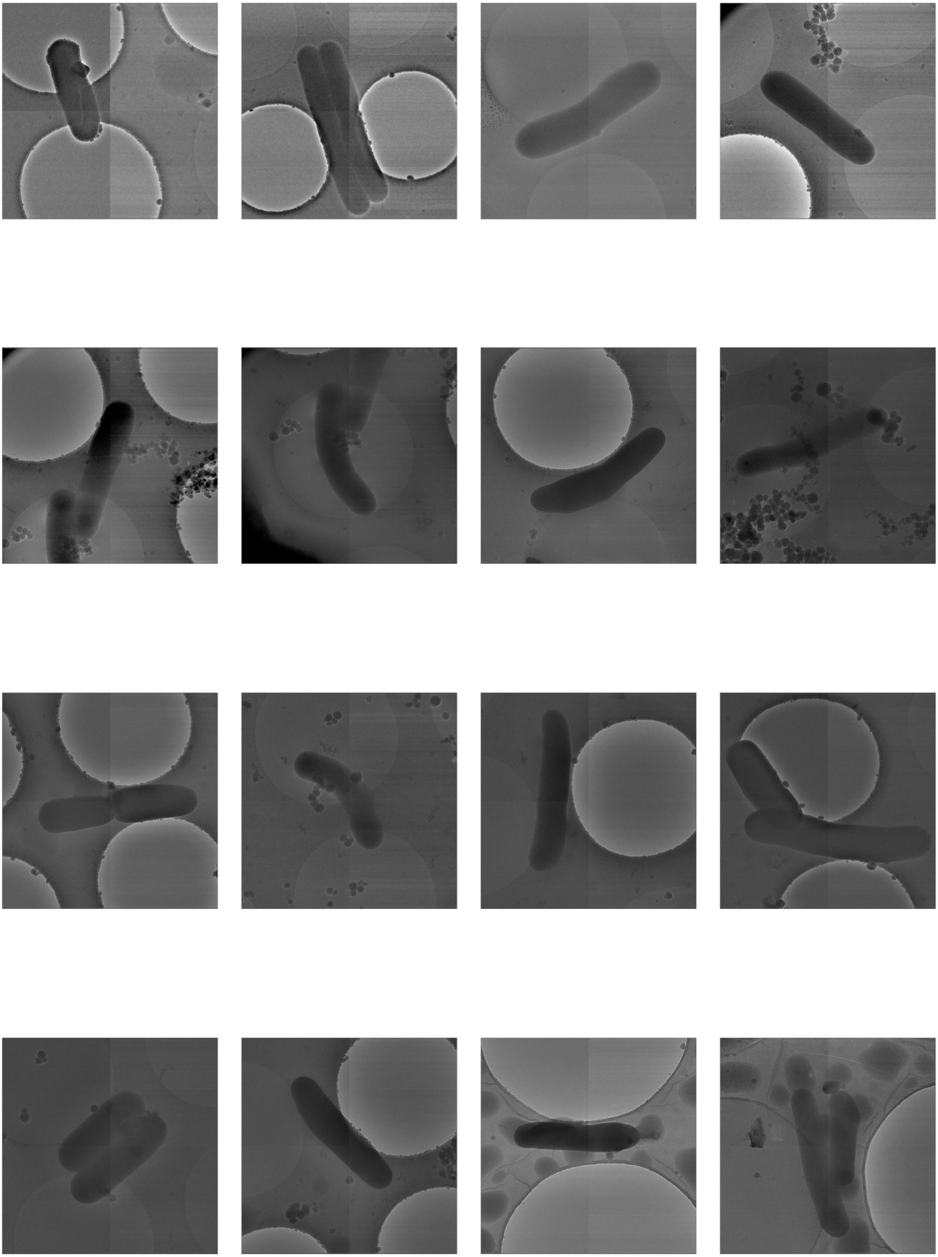

Mycobacterium marinum

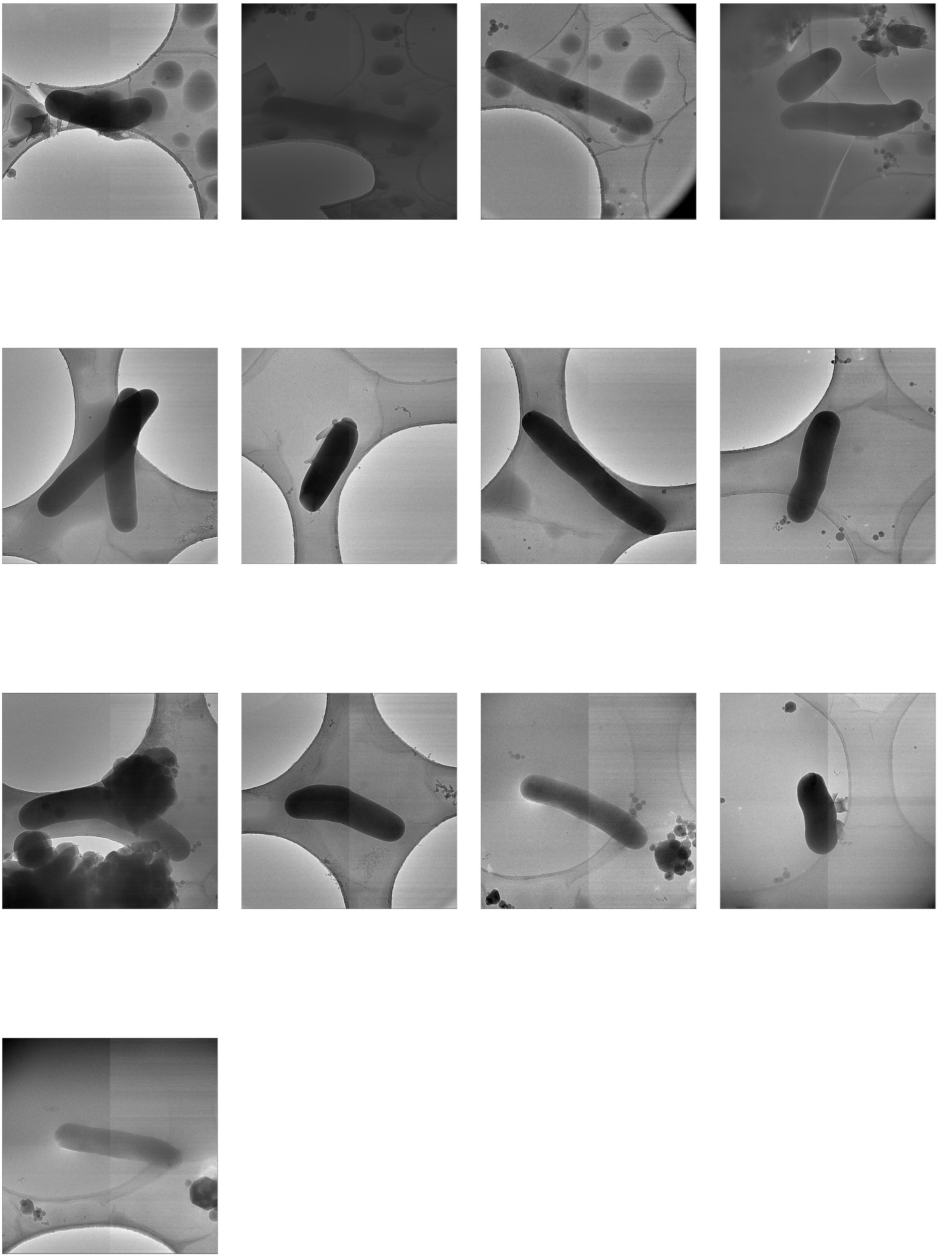

Mycobacterium scrofulaceum

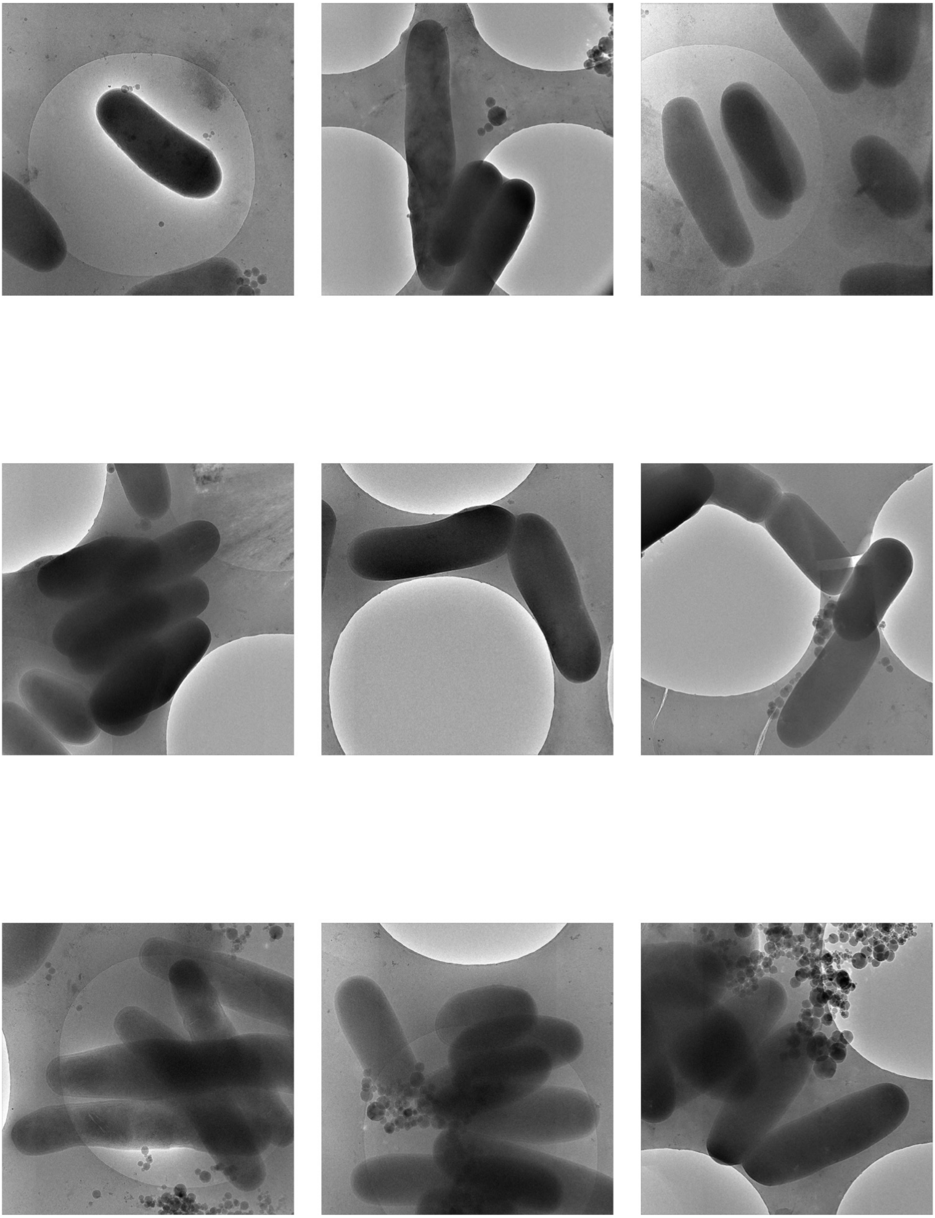

Mycobacterium scrofulaceum

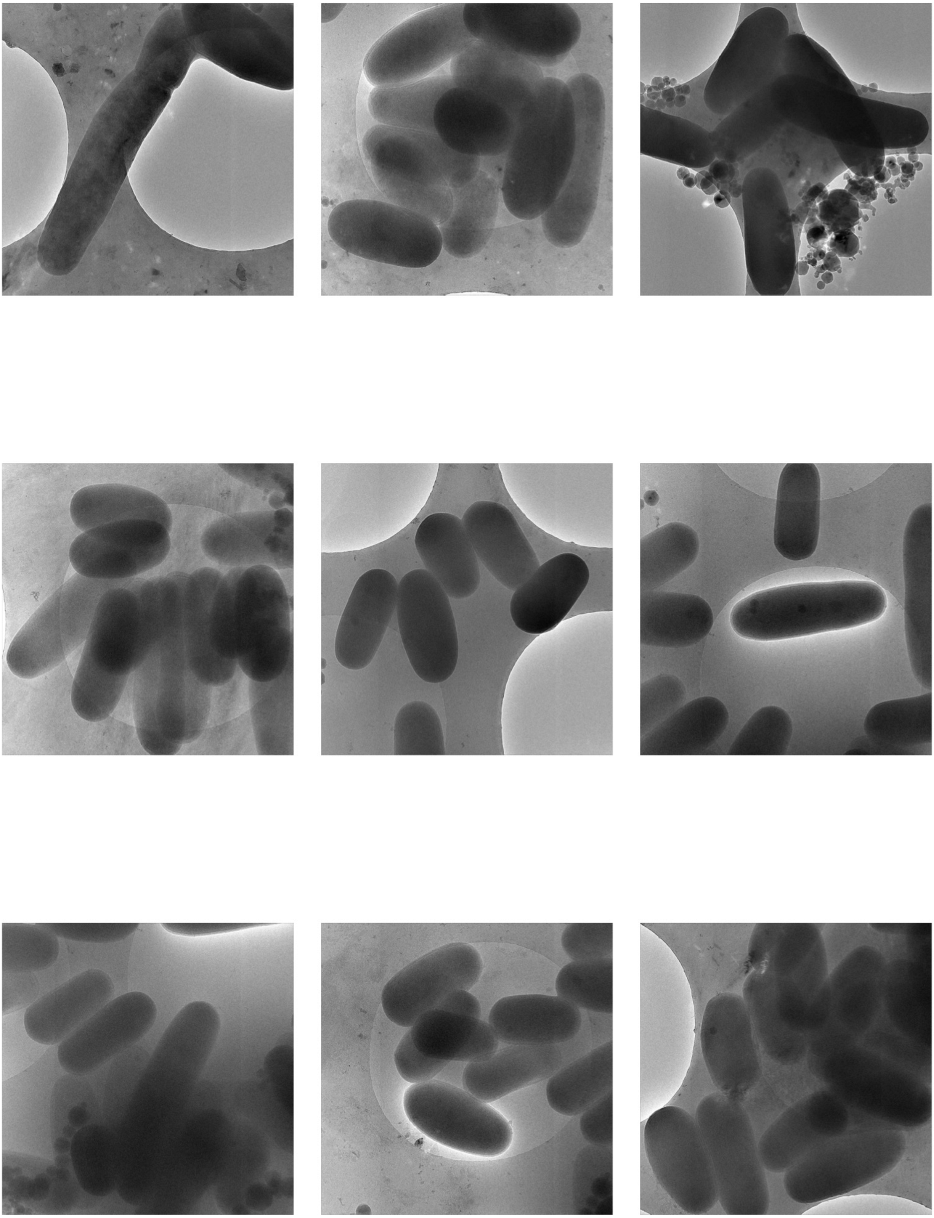

Mycobacterium scrofulaceum

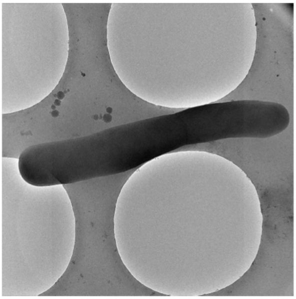
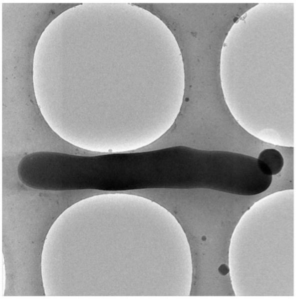
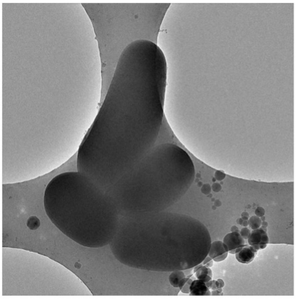
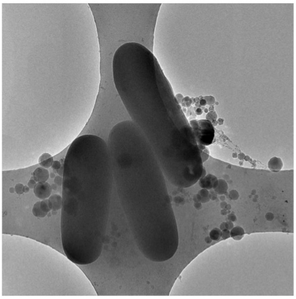

Mycobacterium ulcerans

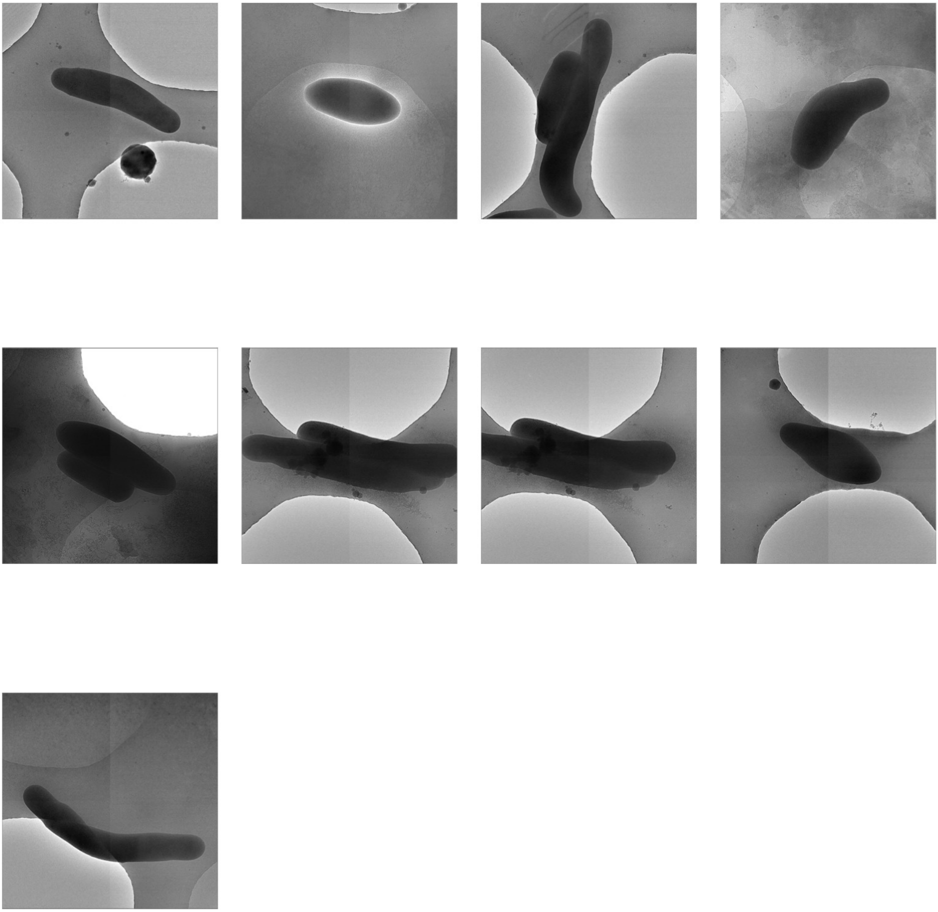

Mycobacterium xenopi

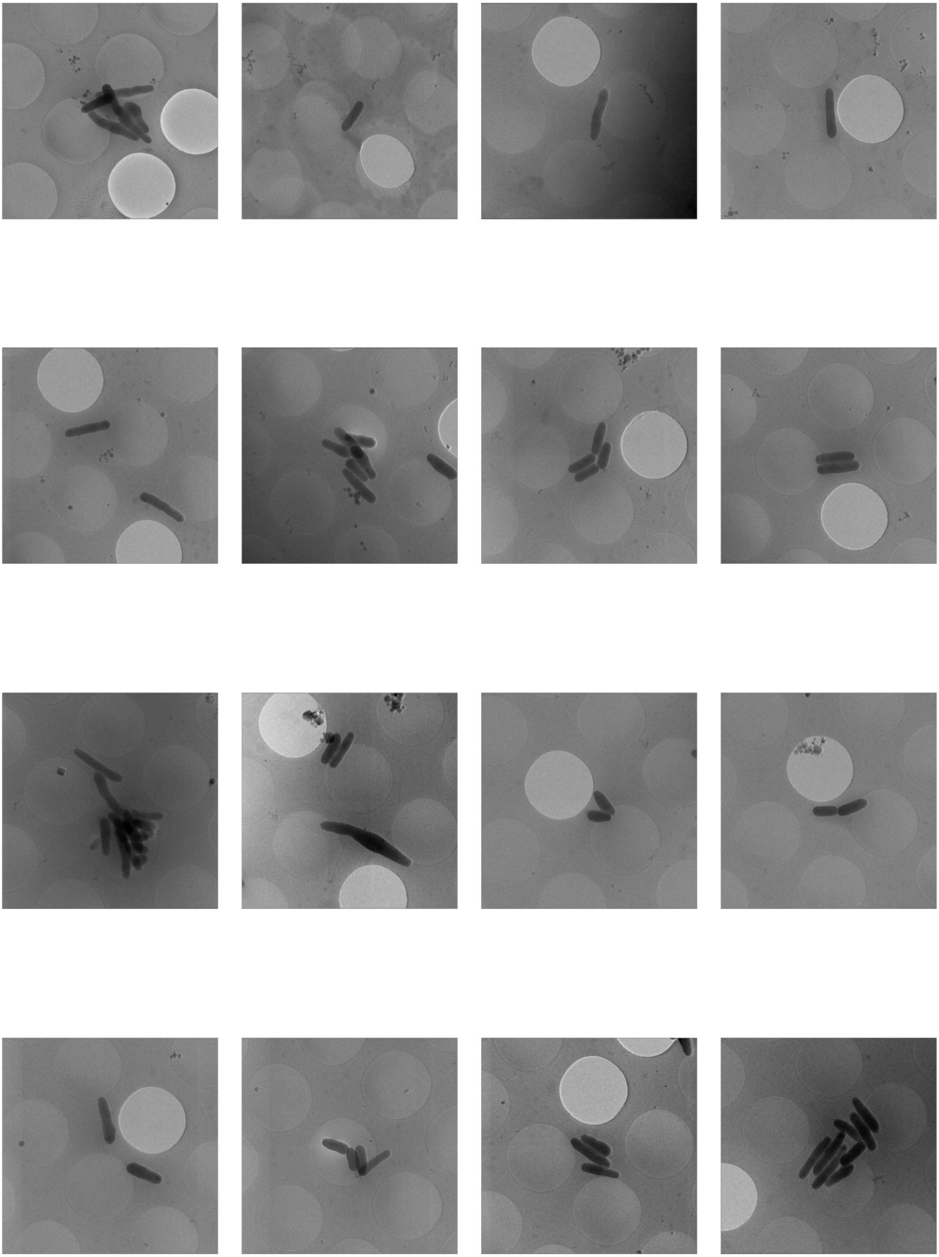

Mycobacterium xenopi

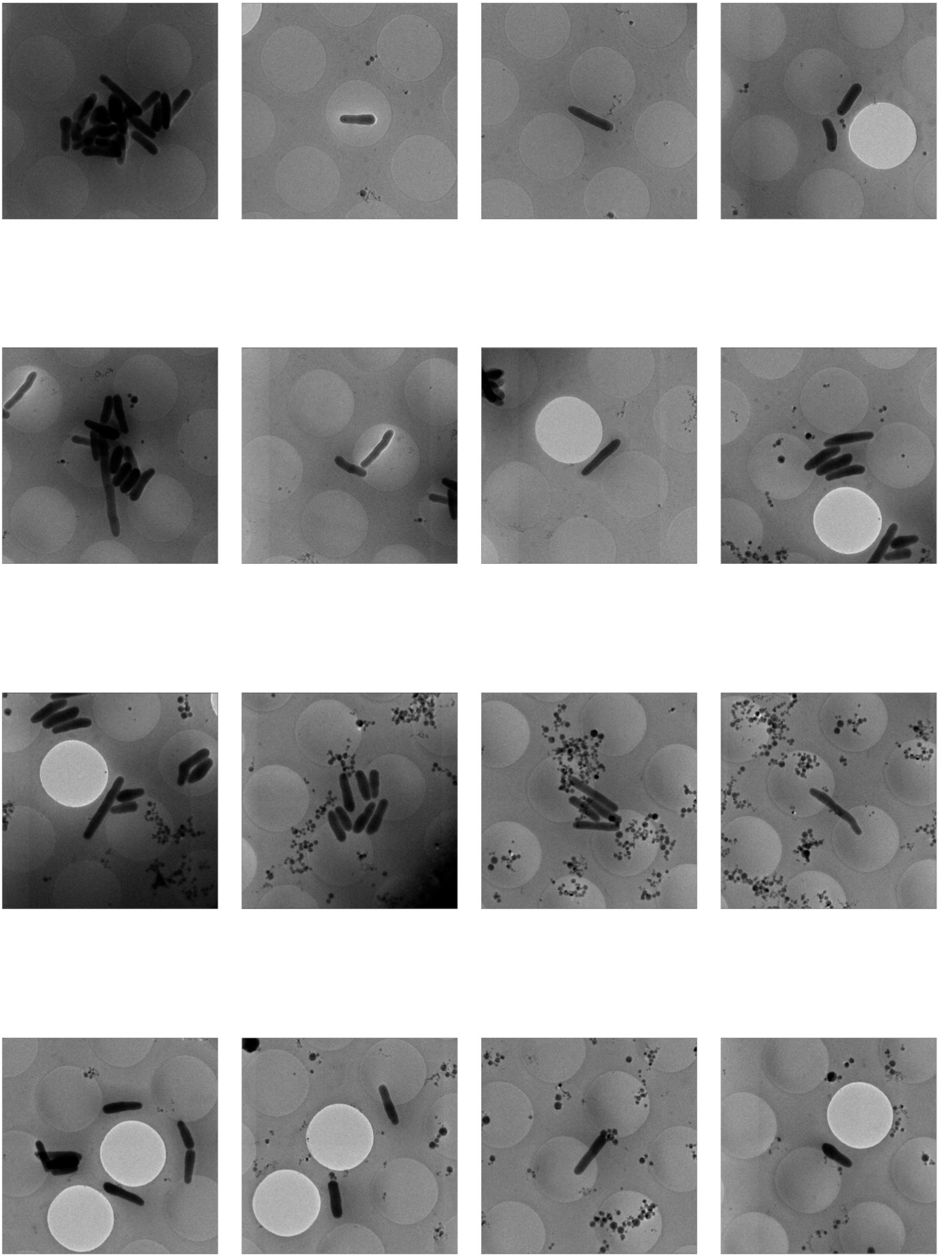

Mycobacterium xenopi

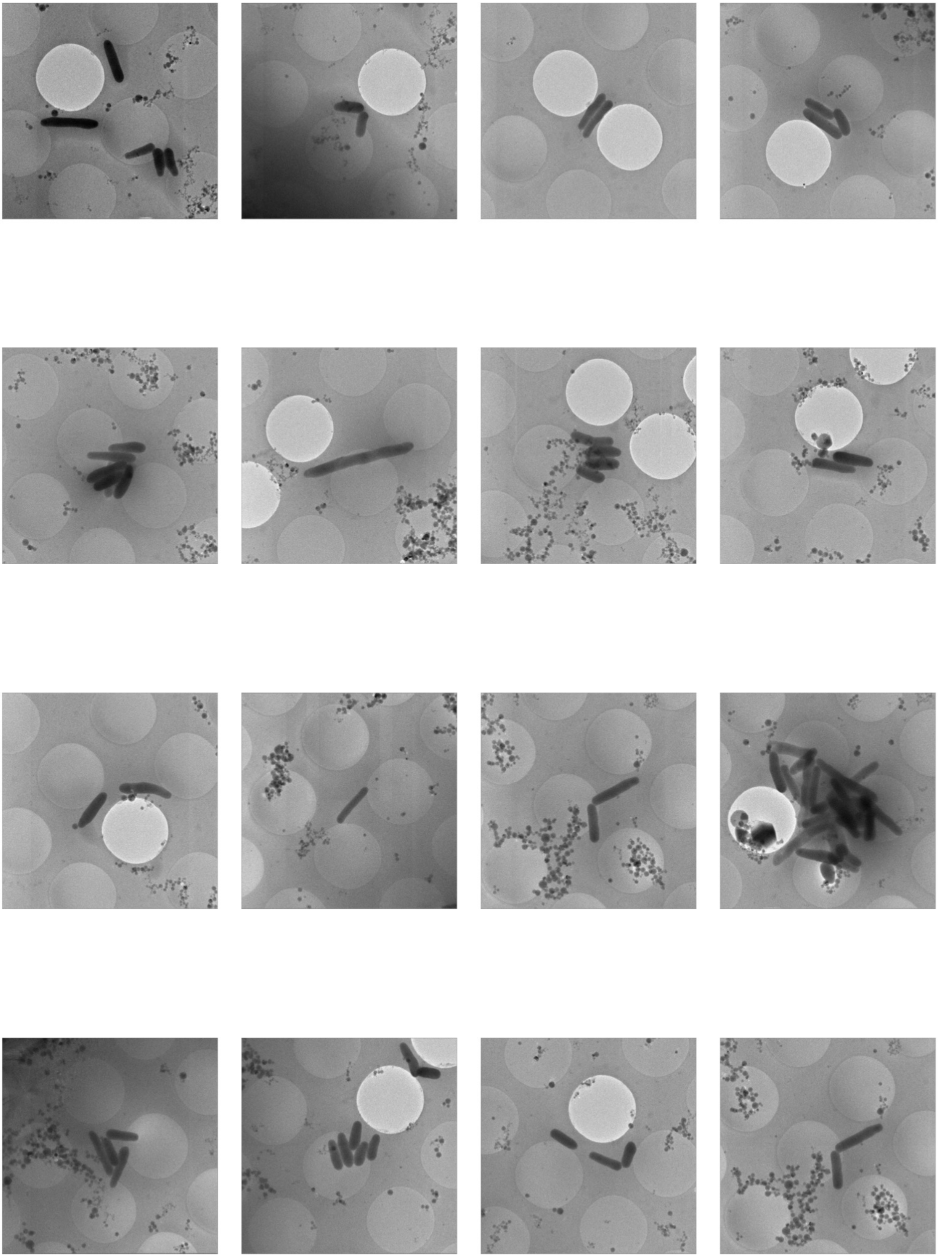

Mycobacterium xenopi

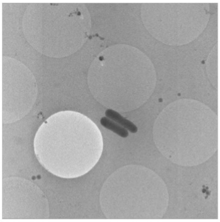

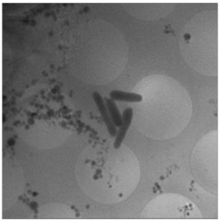

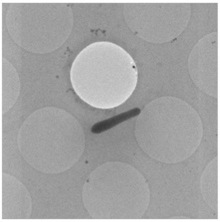

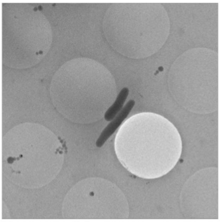

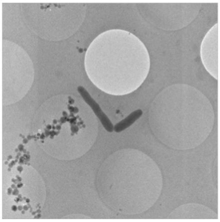

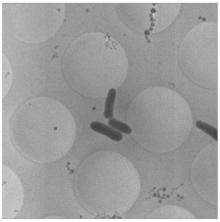

Supplement: Supplementary file 6 [file Data_Sheet_6.PDF]
